# Promoting the Furan Ring‐Opening Reaction to Access New Donor–Acceptor Stenhouse Adducts with Hexafluoroisopropanol

**DOI:** 10.1002/anie.202100115

**Published:** 2021-03-18

**Authors:** Michèle Clerc, Friedrich Stricker, Sebastian Ulrich, Miranda Sroda, Nico Bruns, Luciano F. Boesel, Javier Read de Alaniz

**Affiliations:** ^1^ Empa Swiss Federal Laboratories for Materials Science and Technology Laboratory for Biomimetic Membranes and Textiles Lerchenfeldstrasse 5 9014 St. Gallen Switzerland; ^2^ Department of Chemistry and Biochemistry University of California Santa Barbara CA 93106 USA; ^3^ Department of Chemistry University of Fribourg 1700 Fribourg Switzerland; ^4^ Department of Pure and Applied Chemistry University of Strathclyde Glasgow G1 1XL UK

**Keywords:** donor–acceptor Stenhouse adducts, hexafluoroisopropanol, hydrogen bonds, photochromic materials, photoswitches, synthetic methods

## Abstract

Donor–acceptor Stenhouse adducts (DASAs) are visible‐light‐responsive photoswitches with a variety of emerging applications in photoresponsive materials. Their two‐step modular synthesis, centered on the nucleophilic ring opening of an activated furan, makes DASAs readily accessible. However, the use of less reactive donors or acceptors renders the process slow and low yielding, which has limited their development. We demonstrate here that 1,1,1,3,3,3‐hexafluoro‐2‐propanol (HFIP) promotes the ring‐opening reaction and stabilizes the open isomer, allowing greatly reduced reaction times and increased yields for known derivatives. In addition, it provides access to previously unattainable DASA‐based photoswitches and DASA–polymer conjugates. The role of HFIP and the photochromic properties of a set of new DASAs is probed using a combination of ^1^H NMR and UV/Vis spectroscopy. The use of sterically hindered, electron‐poor amines enabled the dark equilibrium to be decoupled from closed‐isomer half‐lives for the first time.

## Graphical abstract

The use of 1,1,1,3,3,3‐hexafluoro‐2‐propanol (HFIP) leads to a streamlined synthetic access to donor–acceptor Stenhouse adduct (DASA) photoswitches through shortened reaction times and increased yields. This approach enables the diversification of DASA structures through the use of previously unreactive donor or acceptor groups, as well as simple access to DASA–polymer conjugates even when using electron‐poor amine donors.

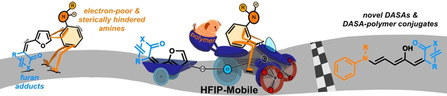

## Introduction

In recent years, photochromic molecules have found increased attention because of their ability to dynamically control physical and chemical properties with high spatial and temporal resolution.[^1^, ^2^] The incorporation of these photoresponsive molecules into materials has led to a range of developments from molecular machines to life‐science applications.[^3^, ^4^, ^5^, ^6^] Critical to advancing these applications has been the ability to optimize the photochromic properties, such as absorption profile, quantum yield, and the thermal stability of the metastable isomers through synthetic structural modification. Although clearly beneficial, optimization often also introduces more complicated synthetic strategies with longer synthetic sequences and lower yields. The widespread use of photochromic molecules requires easy accessibility, without compromising their tunability. Therefore, high‐yielding synthetic approaches using readily available starting materials for both small molecules and macromolecular systems remains an important goal.

**Figure 1:**
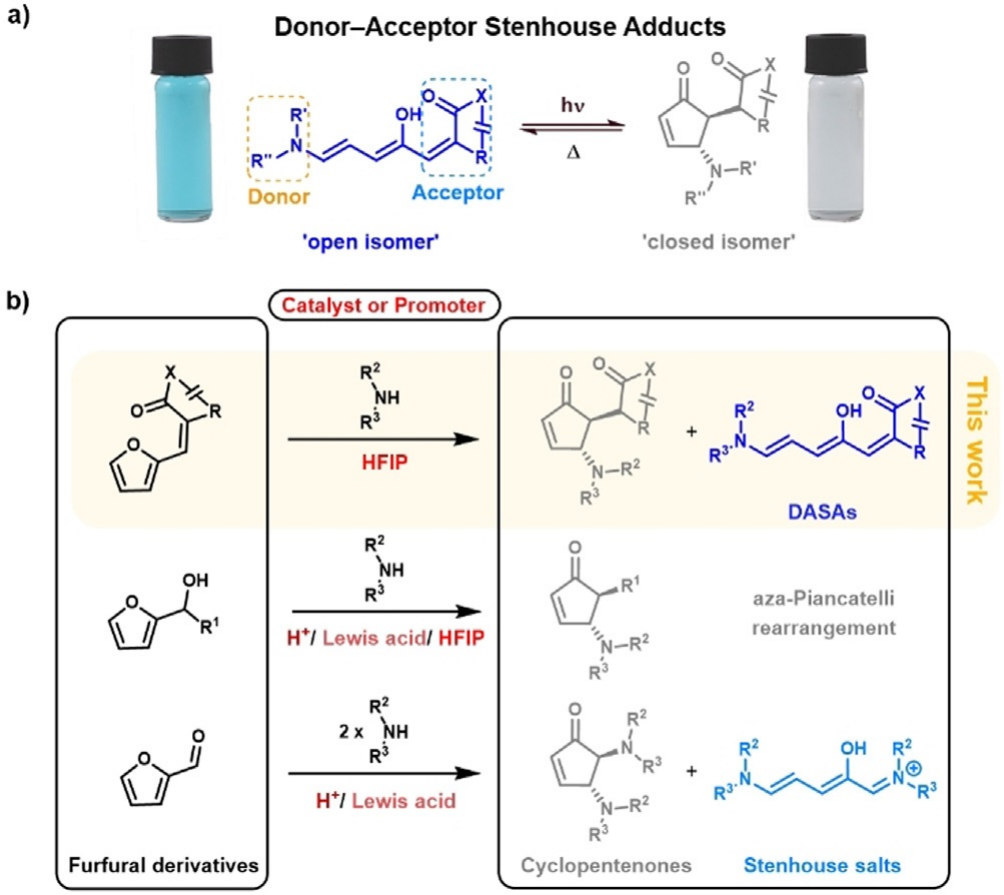
Donor–acceptor Stenhouse adducts (DASAs). a) General structures for open/colored triene and closed/colorless cyclopentenone isomers. R′/R′′: alkyl or aryl; X: O or N; R: electron‐withdrawing group, five‐ or six‐membered ring (a selection of donor and acceptor structures are displayed in Figure 2). b) General DASA synthesis in comparison to the closely related aza‐Piancatelli rearrangement and Stenhouse reaction.

Donor–acceptor Stenhouse adducts (DASAs) are a new class of visible‐light‐responsive photoswitches that were developed in 2014.[^7^, ^8^] DASAs exhibit a range of promising properties for photochromic materials, such as negative photochromism, visible‐light activation, and modular synthesis. Their architecture consists of a conjugated triene connecting an amine donor and a carbon acid acceptor, which upon irradiation can undergo a 4π‐electrocyclization to a closed cyclopentenone form (Figure 1 a).[^9^, ^10^] The “strength” of the electron‐donating or ‐withdrawing character of the donor and acceptor groups largely governs the overall switching properties, with structural modifications enabling these properties to be readily tuned. For example, replacing the dialkylamine donors from first generation derivatives (2014)[^7^, ^8^] with arylamines (second generation, 2016)[^11^, ^12^] provides access to DASAs with increased solvent compatibility, wavelength tunability, and tunable switching kinetics. The introduction of strong carbon acid acceptors (third generation, 2018)^[13]^ retained the advantageous properties of the second generation derivatives, while also providing better control over the thermodynamic equilibrium in the dark. In 2018, Beves and co‐workers also reported that minor steric modifications to the dialkylamine donor (first generation) dramatically improve the photoswitching properties of this class of DASAs.^[14]^ Similar to other classes of photoswitches, however, most of these modifications have come with increased difficulty in synthetic access.

DASA photochromes are derived from furfural, which serves as the precursor to the triene bridge, with the furan oxygen atom forming the hydroxy group in the final architecture. Using a straightforward, modular two‐step synthesis, furfural is attached to readily available carbon acid acceptor moieties through a Knoevenagel condensation followed by a ring‐opening reaction of the furan core by a secondary amine nucleophile, thereby resulting in the open‐form DASA photochrome (Figure 1 b, proposed full mechanism Figure S1). Despite the simplicity of this approach, some synthetic challenges remain to enable general access to this class of photochromes. Most importantly, the rate of the furan ring‐opening reaction strongly depends on the nucleophilicity of the amine donor and the electrophilicity of the acceptor group.[^12^, ^13^, ^15^, ^16^] By introducing arylamines as donors (second and third generation DASAs), reaction times can increase drastically—from minutes to multiple hours.[^12^, ^13^] The slow rate is particularly problematic when polymers are involved because of the concentration constraints and additional deceleration caused by steric effects from the polymer backbone. Here, reactions can take up to three weeks to reach full conversion, even with moderately reactive furan adducts derived from Meldrum's acid.[^17^, ^18^] Attempts to promote the ring‐opening reaction by using excess of the amine component leads to partial degradation of the DASA. Presumably, this results from nucleophilic attack on the triene and/or 1,4‐addition to the cyclopentenone closed form.[^19^, ^20^] Besides long reaction times, purification can also be a major challenge. Purification of DASA photochromes often relies on trituration or precipitation of the more hydrophobic open form, which takes advantage of the solubility differences between the open and closed form of DASAs and the corresponding degradation products. Common purification methods such as column chromatography are often low‐yielding, predominantly because of the conversion between the open and closed form that occurs during purification. This challenge is highlighted by the low yields (typically <50 %)[^11^, ^12^] observed for the synthesis of second generation DASAs. Decreasing the required reaction time, simplifying the purification of DASAs, and expanding the design space to enable the use of unreactive donors or acceptors would, therefore, further expand the utility of this new class of photochromes.

The intrinsic similarities between the DASA furan ring‐opening reaction and the Stenhouse reaction^[21]^ as well as aza‐Piancatelli rearrangement[^22^, ^23^] might provide the key to overcome some of the limitations currently associated with DASA synthesis (Figure 1 b). In 2007, Li and Batey rendered the condensation/ring‐opening/electrocyclization cascade reaction between furfural and two equivalents of an amine practical and synthetically useful by using lanthanide(III) catalysts.^[24]^ Subsequently, it was demonstrated by Read de Alaniz and co‐workers that rare‐earth Lewis acids such as dysprosium triflate (Dy(OTf)_3_) also serve as excellent catalysts for the rearrangement of furylcarbinols with a range of aniline nucleophiles.^[23]^ Since these initial reports, a number of Brønsted and Lewis acid catalysts have been shown to promote the ring‐opening reaction of furfural or 2‐furylcarbinols under mild conditions in the presence of a range of nucleophilic amines.[^23^, ^24^, ^25^, ^26^, ^27^, ^28^, ^29^] A challenge remained to identify conditions applicable to DASA synthesis that could simultaneously increase the rate of the furan ring‐opening reaction and inhibit the formation of the closed isomer during synthesis to streamline the purification process and synthetic access. Interestingly, Gandon and co‐workers reported several outstanding examples of the aza‐Piancatelli rearrangement, for which the Lewis acid catalyzed reaction proceeds in 1,1,1,3,3,3‐hexafluoro‐2‐propanol (HFIP) at room temperature in under an hour (selected substrates even reacted without additional catalyst when HFIP was used as solvent).^[26]^ HFIP has recently gained much attention for efficiently promoting a wide range of organic reactions catalyzed by Lewis and Brønsted acids.[^30^, ^31^]

Herein, we report the use of HFIP as a mild Lewis/Brønsted acid in the synthesis DASA to promote the ring‐opening reaction of furan adducts for facile access to a broad range of DASA photoswitches, including a range of new DASA derivatives bearing deactivated amine donors that were previously unreactive. Furthermore, we show that HFIP shifts the DASA equilibrium to the open form through hydrogen‐bonding interactions, thereby simplifying work‐up and purification procedures and increasing the overall yields of the isolated products. This method is also applied to prepare DASA–polymer conjugates, with the required time for functionalization reduced from days to several hours and allowing the preparation of more structurally diverse DASA materials.

## Results and Discussion

### HFIP as Promoter in DASA Synthesis

**Figure 2:**
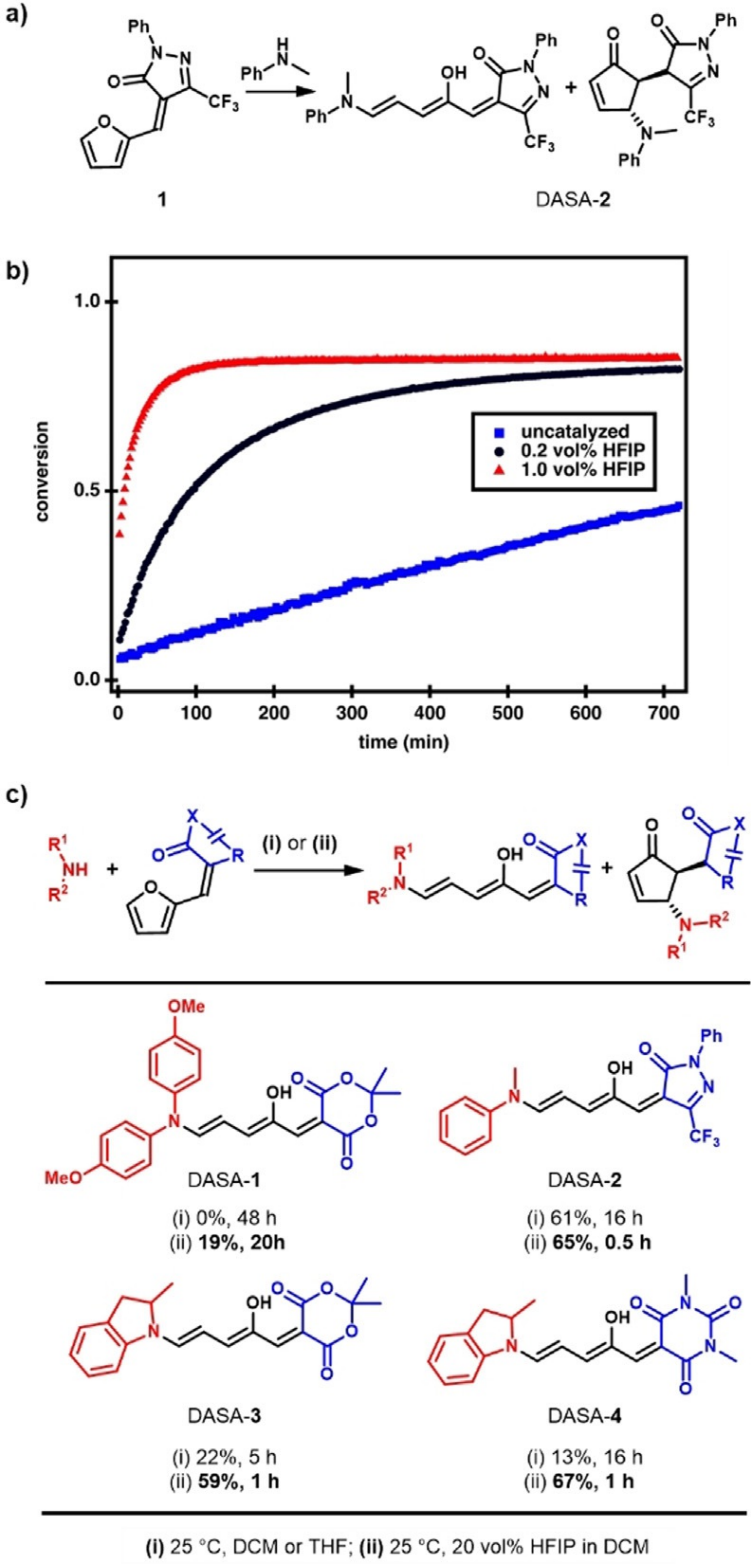
Effect of HFIP on the reaction rate and yields in DASA synthesis. a) Reaction scheme of the ring opening of CF_3_‐pyrazolone‐derived furan adduct **1** and *N*‐methylaniline. b) Conversion plots from in situ ^1^H NMR experiments of the reaction displayed in (a) on applying different amounts of HFIP in deuterated dichloromethane at 25 °C. The second‐order rate constant increases from 3±1 m ^−1^ h^−1^ (0 vol %) to 11.6±0.2 m ^−1^ h^−1^ (0.2 vol %, 1 equivalent relative to **1**) to 56±5 m ^−1^ h^−1^ (1 vol %, 5 equivalents relative to **1**). c) Comparison of yields of isolated product and reaction time in the synthesis of a series of DASAs under traditionally used reaction conditions and by application of 20 vol % HFIP in dichloromethane. Yields obtained under uncatalyzed conditions for DASA‐**3** and DASA‐**4** were taken from the literature.^[13]^

Initial attempts to promote the ring‐opening reaction commenced with the use of Lewis acid catalysts such as dysprosium triflate (Dy(OTf)_3_), a commonly used catalyst in the aza‐Piancatelli reaction.[^23^, ^27^] Unfortunately, all our attempts to use metal‐based Lewis acids in DASA synthesis resulted in degradation of the product or starting materials. Inspired by the work of Gandon and co‐workers and their successful application of HFIP in the aza‐Pinacatelli rearrangement,[^26^, ^31^] we next explored the use of HFIP as a mild Lewis/Brønsted acid in DASA synthesis. HFIP has a high polarity and ionization potential as well as moderate acidity (p*K*_a_=9.3) in combination with low nucleophilicity and a strong hydrogen bond donating ability, which could possibly provide a way of assisting in the electrophilic activation of the furan adduct and stabilizing charged intermediates.[^30^, ^31^] Furthermore, as a consequence of its low boiling point (b.p.: 59 °C)^[30]^ HFIP can be easily separated from the reaction mixture by evaporation in vacuo before purification. Initial experiments were conducted on the effect of HFIP as a cosolvent with dichloromethane for the reaction of 4,4′‐dimethoxydiphenylamine with the furan adduct derived from Meldrum's acid. 4,4′‐Dimethoxydiphenylamine is unreactive under the standard reported conditions for DASA formation with this acceptor.^[13]^ In contrast, rapid color formation was observed by the naked eye, thus supporting the formation of DASA‐**1** and the promising ability of HFIP to promote the synthesis of DASAs. ^1^H NMR and UV/Vis kinetic studies were then used to confirm this qualitative evaluation. For this we utilized the third generation CF_3_‐pyrazolone‐derived furan adduct **1** and *N*‐methylaniline (Figure 2 a) as readily available starting materials that provided good signal separation in the ^1^H NMR spectrum. The reaction progress in the synthesis of DASA‐**2** was monitored by continuous in situ analysis in deuterated dichloromethane in the presence and absence of HFIP. Dichloromethane is a suitable cosolvent for HFIP as it is inert under acidic conditions and does not form hydrogen‐bonded complexes with HFIP. As shown in Figure 2 b, a drastic rate increase was observed using only 0.2 and 1 vol % of HFIP (corresponding to 1 and 5 equivalents relative to **1**), with second‐order rate constants (*k*) increasing from 3±1 m ^−1^ h^−1^ to 11.6±0.2 and 56±5 m ^−1^ h^−1^, respectively (Table S1). The accelerating effect was strongly enhanced at higher concentrations (Figure S2) and, importantly, no signs of the formation of side products or degradation were detectable by ^1^H NMR spectroscopy, even when going up to 20 vol % HFIP, while also improving the yields of the isolated products (Figure 2 c). Utilizing higher amounts of HFIP accelerated the reaction further, however, degradation of the product and starting material could be observed at concentrations >50 vol % (Figure S2). In this study, a maximum of 20 vol % HFIP (1 to 10 equivalents relative to the furan adduct) was, therefore utilized, for all syntheses. Reactions can be performed in the open air. Importantly, removing HFIP by evaporation in vacuo enables purification by trituration, similar to previously reported procedures.[^7^, ^12^]

Encouraged by this initial result, we next explored the generality of this method toward the synthesis of various other first, second, and third generation DASAs. The synthesis of first generation DASAs bearing strongly basic alkyl amines (as compared to anilines used for second and third generation DASAs) were inhibited by the addition of HFIP (Figure S3). Presumably, this is due to the basicity of the secondary alkylamines, which are known to form stable hydrogen‐bonding complexes with HFIP, which leads to an adverse effect on the reaction rate.[^32^, ^33^, ^34^] In contrast, the synthesis of both second and third generation DASAs greatly benefited from the addition of HFIP (Figure 2 c). For example, a fivefold increase in yield was achieved for the synthesis of DASA‐**4** within a fraction of the usual reaction time: 67 % in 1 h vs. 13 % in 16 h. This demonstrates that the use of low concentrations of HFIP with a cosolvent improves access to DASA‐based photoswitches in terms of yield and reaction time, while also expanding the accessible DASA design space.

To better understand the role of HFIP in mediating the ring‐opening reaction of the furan adduct, we monitored the rate of DASA‐**2** formation with other polar solvents and alcohols by ^1^H NMR spectroscopy and investigated the electronic ground‐state properties of the furan adduct by UV/Vis spectroscopy in various solvents. Analogous NMR kinetic experiments were conducted with 2‐propanol instead of HFIP (Figures S4–S9). As previously observed for polar protic solvents,[^13^, ^16^] we found an accelerating effect for 2‐propanol (*k*: 9 m ^−1^ h^−1^ for 2 vol %), but it was substantially smaller than that of its fluorinated analogue (*k*: 56 m ^−1^ h^−1^ for 1 vol %). More interestingly, using the methyl ether of HFIP (HFIPMe) instead of HFIP had only minor effects on the reaction rate (*k*: 5 m ^−1^ h^−1^ for 3 vol %). HFIPMe is comparably polar to HFIP (relative dielectric constant: *ϵ*_HFIP_=17.8,^[35]^ *ϵ*_HFIPMe_=15.4^[36]^), exhibits similar hydrogen bond acceptor properties, but lacks the hydrogen bond donor ability that is suspected to be responsible for the promotion of activity seen in many other organic transformations.^[37]^ UV/Vis spectroscopic analysis of the CF_3_‐pyrazolone‐derived furan adduct **1** revealed that the spectral properties are largely insensitive to polarity differences of a variety of protic and aprotic solvents but are strongly altered in HFIP (Figures S10 and S11). These results suggest that the hydrogen bonding of HFIP with the furan adduct plays a role in the observed rate increase, potentially by increasing their electrophilic character. However, the specific mechanism at play here requires further study, as it is likely that multiple effects (polarity, hydrogen bond donor ability, possibly acidity) contribute to the observed promotion of the reaction. Moreover, other effects such as off‐cycle binding of the amine nucleophile with HFIP (of relevance for strongly basic alkyl amines, Figure S3) and product inhibition (see below) further complicate the full mechanistic picture.

### HFIP as Modulator of Thermodynamic Equilibrium and Photoswitching

**Figure 3:**
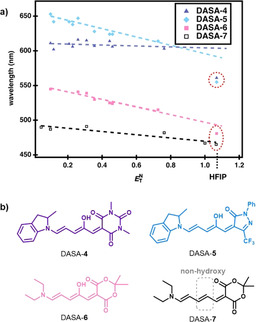
DASA solvatochromic shift analysis. a) Solvatochromic shifts using the Dimroth–Reichardt *E* ^N^_T_ solvent polarity scale for first to third generation DASAs and a non‐hydroxy analogue in solvents of different polarity. A deviation from nonlinearity is observed only for DASAs in HFIP, which indicates the presence of hydrogen bonding interactions with the triene hydroxy group. b) Chemical structures of the DASAs in (a).

**Figure 4:**
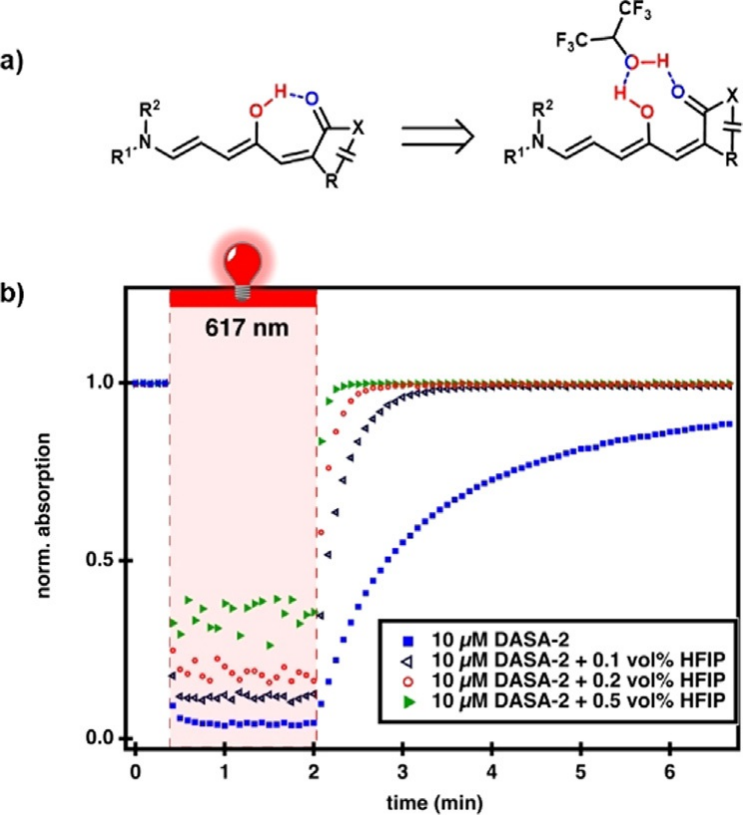
Hydrogen bonding interaction of HFIP and the DASA open form and the effect of photoswitching. a) General scheme for the proposed hydrogen bonding in DASAs in the presence and absence of HFIP. b) The use of time‐dependent UV/Vis spectroscopy to observe the photochromic behavior of DASA‐**2** (10 μm) in toluene and toluene/HFIP followed at *λ*_max_ (625 nm).

We then investigated the impact of HFIP on the thermodynamic equilibrium between the open and closed form of DASAs. Previous studies showed that protic solvents (e.g. methanol) and polar solvent (e.g. acetonitrile) afford a mixture of open and closed isomers, which renders the purification more difficult and, in general, results in lower yields of isolated products (often only the more hydrophobic open form can be isolated in high yield by trituration).[^8^, ^38^] ^1^H NMR spectroscopic analysis revealed that in the presence of HFIP, minimal formation of the closed form occurred, despite the polarity and acidity of HFIP (Figures S12–S16). Analogous experiments in a polar solvent such as acetonitrile (*ϵ*=36.6)^[39]^ led to mostly the closed form (Figure S17). These results support that HFIP is unique amongst the protic polar solvents for its ability to stabilize the open form of DASA photochromes. To further investigate the effects of solvent on the open form, we analyzed solvatochromic shifts^[40]^ of first, second, and third generation DASAs (DASA‐**4**–DASA‐**6**) as well as a non‐hydroxy DASA analogue in solvents of different polarity (Figure 3 and Figures S18–S21). HFIP showed a clear deviation from the linear trend in terms of the solvatochromic shifts observed with the other solvents, thus indicating the presence of additional specific interactions in HFIP. Of note, the non‐hydroxy DASA analogue (DASA**‐7**) showed a linear trend in its solvatochromic shift in all solvents, including HFIP, which shows that the hydroxy group is likely responsible for the nonlinear behavior of regular DASA compounds. A detailed study on the interpretation of solvatochromic shifts in terms of delocalization of the electronic ground‐state structure for different DASA generations was recently published.^[41]^ To also evaluate the effects of HFIP on the switching properties, DASA‐**2** was placed in a solution of dichloromethane with 0.5 vol % HFIP and irradiated with light of *λ*=530 nm (Figure S22). In the presence of HFIP, DASA‐**2** undergoes only an 11 % decrease in absorption upon irradiation, followed by rapid recovery to the open form in the dark. In contrast, in pure dichloromethane, a substantially increased photothermal stationary state (PTSS, equilibrium between the light‐driven forward reaction and the purely thermal back reaction;^[42]^ 81 % closed isomers) and a noticeably slower recovery rate were observed. These results highlight that the thermodynamic equilibrium and PTSS clearly shift to the open form in the presence of HFIP, even at low concentrations. We propose that a hydrogen bond donor interaction between the hydroxy group on the triene backbone and HFIP must be critical for stabilizing the open form and controlling the thermodynamic equilibrium and PTSS. A possible hydrogen bond donor interaction is shown in Figure 4 a.^[43]^

Building on this hypothesis, we speculated that a modulation of the intramolecular hydrogen bond strength in the open form could also be exploited to systematically shape the overall switching kinetics of DASA compounds.[^43^, ^44^] To test this idea, we explored the role of HFIP in toluene, in which an excellent PTSS performance (>95 % closed isomers at PTSS for most DASA derivatives) and a slower thermal recovery than in chlorinated solvents is observed.

As highlighted in Figure 4 b, the closed‐form half‐life of DASA‐**2** is reduced from 94 s to 2.5 s upon the addition of only 0.1–0.5 vol % HFIP, and reaches a lower value than the corresponding substantially more electron‐rich 2‐methylindoline derivative (DASA‐**5**, 40 s in toluene).^[13]^ This demonstrates a facile pathway to externally modulate DASA switching kinetics through addition of a simple hydrogen bond donor, thus opening the door to systems relying on finely controlled switching kinetics.

### New DASA Derivatives

**Figure 5:**
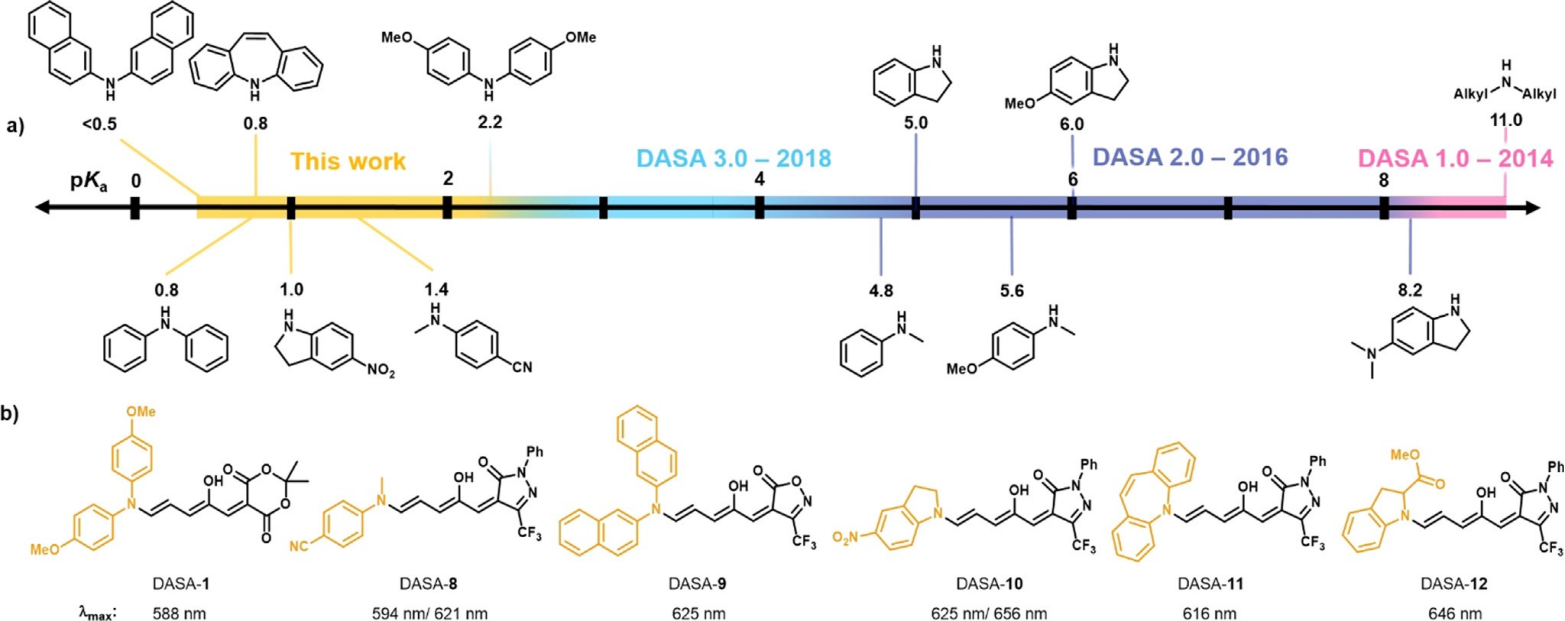
New DASAs bearing weak amine donors that were synthesized using HFIP. a) Different secondary amine donors examined previously and herein for DASA synthesis ordered according to the p*K*_a_ values of their corresponding acid (calculated with SciFinder^®^), which correlate well with the reactivity in the furan ring‐opening reaction. b) Chemical structures of the new DASAs and their *λ*_max_ in chloroform. Note: some of the DASAs display split absorption bands (Figure S23–S36).

To further illustrate the advantage of this new method, we sought to expand the scope of DASAs by including highly unreactive amine donors to prepare previously unattainable DASA derivatives (Figure 5 a). Previously reported DASAs were limited to considerably nucleophilic amine donors with p*K*_a_ values of their conjugated acid above about 4.8. One exception is 4,4′‐dimethoxydiphenylamine (p*K*_a_ conjugated acid of ca. 2.2) that only reacted with a highly electron poor and unusually reactive CF_3_‐isoxazolone‐derived furan adduct.^[13]^ Herein, we present a number of DASAs bearing extremely non‐basic amine donor moieties whose conjugate acids have p*K*_a_ values approaching 0.5 (Figure 5 a). Driven by the increased light penetration depth and biological compatibility of near‐IR light, we initially focused on arylamine derivatives with the potential ability to red‐shift the *λ*_max_ through hyperconjugation. Using 20 vol% HFIP in dichloromethane provided access to a range of new DASA derivatives for the first time, including derivatives bearing aniline with highly deactivating groups, sterically hindered aromatic amines, and new acyclic and cyclic aromatic amines (Figure 5 b). To compare the properties of the DASAs synthesized from these new donors, the CF_3_‐pyrazolone‐derived furan adduct **1** was primarily used as the acceptor compound. For the weakest donor, 2,2′‐dinaphthylamine, a more reactive CF_3_‐isoxazolone‐derived acceptor (**S4**) was used to yield DASA‐**9**. These furan adducts were synthesized by Knoevenagel condensation of the respective carbon acids with furfural in a first step according to previously reported procedures.[^8^, ^13^] The furan ring‐opening reactions of the furan adducts in 20 vol % HFIP can be conducted under ambient conditions, open to air, and after completion, evaporation of the solvents in vacuo enables isolation of the desired compounds as solids that can be filtered and purified by trituration with diethyl ether. For example, **1** reacted in 16 h with iminostilbene to afford DASA‐**11**, which was isolated in good yield (65 %) after trituration with diethyl ether. Full characterization data of all the compounds can be found in the Supporting Information.

### Absorption Properties of New DASAs

**Figure 6:**
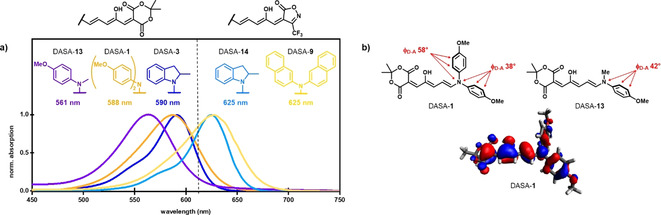
UV/Vis absorption properties of new DASAs. a) Absorption spectra (chloroform) of DASA‐**1** and DASA‐**9** compared to previously reported DASA derivatives.[^12^, ^13^] b) Computational density functional theory modeling of DASA‐**1** to determine the HOMO orbital overlap and dihedral angles between the donor and acceptor (Φ_D‐A_) in comparison to the respective second generation DASA^[12]^ bearing only one phenyl substituent.

With access to a range of new DASA scaffolds, we explored their absorption profiles by UV/Vis spectroscopy (Figure 6 a and Figures S23–S36). In general, these DASA derivatives have similar properties to the previously reported second and third generation DASAs, despite notable structural differences.[^7^, ^12^, ^13^] For example, the two 4‐methoxyphenyl substituents in DASA‐**1** resulted in the same maximum absorption wavelength of about 590 nm in chloroform as the respective unsubstituted indoline derivative that formally provides only one phenyl ring (DASA‐**3**).[^12^, ^13^] Presumably, this is due to the substantial out‐of‐plane twist of both aryl substituents on the acyclic amine donors and, therefore, reduced HOMO overlap/conjugation. This phenomenon was extensively investigated before to explain the bathochromic shift of about 30 nm observed for cyclic indoline relative to the corresponding *N*‐methylaniline derivatives.^[12]^ In analogy to the previous study,^[12]^ density functional theory (DFT) calculations at the B3LYP‐GD3BJ/6‐31G(d) level of theory were used for geometry optimizations of the open‐form DASAs to compare dihedral angles between the acceptor and donor groups in this new series (Figure 6 b, Table S2, Figures S41 and S42). In agreement with the literature,^[12]^ a dihedral angle of Φ_D‐A_≈40° between the triene acceptor system and the aryl donor group on the opposite side of the hydroxy group was found for the acyclic amine donors, whereas the indoline‐derivative (DASA‐**5**) is completely planar. The second aryl group in DASA‐**1** and DASA‐**9** was predicted to be even more twisted (Φ_D‐A_≈60°) and, therefore, is expected to contribute even less to the homoconjugation and the bathochromic absorption shift (Figure 6 b). The iminostilbene moiety in DASA‐**11** adopts a boat‐like conformation similar to the experimentally determined solid‐state structures^[45]^ of iminostilbene derivatives, with both phenyl rings substantially out‐of‐plane (Φ_D‐A_≈60°), which explains the hypsochromic shift observed relative to the corresponding *N*‐methylaniline derivative (DASA‐**2**). A 11 nm red‐shift can be achieved with commercially available and stable *p*‐nitroindoline in DASA‐**10** (*λ*_max_=656 nm, Figures S31 and S32) relative to the respective 2‐methylindoline derivative (DASA‐**5**).^[13]^ Since the planarity of the aryl groups of the donors are critical for increasing the conjugation and extending the absorption maximum wavelength, we attempted to utilize other cyclic amines, including acridone and carbazole derivatives. However, it was found that these amines, whose conjugated acids have p*K*_a_ values <0, are either unreactive under the optimized reaction conditions or result in unstable adducts that prevented product characterization.

### Photoswitching of New DASAs

Expanding the scope towards more electron‐deficient amines allowed us to compare the effect of these weakly donating groups on the photoswitching behavior with the arylamines introduced previously.[^12^, ^13^] For this, time‐dependent UV/Vis spectroscopic analysis of the overall switching kinetics and ^1^H NMR spectroscopic analysis of the dark state were carried out (Figures S43–S56, S62–S73).

Detailed results are tabulated in the Supporting Information (Table S3). Similar to the introduction of arylamines instead of alkylamines for barbituric acid and Meldrum's acid acceptors reported in 2016,^[12]^ the use of even more electron‐deficient amines for pyrazolone and isoxazolone derivatives increased the half‐life of their closed form while maintaining the red‐shifted *λ*_max_ wavelength that these acceptor groups enable (e.g. DASA‐**5**: 5 s, DASA‐**2**: 13 s. DASA‐**11**: 202 s). Another similarity is the shift of the thermodynamic equilibrium towards the closed isomer with more weakly donating amines, as observed for DASA‐**8** and DASA‐**10** (both <5 % open at equilibrium in chloroform), which is in accordance with previous studies.^[11]^ This electronic effect on the equilibrium position can be compensated by introducing sterically hindered groups, as was shown in 2018,^[14]^ which is in line with what we observed when utilizing amines with bulky secondary phenyl moieties in DASA‐**1**, DASA‐**9**, and DASA‐**11** (32 %, >95 %, and 89 % open at equilibrium in chloroform). This allows the formation of DASAs with a large amount of open form in the dark while exhibiting long‐lived closed‐form isomers after photoswitching, which could previously not be decoupled.[^11^, ^12^, ^13^]

**Figure 7:**
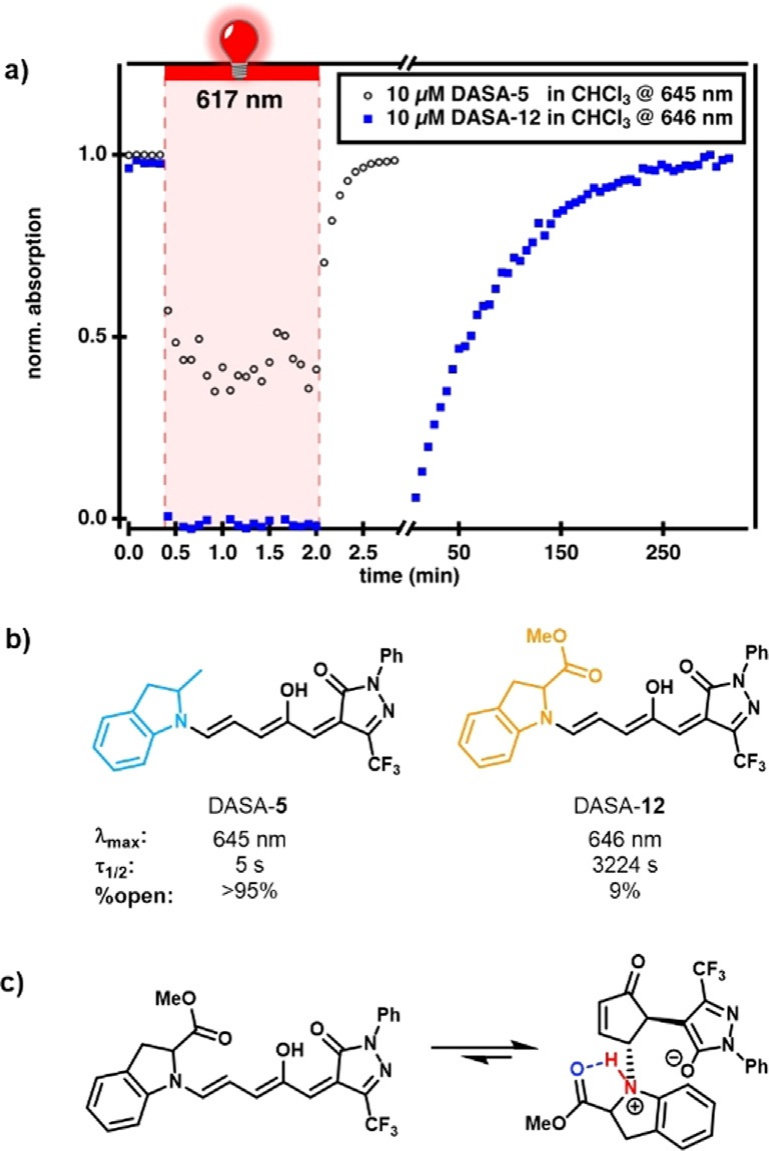
Photochromic properties of DASA‐**12** compared to DASA‐**5**. a) The use of time‐dependent UV/Vis spectroscopy to observe the photochromism of DASA‐**5** and DASA‐**12** in chloroform at 10 μm (initial absorbance: 0.9 and 0.1) followed at *λ*_max_ (645 nm and 646 nm). Quantitative conversion of the open form to the closed form under irradiation with light at 617 nm for 100 s and subsequent thermal recovery in the dark can be observed. To ensure comparability, this experiment was also performed at similar initial absorbances (100 μm DASA‐**12**, Figure S61). b) Comparison of the photochromic parameters of DASA‐**5** and DASA‐**12** in chloroform.^[13]^ c) Presumed hydrogen bonding stabilizing the zwitterionic closed form of DASA‐**12**.

One interesting exception to this trend is DASA‐**12**, which includes a sterically bulky methyl ester in the 2‐position of an indoline donor. Against expectation, the thermodynamic equilibrium is massively shifted towards the closed form when compared to the previously published 2‐methylindoline derivative (DASA‐**5**), while the half‐life of the closed form after irradiation is greatly increased (>3200 s vs. 5 s, Figures 7 a,b). As a result of the increased stability of the closed form of DASA‐**12**, it could be shown by 2D NMR spectroscopy (Figures S75 and S76) to be zwitterionic in chloroform. This is the first report on the nature of the closed form of a DASA with a third generation acceptor and aryl donor, and demonstrates a clear difference to the second generation Meldrum's and barbituric acid derivatives that reside mostly in the keto isomer closed form.[^11^, ^12^, ^44^] Presumably, the increase in the half‐life of the closed isomer of DASA‐**12** is partially due to a hydrogen bond stabilization of the protonated amine in the zwitterionic form by the carbonyl ester moiety through formation of a five‐membered ring (Figure 7 c). This shows an interesting design principle that enables the intramolecular stabilization of a selected form of the closed DASA isomer and its ability to influence overall switching kinetics.^[46]^

### Fluorescence of DASA‐9

Beyond controlling the half‐life, new properties can be introduced by the utilization of functional amines. Subjecting fluorescent 2,2′‐dinaphthylamine to furan adduct **S4** in the presence of 20 vol % HFIP in dichloromethane afforded DASA‐**9**. Interestingly, it was found that DASA‐**9** shows a fluorescence emission with a maximum at about 400 nm upon excitation into the absorption bands that are characteristic of the naphthyl groups (300–350 nm, Figures S77 and S78). The shape and position of the fluorescence emission band did not change, and the intensity of the emission was found to change only slightly when the DASA was converted into the closed form (Figure S78). This is in agreement with DFT calculations that predicted a limited electronic coupling between the naphthyl groups and the triene acceptor system in the open form (Table S2). However, we cannot completely exclude that trace amounts of highly fluorescent, free 2,2′‐dinaphthylamine is present, which could complicate the fluorescence measurements. DASAs in general are weakly fluorescent when excited at their maximum absorbance wavelength (π‐π* transition of open form), so that prior examples of strongly fluorescent DASAs have been limited to systems that are based on the attachment of separate fluorophores.[^47^, ^48^, ^49^, ^50^] Introducing easily accessible fluorescent donors could thus enable further development of multifunctional materials and easily identifiable DASA‐functionalized materials.

### Polymer Conjugation

**Figure 8:**
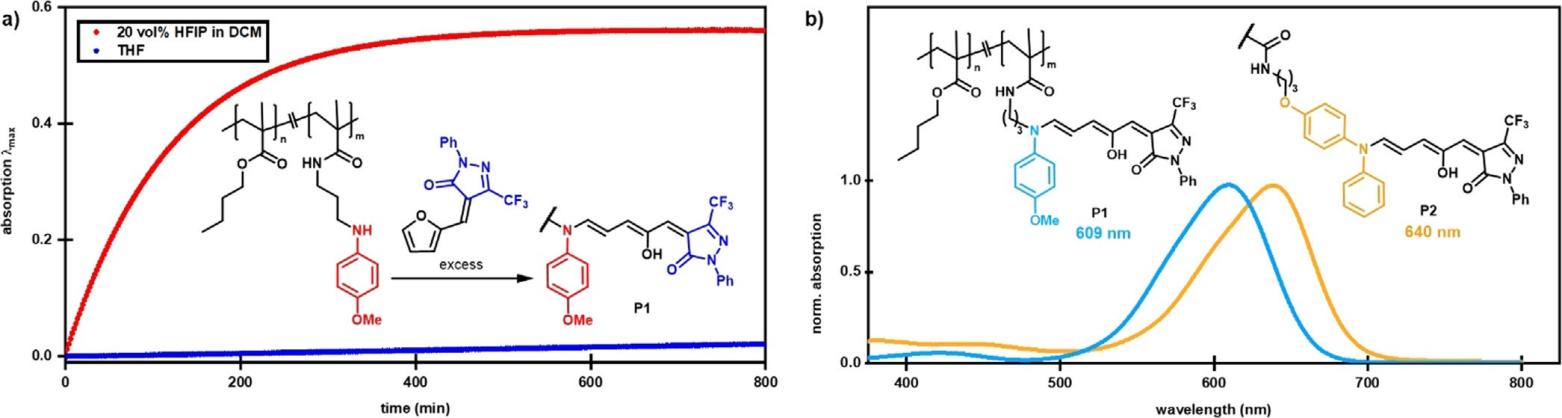
HFIP‐promoted synthesis of DASA–polymer conjugates and properties of the resulting polymers. a) In situ absorbance monitoring at *λ*_max_ (open form DASA) of the furan ring‐opening reaction to afford **P1** in THF or using HFIP in dichloromethane. b) Absorption spectra (chloroform) and chemical structures of DASA‐functionalized poly(butyl methacrylate) polymers **P1** and **P2** (*m*: 4 mol %).

The utility of HFIP to promote the formation of DASAs was also explored beyond small molecules by investigating its applicability to the synthesis of DASA–polymer conjugates. Previous syntheses of DASA–polymer conjugates relied on first installing secondary amine precursors onto the polymer and then a subsequent reaction with an excess of furan adduct.[^17^, ^18^, ^51^, ^52^] Although this approach provided access to DASA–polymer conjugates, it suffered from slow reaction and favored the use of electron‐rich amine donors. As such, we were pleased to discover that HFIP improved access to DASA–polymer conjugates, reducing the reaction time from days to hours (Figure 8 a). For example, treatment of an *N*‐methylmethoxyaniline‐functionalized simple model poly(butyl methacrylate) with an excess of the CF_3_‐pyrazolone‐derived furan adduct in the presence of 20 vol % HFIP in dichloromethane for 5 h yielded the new DASA polymer **P1**. To further demonstrate the power and generality of this HFIP‐accelerated postfunctionalization approach, we sought to broaden the process to diphenylamine as a weak amine donor, which is largely unreactive under previously reported reaction conditions. The polymer bearing diphenylamine groups was synthesized by aminolysis of the activated ester groups.^[17]^ Treatment of this material with an excess of the CF_3_‐pyrazolone–furan adduct in the presence of 20 vol % HFIP in dichloromethane overnight and subsequent purification by size‐exclusion chromatography gave DASA–polymer conjugate **P2** (characterization data for the polymers can be found in the Supporting Information). Analysis by UV/Vis spectroscopy confirmed the formation of the new DASA adduct (Figure 8 b). The DASA–polymer conjugates can be converted into the closed form upon irradiation (PTSS of 40 % closed form for **P1** and 85 % for **P2** in toluene) and fully recover their initial absorbance in the dark (Figures S57–S60). These examples highlight that the new reaction conditions improve the reaction rates and provide access to new DASA‐based functional materials with greater tunability of the absorption wavelength and photoswitching response.

## Conclusion

In conclusion, we have developed a practical solution for accelerating the furan ring‐opening reaction in the synthesis of DASAs by using HFIP. Importantly, the new method can be performed at ambient temperature and HFIP can be readily removed from the reaction mixture by simple evaporation. We demonstrate that the addition of HFIP greatly shortens the reaction time and improves the yields of new, as well as previously reported adducts, including DASA–polymer conjugates. Furthermore, the method offers access to a broader range of DASA photoswitches by enabling the use of electron‐deficient aromatic amines and new furan adducts. The introduction of sterically hindered, electron‐poor donors allows the design of DASA derivatives with long closed‐form half‐lives after photoswitching, while maintaining large amounts of the open isomer in the dark. Moreover, the use of HFIP allows more facile and faster access to DASA–polymer conjugates, thus lowering the barrier of entry into the growing field of functional DASA materials. The use of HFIP as an external modulator of DASA photoswitching kinetics will further enable the design of systems with tailored responses for selected applications.

## Conflict of interest

The authors declare no conflict of interest.

## Supporting information

As a service to our authors and readers, this journal provides supporting information supplied by the authors. Such materials are peer reviewed and may be re‐organized for online delivery, but are not copy‐edited or typeset. Technical support issues arising from supporting information (other than missing files) should be addressed to the authors.

Supplementary
