# Supplementary material for: Promoting the Furan Ring‐Opening Reaction to Access New Donor–Acceptor Stenhouse Adducts with Hexafluoroisopropanol
Source: Angew Chem Int Ed Engl. 2021 Mar 18;60(18):10219–27. doi: 10.1002/anie.202100115 (PMC8068666; doi:10.1002/anie.202100115)
Supplement: Supplementary file 1 — Supplementary [file ANIE-60-10219-s001.pdf]

Angewandte Chemie
Eine Zeitschrift der Gesellschaft Deutscher Chemiker

Supporting Information

Promoting the Furan Ring-Opening Reaction to Access New Donor–Acceptor Stenhouse Adducts with Hexafluoroisopropanol

Michèle Clerc[+], Friedrich Stricker[+], Sebastian Ulrich, Miranda Sroda, Nico Bruns, Luciano F. Boesel,* and Javier Read de Alaniz**

anie_202100115_sm_miscellaneous_information.pdf

Table of Contents

1. General Experimental 3
1.1 Chemicals 3
1.2 Instruments and Methods 3
2. Synthesis 4
2.1 Proposed Mechanism 4
2.2 Furan Adducts 4
2.3 Amines 6
2.3 DASAs 7
2.4 Polymers 11
3. Kinetics Experiments 12
3.1 UV-Vis Spectroscopy 12
3.1.1 Rates as Function of HFIP Concentration 12
3.1.2 Alkyl vs Aryl Amines 13
3.1.3 Polymers 14
3.2 NMR Spectroscopy 14
3.2.1 Kinetic Fitting 15
4. UV-Vis Absorption Spectra of Furan Adducts 18
5. Effects on Thermodynamic Equilibrium 18
5.1 NMR Spectroscopy 18
5.2 Solvatochromic Shift Analysis 22
5.3 Effect of HFIP on PTSS 23
6. UV-Vis Absorption Spectra 23
7. DFT Calculations 29
8. Photoswitching Experiments 32
8.1 Overview Switching Parameters 33
8.2 Time Dependent UV-Vis Absorption Spectra 35
8.3 Thermodynamic Equilibrium NMR Spectroscopy 41
8.4 2D-NMR Spectroscopy 47
9. Fluorescence 49
10. Probing the H-bonding in the Presence of HFIP 53
11. Spectra 54
11.1 NMR Spectra 54
11.2 IR Spectra 67
12. References 67

1. General Experimental

1.1 Chemicals

All commercially obtained reagents were bought from Sigma Aldrich, TCI Europe or Fisher Scientific and were used without purification, except furfural, which was distilled prior to usage. Size exclusion beads (Bio-Beads S-X1 Support, 0.6–14 kDa) were obtained from Bio-Rad Laboratories. Anhydrous solvents were either obtained from Sigma Aldrich or from a solvent purification system.

1.2 Instruments and Methods

Room temperature reactions were carried out between 22–25 ºC. Thin layer chromatography (TLC) was performed using Merck TLC plates (silica gel 60 F254 on aluminum) and visualized by UV light (254/ 366 nm) or staining with $KMnO_4$/NaOH. Silica gel chromatography was performed using silica gel from Sigma Aldrich (technical grade, 60 Å pore size, 40–63 µm particle size). Size exclusion chromatography (SEC) was performed on Bio-Beads S-X1 Support beads using distilled THF as mobile phase. ^{1}H and ^{13}C nuclear magnetic resonance (NMR) spectra were measured at 298 K on a Bruker Avance III 400 (400 MHz) NMR spectrometer, a Varian Unity Inova 500 MHz, or a Varian Unity Inova AS600 600 MHz spectrometer. ^{19}F NMR spectra were recorded on a Bruker Avance III 400 (400 MHz) NMR spectrometer. Chemical shifts (δ) are reported in ppm and referenced internally from the proteo-solvent resonance. Coupling constants (J) are reported in Hz. Abbreviations for the peak multiplicities are *s* (singlet), *d* (doublet), *dd* (doublet of doublet), *t* (triplet), *q* (quadruplet) and *m* (multiplet). For diffusion-edited ^{1}H NMR spectra, 40% gradient strengths were applied to selectively suppress the signals of low molecular weight species. Gas chromatography/electron impact ionization-mass spectrometry (GC/EI-MS) was measured on a Thermo Scientific ISQ GC/MS equipped with an ISQ 7000 and Trace 1300 GC using a Thermo Scientific TG-SQC capillary column (15 m, 0.25 mm I.D., 0.25 m thickness). Split/splitless injector at 280 °C; flowrate at 1 mL min^{-1}; gradient set to 20 °C min^{-1} from 30 °C to 300 °C, then isothermal for another 4 min. EI set to 70 eV; single stage quadruple mass analyzer; mass range 35–600 amu at 2 scans min^{-1} in full scan mode. The retention time (R_t) is reported in min, the mass of molecular ions and characteristic fragments with >15 rel.% are reported as *m/z* (rel.%). High resolution mass spectrometry (HR-MS) was measured on a Waters LCT Premier ESI TOF. Attenuated total reflection Fourier-transform infrared (ATR FT-IR) spectra were recorded on a Varian 640-IR FT-IR spectrometer equipped with an ATR (attenuated total reflection) accessory or a Thermo Nicolet iS10 FTIR Spectrometer with a Smart Diamond ATR; applied as neat samples and absorbance bands reported as 1/λ in cm^{-1}. Abbreviations for the relative band intensities are *s* (strong), *m* (medium), *w* (weak). Gel permeation chromatography (GPC) was measured on an Agilent 1100 Series high-performance liquid chromatography (HPLC) system on serial coupled PSS SDV 5 m 100 Å and PSS SDV 5 m 1000 Å columns maintained at 30 °C (allows separation from ca. 1–1000 kDa). Signals were recorded on a diode array detector (235 nm/ 360 nm) and a refractive index (RI) detector (at 35 °C). Measurements were performed in THF as an eluent relative to narrow molecular weight PS standards. UV-Vis absorbance spectra were recorded on an Agilent Cary 4000 UV-Visible spectrophotometer or an Agilent 8453 UV-Visible Spectrophotometer G1103A. Details for UV-Vis absorption and photoswitching measurements are presented in the respective *section 8*. Photoluminescence spectra were measured on a Horiba Scientific Fluoromax-Plus fluorescence spectrometer at room temperature (excitation and emission slit widths were set to 2 nm unless otherwise stated).

2. Synthesis

2.1 Proposed Mechanism

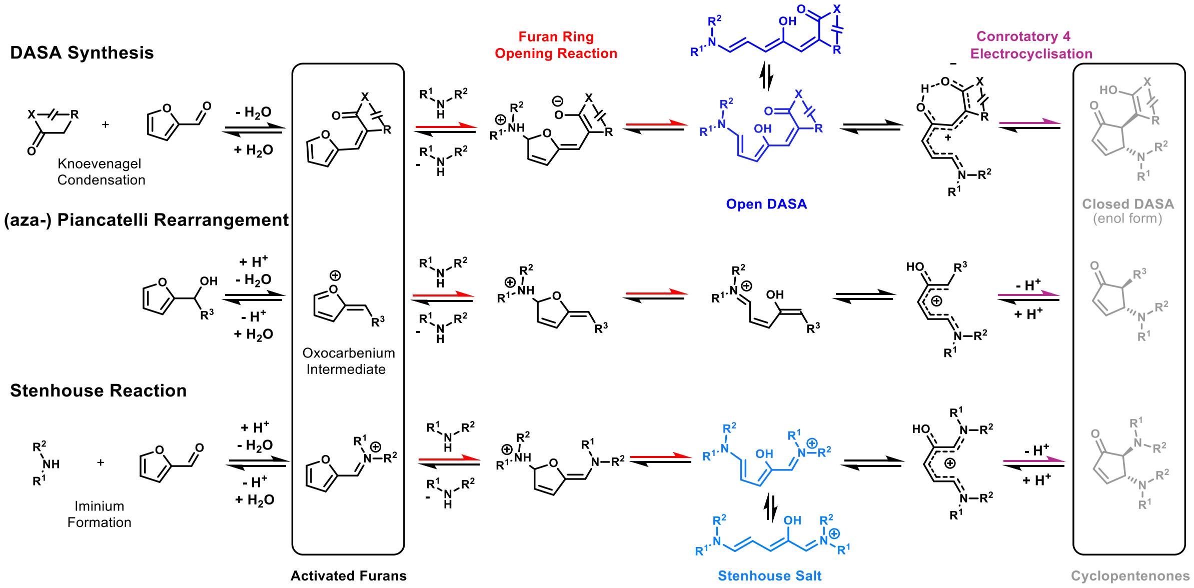

Figure S1: Similarities between the proposed mechanism of DASA synthesis, aza-Piancatelli rearrangement[1,2] and Stenhouse reaction.[3]

2.2 Furan Adducts

Compounds **1** and **S1**-S**4** were prepared similarly to literature procedures and the spectral analysis matched literature data.[4,5]

5-(furan-2-ylmethylene)-2,2-dimethyl-1,3-dioxane-4,6-dione (S1).

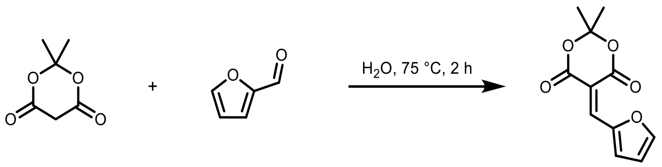

S1
(42%)

2,2-dimethyl-1,3-dioxane-4,6-dione (11.5 g, 80 mmol, 1.0 eq.) and 2-furaldehyde (7.7 g, 80 mmol, 1.0 eq.) were added to 60 mL H_2O. This suspension was heated to 75 °C and stirred for 2 h when a brown precipitate formed. The mixture was cooled to room temperature and the precipitate was collected by vacuum filtration and recrystallized from EtOH twice yielding **S1** as a yellow solid (7.5 g, 34 mmol, 42%). ^{1}H NMR (400 MHz, $CDCl_3$) δ/ppm: 8.46 (*d*, J = 3.9 Hz, 1H, C*H*CHCHO), 8.35 (*s*, 1H, OCCCH), 7.84 (*d*, J = 1.4 Hz, 1H, CHO), 6.75 (*dd*-like *m*, "J" = 3.9, 1.0 Hz, 1H, C*H*CHO), 1.77 (*s*, 6 H, 2 x CH_3); ^{13}C NMR (100 MHz, $CDCl_3$) δ/ppm: 163.4 (C=O), 160.3 (C=O), 150.5 (arom. CH), 150.4 (quart. C), 141.4 (arom. CH), 128.2 (arom. CH), 115.4 (arom. CH), 107.7 (quart. C), 104.6 (quart. C), 27.7 (CH_3); GC/EI-MS (R_t = 12.3 min): 222 (100, $M^{+\cdot}$), 165 (29), 164 (68), 120 (100, $[M–CO_2–O(CH_3)_2]^{+\cdot}$), 96 (53), 92 (59), 64 (34), 63 (59), 58 (16), 44 (27), 43 (68).

5-(furan-2-ylmethylene)-1,3-dimethylpyrimidine-2,4,6(1H,3H,5H)-trione (S2).

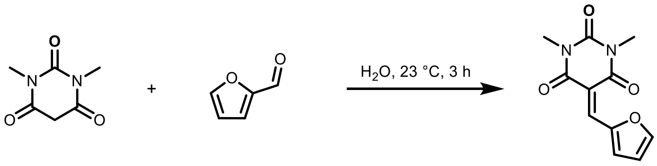

S2
(85%)

1,3-dimethylpyrimidine-2,4,6(1H,3H,5H)-trione (2.0 g, 13 mmol, 1.0 eq.) and 2-furaldehyde (1.3 g, 14 mmol, 1.1 eq.) were added to 40 mL H_2O and it was stirred for 3 h at room temperature, when reaction control by TLC showed complete consumption of the starting material. A yellow precipitate formed during the course of the reaction, which was collected by vacuum filtration and washed with ice-cooled H_2O (2 x 30 mL). The obtained solid was passed through a silica plug with DCM as the solvent. After removal of the solvent *in vacuo* **S2** was obtained as a yellow powder in a yield of 85% (2.6 g, 11 mmol). ^{1}H NMR (400 MHz, $CDCl_3$) δ/ppm: 8.63 (*d*, J = 3.8 Hz, 1H, C*H*CHCHO), 8.43 (*s*, 1H, OCCCH), 7.85 (*d*, J = 1.4 Hz, 1H, CHO), 6.74 (*ddd*-like *m*, "J" = 3.9, 1.6, 0.7 Hz, 1H, C*H*CHO), 3.41 (*s*, 3 H, CH_3), 3.40 (*s*, 3 H, CH_3); ^{13}C NMR (100 MHz, $CDCl_3$) δ/ppm: 162.6 (C=O), 161.0 (C=O), 151.5 (C=O), 151.3 (quart. C), 150.5 (arom. CH), 141.1 (arom. CH), 128.2 (arom. CH), 115.3 (arom. CH), 111.5 (quart. C), 29.1 (CH_3), 28.4 (CH_3); GC/EI-MS (R_t = 13.9 min): 234 (100, $M^{+\cdot}$), 206 (36), 149 (29), 133 (35), 120 (63), 106 (16), 93 (22), 92 (56), 66 (81), 64 (26), 63 (43).

2-phenyl-5-(trifluoromethyl)-2,4-dihydro-3H-pyrazol-3-one (S3).

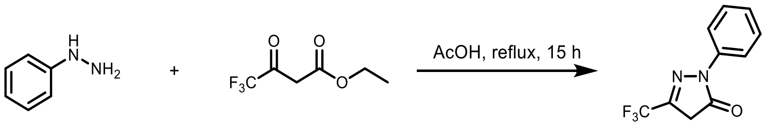

S3
(88%)

Phenylhydrazine (2.4 mL, 24 mmol, 1.0 eq.) and ethyl 4,4,4-trifluoroacetoacetate (3.5 mL, 24 mmol, 1.0 eq.) were dissolved in 6 mL AcOH and heated to reflux under N_2 for 15 h while stirring. The reaction mixture was then allowed to cool to room temperature whereupon beige crystals formed. The crystals were filtered off with a Buchner funnel, washed with H_2O (10 mL) and dried in a desiccator overnight (yield 4.8 g, 21 mmol, 88%). NMR characterization is of enol form of product. ^{1}H NMR (400 MHz, DMSO-*d*$_6$) δ/ppm: 12.43 (*br s*, 1H, OH), 7.71 (*d*-like *m*, "J"= 7.6 Hz, 2H, arom. H), 7.51 (*t*-like *m*, "J"= 7.4 Hz, 2H, arom. H), 7.38 (*tt*-like *m*, "J"= 7.4, 1.5 Hz, 1H, NCCHCHC*H*), 5.94 (*s*, 1H, CF_3CCH); ^{13}C NMR (100 MHz, DMSO-*d*$_6$) δ/ppm: 153.7 (COH), 140.4 (*q*, $^{2}J_{CF}$ = 37 Hz, *C*CF_3), 137.7 (quart. arom. C), 129.1 (arom. CH), 127.2 (NCCHCHC*H*), 122.3 (arom. CH), 121.4 (*q*, J_{CF} = 269 Hz, *C*F_3), 85.6 (*m*, $^{3}J_{CF}$ = 2 Hz, *C*HCCF_3); GC/EI-MS (R_t = 11.4 min): 228 (100, $M^{+\cdot}$), 105(23), 77(59).

4-(furan-2-ylmethylene)-3-(trifluoromethyl)isoxazol-5(4H)-one (S4).

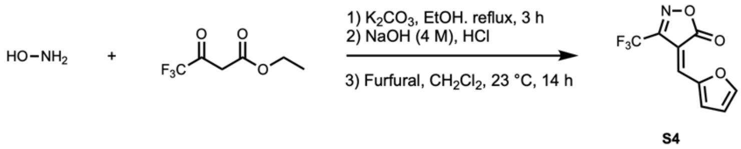

S4
(62%)

Hydroxylamine hydrochloride (1.9 g, 27 mmol, 2.3 eq.), 4,4,4-trifluoroacetoacetetate (2.4 g, 12 mmol, 1.0 eq.) and K_2CO_3 (3.8 g, 27 mmol, 2.3 eq.) were heated to reflux in EtOH (15 mL) for 3.5 hours while stirring. The reaction mixture was allowed to cool to room temperature, and the solvent was removed under reduced pressure. The yellowish residue was re-dissolved in 10 mL of an aqueous solution of NaOH (4 M) and stirred at 23 °C for 10 min. This solution was then acidified with conc. HCl to a pH value of 2 and extracted with DCM (3 x 50 mL). The organic phase was dried with $MgSO_4$, filtered and the solvent was removed *in vacuo* to afford a yellow oil (1.8 g). This oil was re-dissolved in DCM (20 mL), 2-furaldehyde (1.4 g, 15 mmol) was added, and it was stirred at 23 °C for 14 h until the mixture turned brown. H_2O (10 mL) was added and DCM was removed *in vacuo.* The precipitated brown solid was collected by filtration, rinsed with H_2O and dried in a desiccator. Purification was done by passing the product through a silica plug with DCM as an eluent. After removal of the solvent *in vacuo,* the product **S4** was obtained as a yellow solid (1.7 g, 7.4 mmol, 62%). ^{1}H NMR (400 MHz, $CDCl_3$) δ/ppm: δ 8.75 (*d*, J = 3.8 Hz, 1H, C*H*CHCHO), 7.94 (*d*, J = 1.4 Hz, CHO), 7.72 (s, 1H, CF_3CCCH), 6.86 (*ddd*, J = 3.9, 1.6, 0.7 Hz, 1H, C*H*CHO); ^{13}C NMR (100 MHz, $CDCl_3$) δ/ppm: 167.1 (C=O), 153.9 (*q*, $^{2}J_{CF}$ = 38 Hz, *C*CF_3), 152.1 (arom. CH), 150.5 (quart. C), 134.5 (*m*, $^{4}J_{CF}$ = 1 Hz, *C*HCCCF_3), 129.7 (arom. CH), 118.8 (*q*, J_{CF} = 273 Hz, *C*F_3), 116.5 (arom. CH), 106.9 (quart. C); GC/EI-MS (R_t = 9.8 min): 231 (87, $M^{+\cdot}$), 173(100), 162(29), 145(33), 125(28), 106(16), 95(28), 75(19), 63(23).

4-(furan-2-ylmethylene)-2-phenyl-5-(trifluoromethyl)-2,4-dihydro-3H-pyrazol-3-one (1).

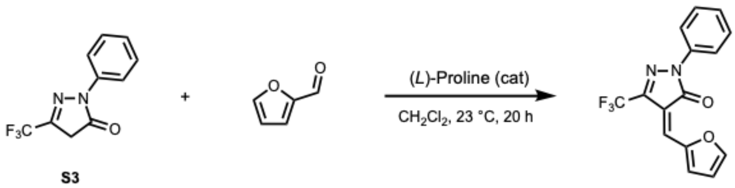

S3 1 (57%)

S3 (2.5 g, 11 mmol, 1 eq.) and 2-furaldehyde (1.9 g, 20 mmol, 1.8 eq.) were combined in 30 mL DCM and (*L*)-proline (0.21 g, 1.9 mmol, 0.1 eq.) was added. The mixture was stirred at 23 °C for 20 h and passed through a silica plug with DCM as an eluent. To the orange filtrate was added H_2O (20 mL) and this mixture was subjected to reduced pressure to remove the DCM. The precipitated product was then filtered off and rinsed with water to afford **1** as a dark orange solid (1.9 g, 6.3 mmol, 57%). ^{1}H NMR (400 MHz, $CDCl_3$) δ/ppm: 8.92 (*d*, J = 3.9 Hz, 1H, C*H*CHCHO), 7.92 (*d*, J = 7.9 Hz, 2H, NCCH), 7.87 (*d*, J = 1.3 Hz, 1H, CHO), 7.69 (*s*, 1H, CF_3CCCH), 7.46 (*t*, J = 8.0 Hz, 2H, NCCHC*H*), 7.29 – 7.26 (m, 1H, NCCHCHC*H*), 6.80 (*dd*-like *m*, "J" = 3.9, 1.3 Hz, 1H, C*H*CHO); ^{13}C NMR (100 MHz, $CDCl_3$) δ/ppm: 161.5 (C=O), 150.9 (*C*C=O), 150.8 (C*H*O), 140.0 (*q*, $^2J_{CF}$ = 37 Hz, *C*CF_3), 137.8 (*C*CHCHCHO), 131.7 (*m*, $^4J_{CF}$ = 1 Hz, *C*HCCCF_3), 129.1 (arom. CH), 128.1 (arom. CH), 126.3 (arom. CH), 120.1 (*q*, J_{CF} = 272 Hz, *C*F_3), 120.0 (arom. CH), 115.9 (N*C*CH), 115.8 (arom. CH). GC/EI-MS (R_t = 14.0 min): 306 (75, $M^{+\cdot}$), 77(100), 51(21).

2.3 Amines

Compound **S5** and **S7** were prepared according to literature procedure and spectral analysis matched literature data.[6,7]

***N*-(4-Methoxyphenyl)propane-1,3-diamine (S5).**

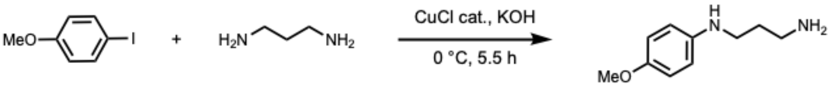

S5 (58%)

4-Iodoanisole (3.9 g, 17 mmol, 1.0 eq.), CuCl (freshly recrystallized from MeOH and dry, 0.17 g, 1.7 mmol, 0.1 eq.) and a fine and well dried powder of KOH (1.6 g, 28 mmol, 1.6 eq.) were added to a dry 100 mL round-bottom flask equipped with a stirrer and a septum under N_2. The flask was immersed into an ice-water bath and 1,3-diaminopropane (6.3 mL, 5.6 g, 76 mmol, 4.5 eq.) was added slowly at 0 °C. The reaction mixture turned dark blue overtime. After stirring at 0 °C for 5.5 h, the mixture was exposed to air and 30 mL of H_2O were added. It was extracted with DCM (3 x 150 mL) and the organic phase was dried over $MgSO_4$, filtered and solvent was removed *in vacuo* to yield the crude product as a brown oil (2.5 g). The product was purified via column chromatography on silica gel (DCM/methanol/ammonia 20:10:1) to afford **S5** as brownish oil (1.8 g, 9.9 mmol, 58%). ^{1}H NMR (400 MHz, *d*-MeOD) δ/ppm: δ 6.75 (*d-like m*, "J" = 9.1 Hz, 2H, arom. H), 6.65 (*d-like m*, "J" = 8.9 Hz, 2H, arom. H), 3.70 (*s*, 3 H, OCH_3), 3.09 (*t*, J = 7.0 Hz, 2H, CH_2), 2.75 (*t*, J = 7.2 Hz, 2H, CH_2), 1.75 (*q*, J = 7.0 Hz, 2H, $CH_2CH_2CH_2$); GC/EI-MS (R_t = 12.3 min): 180 (72, $M^{+\cdot}$), 148(17), 136(100), 123(30), 108(22).

4-(3-Aminopropoxy)-diphenylamine (S6).

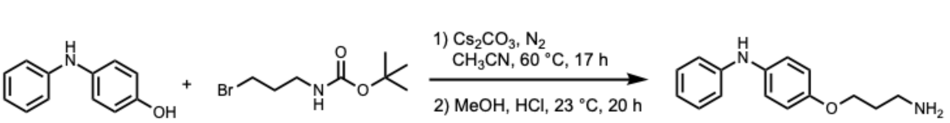

S6 (32%)

3-Bormopropylamine hydrobromide was *N*-boc protected as described in literature.[8] 4-Hydroxydiphenylamine (2.50 g, 13.5 mmol, 1.0 eq.), Cs_2CO_3 (14.6 g, 44.7 mmol, 3.3 eq.) and 3-(*N*-Boc)aminopropyl bromide (3.50 g, 14.7 mmol, 1.1 eq.) were added to a two-neck 250 mL round bottom flask equipped with a magnetic stir bar, a septum and a reflux condenser under N_2. CH_3CN (anhydrous, 75 mL) was added and the obtained suspension was heated to 60 °C under N_2 and stirred for 17 h. The mixture was then allowed to cool to room temperature and the solvent was removed *in vacuo*. The residue was taken up in AcOEt (150 mL) and extracted with H_2O (2 x 50 mL) and brine (50 mL). The organic layer was dried over $MgSO_4$, filtered and the solvent removed *in vacuo* to yield a brown solid, which was re-dissolved in MeOH (15 mL) acidified with conc. HCl (2 mL). This solution was stirred at 23 °C under N_2 for 20 h when

reaction control by TLC (AcOEt/heptane 1:2) showed complete deprotection. The solution was then cooled to 0 °C in an ice-water bath and basified with an aqueous solution of NaOH (4 M) to a pH value of 9. H_2O (15 mL) was added and a beige precipitate formed, which was collected by filtration. The product was purified via column chromatography on silica gel (DCM/methanol/ammonia 20:10:1) to afford **S6** as brownish solid (1.05 g, 4.30 mmol, 32%). ^{1}H NMR (400 MHz, $CDCl_3$) δ/ppm: 7.21 (*t-like m*, "J" = 7.9 Hz, 2H, arom. H), 7.05 (*d-like m*, "J" = 8.9 Hz, 2H, arom. H), 6.91 – 6.81 (*m*, 5 H, arom. H), 5.49 (*br s*, 1H, NH), 4.03 (*t*, J = 6.1 Hz, 2H, CH_2), 2.92 (*t*, J = 6.8 Hz, 2H, CH_2), 1.92 (*q*, J = 6.4 Hz, 2H, $CH_2CH_2CH_2$), 1.31 (*br s*, 2H, NH_2). ^{13}C NMR (100 MHz, $CDCl_3$) δ/ppm: 154.8 (quart. C), 145.3 (quart. C), 135.9 (quart. C), 129.4 (2xCH), 122.3 (2xCH), 119.7 (CH), 115.8 (2xCH), 115.5 (2xCH), 66.4 (CH_2), 39.5 (CH_2), 33.3 (CH_2); IR (ATR, cm^{-1}): 3409*w*, 3369*w*, 3249*w*, 3183*w*, 3098*w*, 3029*w*, 2998*w*, 2931*w*, 2872*w*, 2858*w*, 1596*m*, 1505*s*, 1493*s*, 1471*s*, 1445*m*, 1397*w*, 1321*m*, 1596*m*, 1225*s*, 1174*m*, 1114*m*, 1074*w*, 1025*m*, 993*w*, 941*w*, 877*m*, 818*s*, 792*m*, 741*s*, 693*s*; HR-MS (ESI+) m/z 243.1498, calc. 243.1497 for $[M + H]^+$; GC/EI-MS (R_t = 15.2 min): 242 (37, $M^{+\cdot}$), 186(23), 185(100, $[M–C_3H_8N^{\cdot}+H]^{+\cdot}$), 184(46).

Methyl indoline-2-carboxylate (S7).

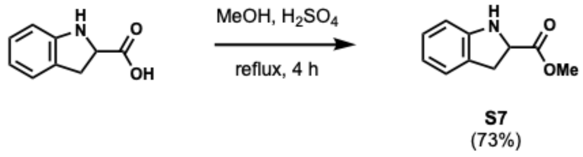

Indoline-2-carboxylic acid (2 g, 12 mmol, 1 eq.) was dissolved in methanol (25 mL). Concentrated sulfuric acid (5 mL) was added and the solution was heated to reflux for 4 h. After letting the solution cool to room temperature, it was neutralized with sodium bicarbonate and subsequently extracted with AcOEt (2 x 5 mL). After drying with $MgSO_4$, the solvent was evaporated under reduced pressure which yielded the product **S7** as a colorless oil (1.56 g, 9 mmol, 73%). The spectral analysis matched literature.[7]

2.3 DASAs

DASA-1

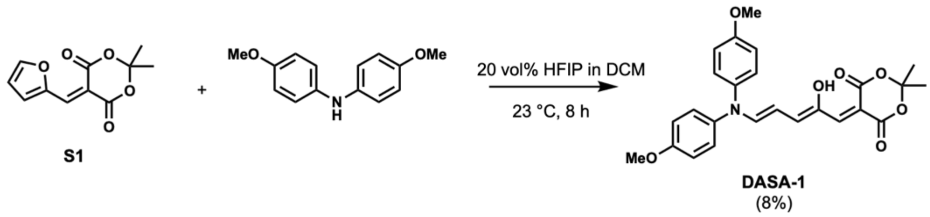

S1 (500 mg, 2.3 mmol, 1.00 eq.) and 4,4'-dimethoxydiphenylamine (500 mg, 2.2 mmol, 0.95 eq.) were dissolved in DCM (0.8 mL). HFIP (0.2 mL) were added and the solution stirred for 8 h. The solvent was removed under reduced pressure and redissolved in 1:1 AcOEt/hexanes (1 mL) and then filtered through a silica plug with first 1:9 AcOEt/hexanes and then 1:1 AcOEt/hexanes as an eluent. The second eluent was collected and the solvent removed under reduced pressure to yield the product as a dark blue solid (80 mg, 0.18 mmol, 8%). ^{1}H NMR (500 MHz, $CDCl_3$), closed isomer: δ/ppm: 7.79 (*dd*, J = 6.0, 1.9 Hz, 1H), 6.91 – 6.85 (*m*, 4H), 6.85 – 6.79 (*m*, 4H), 6.30 (*dd*, J = 6.0, 1.9 Hz, 1H), 5.65 – 5.57 (*m*, 1H), 4.06 – 4.02 (*m*, 1H) 3.77 (*s*, 6H), 3.57 – 3.52 (*m*, 1H), 1.81 (*d*, J = 8.0 Hz, 6H); ^{13}C NMR (125 MHz, $CDCl_3$) δ/ppm: 202.2, 164.8, 164.4, 162.3, 155.7, 144.1, 139.6, 133.8, 123.9, 119.6, 115.3, 114.7, 105.7, 104.0, 62.6, 55.7, 55.6, 55.6, 49.9, 44.1, 28.2, 27.6, 27.6, 27.1, 26.9; HR-MS (ESI+) m/z 474.1522, calc. 474.1525 for $[M + Na]^+$; IR (ATR, cm^{-1}): 2923, 1697, 1610, 1592, 1454, 1389, 1376, 1336, 1302, 1243, 1193, 1108, 1020, 926, 908, 826, 767, 728, 714, 680, 623, 601, 545.

DASA-2

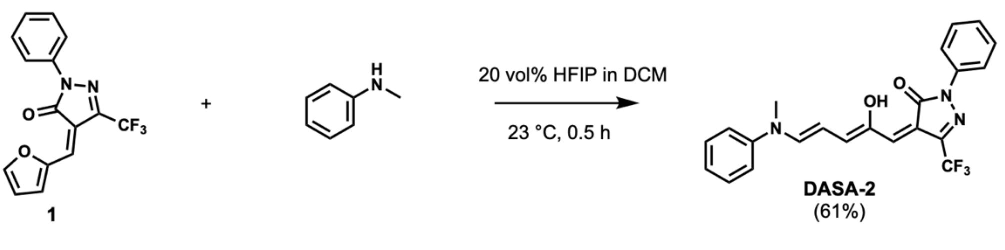

1 (100 mg, 0.32 mmol, 1.0 eq.) and *N*-methylaniline (42 mg, 0.40 mmol, 1.2 eq.) were dissolved in DCM (0.8 mL). HFIP (0.2 mL) were added and the solution stirred for 0.5 h. The solvent was removed under reduced pressure and the remaining solid triturated in diethyl ether (1.0 ml). After filtration the product was isolated as a dark blue solid (performed in triplicates: 80/81/87 mg, 0.19/0.19/0.20 mmol, 59/60/64%). ^{1}H NMR (400 MHz, $CDCl_3$) δ/ppm: 12.97 (*s*, 1H), 7.93 – 7.88 (*m*, 2H), 7.57 – 7.51 (*m*, 1H), 7.49 – 7.39 (*m*, 5H), 7.31 (*t*, J

= 7.5 Hz, 1H), 7.25 – 7.18 (*m*, 2H), 6.69 (*d*, J = 12.1 Hz, 1H), 6.63 (*s*, 1H), 6.34 (*t*, J = 12.4 Hz, 1H), 3.57 (*s*, 3H); ^{13}C NMR (125 MHz, $CDCl_3$) δ/ppm: 164.3, 163.6, 150.7, 146.9, 138.3, 134.9, 134.4, 130.0, 129.3, 129.0, 128.8, 127.8, 127.0, 125.8, 123.1, 120.5, 119.1, 114.8, 105.9, 68.1, 45.5, 33.1; HR-MS (ESI+) m/z 436.1248, calc. 436.1249 for $[M + Na]^+$; IR (ATR, cm^{-1}): 2446, 1604, 1549, 1517, 1497, 1462 1356, 1309, 1269, 1243, 1228, 1203, 1176, 1159, 1148, 1080, 960, 826, 787, 775, 755, 687.

DASA-3

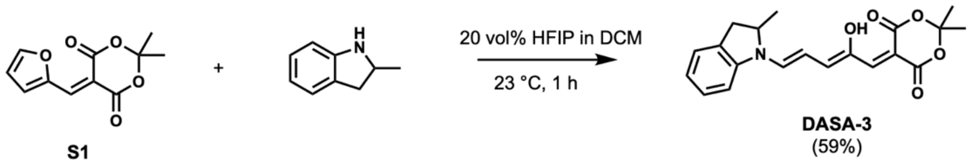

S1 (142 mg, 0.64 mmol, 1.0 eq.) and 2-methylindoline (102 mg, 0.77 mmol, 1.2 eq.) were dissolved in DCM (0.8 mL). HFIP (0.2 mL) were added and the solution stirred for 1 h. The solvent was removed under reduced pressure and the remaining solid triturated in diethyl ether (1.0 ml). After filtration the product was isolated as a dark blue solid (135 mg, 0.38 mmol, 59%). The spectroscopic data matched literature.[5]

DASA-4

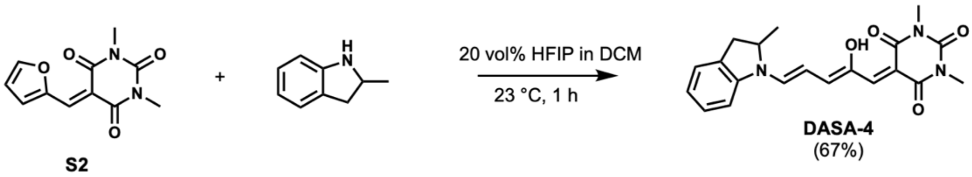

S2 (150 mg, 0.64 mmol, 1.0 eq.) and 2-methylindoline (102 mg, 0.77 mmol, 1.2 eq.) were dissolved in DCM (0.8 mL). HFIP (0.2 mL) were added and the solution stirred for 1 h. The solvent was removed under reduced pressure and the remaining solid triturated in diethyl ether (1.0 mL). After filtration the product was isolated as a dark blue solid (158 mg, 0.43 mmol, 67%). The spectroscopic data matched literature.[5]

DASA-5

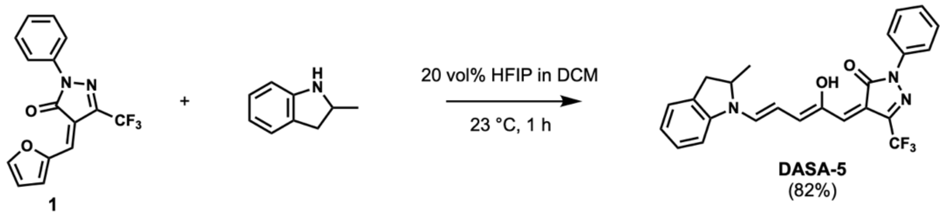

1 (100 mg, 0.32 mmol, 1.0 eq.) and 2-methylindoline (42 mg, 0.32 mmol, 1.0 eq.) were dissolved in DCM (0.8 mL). HFIP (0.2 mL) were added and the solution stirred for 1 h. The solvent was removed under reduced pressure and the remaining solid triturated in diethyl ether (1.0 mL). After filtration the product was isolated as a dark blue solid (115 mg, 0.26 mmol, 82%). The spectroscopic data matched literature.[5]

DASA-6

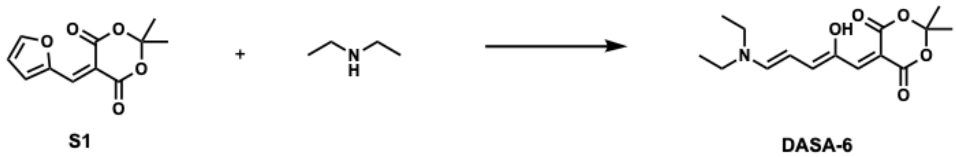

This compound was prepared analogously to literature procedures. Spectroscopic data matched literature.[4,9]

DASA-7

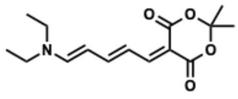

DASA-7

This compound was prepared analogously to literature procedures. Spectroscopic data matched literature.[10]

DASA-8

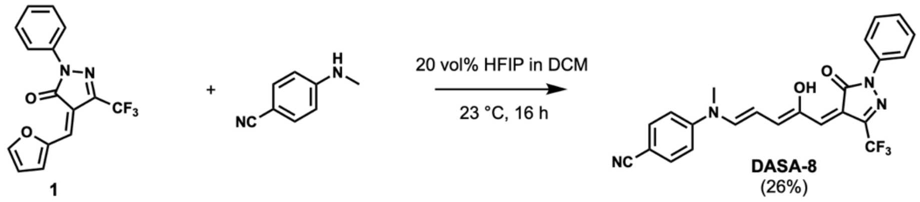

1 (231 mg, 0.75 mmol, 1.0 eq.) and 4-methylaminobenzonitrile (100 mg, 0.75 mmol, 1.0 eq.) were dissolved in DCM (0.8 mL). HFIP (0.2 mL) were added and the solution stirred for 16 h. The solvent was removed under reduced pressure and the remaining solid triturated in diethyl ether (1.0 mL). After filtration the product was isolated as a dark blue solid (84 mg, 0.19 mmol, 26%). ^{1}H NMR (500 MHz, $CDCl_3$), closed isomer: δ/ppm: 7.72 – 7.64 (*m*, 1H), 7.60 – 7.52 (*m*, 2H), 7.52 – 7.42 (*m*, 3H), 6.72 – 6.66 (*m*, 2H), 6.58 – 6.49 (*m*, 1H), 5.35 – 5.28 (*m*, 1H), 3.81 – 3.77 (*m*, 1H), 2.91 (*s*, 3H); ^{13}C NMR (125 MHz, $CDCl_3$) δ/ppm: 206.01, 163.2, 152.2, 139.6, 138.8, 137.5, 135.4, 134.0, 129.6, 128.5, 123.5, 123.3, 120.2, 113.1, 100.1, 66.3, 46.7, 32.9; HR-MS (ESI+) m/z 461.1194, calc. 461.1201 for $[M + Na]^+$; IR (ATR, cm^{-1}): 2223, 1725, 1597, 1518, 1479, 1381, 1347, 1324, 1182, 1141, 1113, 817, 753, 720, 691, 683.

DASA-9

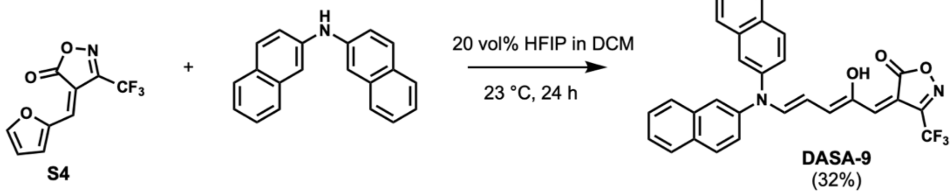

S4 (171 mg, 0.74 mmol, 1.0 eq.) and 2,2'-dinaphtylamine (200 mg, 0.74 mmol, 1.0 eq.) were dissolved in DCM (0.8 mL). HFIP (0.2 mL) were added and the solution stirred for 24 h. The solvent was removed under reduced pressure and the remaining solid triturated in diethyl ether (1.0 mL). After filtration the product was isolated as a dark blue solid (120 mg, 0.24 mmol, 32%). ^{1}H NMR (500 MHz, $CDCl_3$) δ/ppm: 11.08 – 11.04 (*m*, 1H), 7.96 – 7.90 (*m*, 2H), 7.86 – 7.80 (*m*, 2H), 7.77 – 7.74 (*m*, 2H), 7.73 – 7.67 (*m*, 2H), 7.67 – 7.56 (*m*, 1H), 7.53 – 7.50 (*m*, 3H), 7.49 – 7.44 (*m*, 1H), 7.38 – 7.32 (*m*, 1H), 7.29 – 7.22 (*m*, 1H), 6.77 (*d*, J = 12.6 Hz, 1H), 6.44 (*s*, 1H), 6.30 (*t*, J = 12.4 Hz, 1H); ^{13}C NMR (125 MHz, $CDCl_3$) δ/ppm: 175.0, 152.8, 151.7, 150.5, 148.4, 139.2, 134.6, 134.0, 129.3, 128.0, 127.7, 126.6, 126.5, 123.7, 120.3, 116.3, 112.3, 109.5, 96.4; HR-MS (ESI+) m/z 523.1233, calc. 523.1240 for $[M + Na]^+$; IR (ATR, cm^{-1}): 1660, 1597, 1483, 1457, 1375, 1336, 1291, 1210, 1183, 1133, 982, 942, 769, 739.

DASA-10

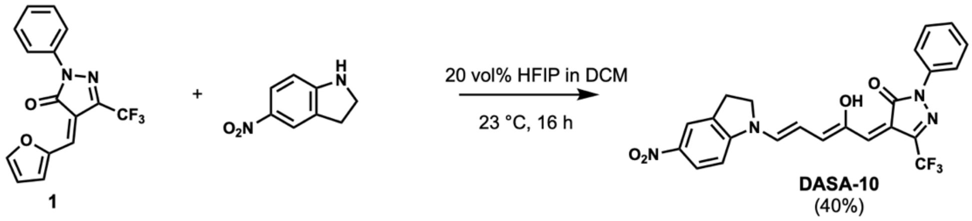

1 (100 mg, 0.32 mmol, 1.0 eq.) and 5-nitroindoline (52 mg, 0.75 mmol, 1.0 eq.) were dissolved in DCM (0.8 mL). HFIP (0.2 mL) were added and the solution stirred for 16 h. The solvent was removed under reduced pressure and the remaining solid triturated in diethyl ether (1.0 ml). After filtration the product was isolated as a dark blue solid (60 mg, 0.13 mmol, 40%). ^{1}H NMR (600 MHz, $CDCl_3$), closed isomer: δ/ppm: 7.99 – 7.92 (*m*, 2H), 7.75 – 7.70 (*m*, 1H), 7.64 – 7.61 (*m*, 2H), 7.51 – 7.38 (*m*, 3H), 6.59 – 6.55 (*m*, 1H), 6.30 – 6.24 (*m*, 1H), 5.12 – 5.07 (*m*, 1H), 3.98 – 3.95 (*m*, 1H), 3.81 – 3.74 (*m*, 1H), 3.65 – 3.55 (*m*, 1H), 3.18 – 3.11 (*m*, 2H), 2.08 – 1.94 (*m*, 1H); ^{13}C NMR (125 MHz, CDCl3) δ/ppm 162.1, 139.8, 135.6, 129.6, 129.2, 128.7, 126.3, 123.3, 120.9, 105.2, 63.6, 48.9, 44.9, 27.2; HR-MS

(ESI+) m/z 493.1096, calc. 493.1100 for $[M + Na]^+$; IR (ATR, cm^{-1}): 1615, 1599, 1562, 1536, 1493, 1470, 1453, 1364, 1320, 1248, 1214, 1196, 1152, 1107, 978, 944, 914, 899, 878, 781, 755, 742, 709, 685, 660, 582, 559.

DASA-11

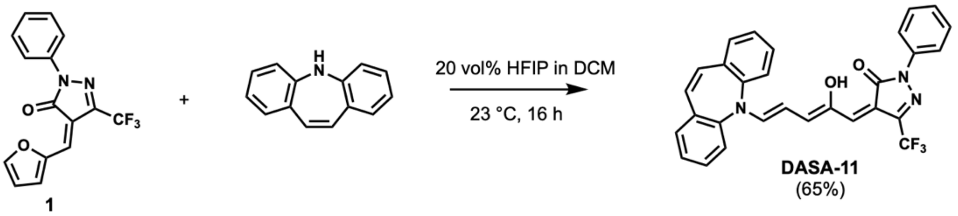

1 (200 mg, 0.65 mmol, 1.2 eq.) and 5H-dibenz[b,f]azepine (101 mg, 0.52 mmol, 1.0 eq.) were dissolved in DCM (0.8 mL). HFIP (0.2 mL) were added and the solution stirred for 16 h. The solvent was removed under reduced pressure and the remaining solid triturated in diethyl ether (1.0 mL). After filtration the product was isolated as a dark blue solid (170 mg, 0.34 mmol, 65%). ^{1}H NMR (400 MHz,$CDCl_3$) δ/ppm: 12.81 – 12.77 (*m*, 1H), 7.96 – 7.85 (*m*, 2H), 7.59 – 7.48 (*m*, 3H), 7.50 – 7.34 (*m*, 8H), 7.25 – 7.16 (*m*, 1H), 6.96 – 6.90 (*m*, 2H), 6.63 – 6.55 (*m*, 2H), 6.18 (*t*, J = 12.5 Hz, 1H); ^{13}C NMR (125 MHz, $CDCl_3$) δ/ppm: 164.3, 155.7, 150.6, 146.8, 140.2, 139.9, 138.3, 135.5, 133.3, 132.1, 130.3, 129.7, 129.5, 128.8, 125.8, 123.0, 120.5, 119.9, 119.3, 115.6, 105.1; HR-MS (ESI+) m/z 554.1666, calc. 554.1667 for $[M + Na+ MeOH]^+$; IR (ATR, cm^{-1}): 1607, 1554, 1497, 1453, 1430, 1347, 1302, 1202, 1182, 1148, 1108, 1001, 984, 962, 944, 875, 757, 741, 684, 653, 640, 600, 555.

DASA-12

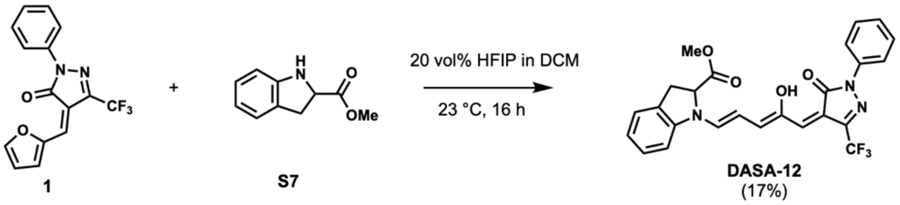

1 (200 mg, 0.65 mmol, 1.1 eq.) and **S7** (104 mg, 0.58 mmol, 1.0 eq.) were dissolved in DCM (0.8 mL). HFIP (0.2 mL) were added and the solution stirred for 16 h. The solvent was removed under reduced pressure and the remaining solid triturated in diethyl ether (1.0 mL). After filtration the product was isolated as a dark blue solid (48 mg, 0.10 mmol, 17%).^{1}H NMR (500 MHz, $CDCl_3$) δ/ppm: 7.75 (*d*, J = 7.9 Hz, 2H), 7.54 (*d*, J = 6.1 Hz, 1H), 7.46 (*t*, J = 7.8 Hz, 3H), 7.36 (*q*, J = 7.8 Hz, 1H), 7.17 – 7.06 (*m*, 2H), 6.80 (*t*, J = 7.4 Hz, 1H), 6.54 (*d*, J = 6.1 Hz, 1H), 6.46 (*d*, J = 8.0 Hz, 1H), 4.95 – 4.90 (*m*, 1H), 4.37 (*dd*, J = 11.5, 5.6 Hz, 1H), 3.89 – 3.81 (*m*, 4H), 3.75 – 3.60 (*m*, 3H), 3.23 (*dd*, J = 16.5, 5.6 Hz, 1H); ^{13}C NMR (125 MHz, $CDCl_3$) δ/ppm: 202.4, 158.9, 152.1, 149.7, 137.7, 136.4, 129.0, 128.4, 127.5, 126.5, 124.6, 123.0, 120.3, 109.0, 95.4, 68.0, 60.8, 53.8, 46.5, 34.6; HR-MS (ESI+) m/z 506.1297, calc. 506.1304 for $[M + Na]^+$; IR (ATR, cm^{-1}): 1739, 1610, 1557, 1489, 1461, 1431 1350, 1315, 1243, 1210, 1162, 1147, 1116, 1042, 984, 961, 945, 869, 835, 788 763, 743, 710, 692, 665, 647.

DASA-13

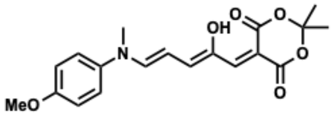

DASA-13

This compound was prepared analogously to literature procedures. Spectroscopic data matched literature.[11]

DASA-14

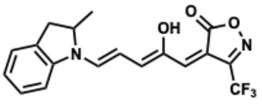

DASA-14

This compound was prepared analogously to literature procedures. Spectroscopic data matched literature.[5]

2.4 Polymers

Compounds **S8**-**S10** were prepared similarly to literature procedures.[12]

P(BMA-PFPMA) (S8)

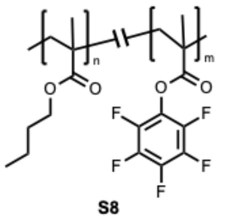

S8
m = 4 mol%

The monomers were purified from their polymerization inhibitors by passing them through a basic aluminum oxide plug. Butyl methacrylate (4.95 g, 34.8 mmol, 1.00 eq.), pentafluorophenyl methacrylate (0.370 g, 1.45 mmol, 0.04 eq.) and azobis(isobutyronitrile) (AIBN, recrystallized from MeOH, 70 mg, 0.43 mmol, 0.01 eq.) were dissolved in 10 mL of dry 1,4-dioxane in a round bottom flask equipped with a rubber septum and a magnetic stir bar. The mixture was deoxygenated by bubbling N_2 gas through for 30 minutes. The flask was then immersed into a pre-heated oil bath and the reaction mixture was stirred at 80 °C under nitrogen for 22 h. The solution was allowed to cool to room temperature and diluted with 10 mL of THF. The polymer was precipitated into ice-cold MeOH (175 mL) under rapid stirring, filtered off, washed with MeOH and dried under vacuum to yield **S8** as colorless solid (yield: 4.36 g). ^{1}H NMR (400 MHz, $CDCl_3$) δ/ppm: 3.94 (*d*-like *m*, OCH_2), 2.00 – 1.81 (*m*, CH_2), 1.61 (*br s*, CH_2), 1.40 (*d*-like *m*, CH_2), 1.28 – 1.14 (*br*, CH_3), 1.02 (*br s*, CH_3), 0.95 (*br s*, CH_3), 0.87 (*br s*, CH_3); ^{19}F NMR (377 MHz, $CDCl_3$) δ/ppm: -149.5 (*s*, 1F), -151.4 (*s*, 1F), -(157.7 – 157.9) (*m*, 1F), -(162.1 – 162.3) (*m*, 2F); IR (ATR, cm^{-1}): 2957*m*, 2932*m*, 2873*w*, 1777w (C=O, PFP ester), 1722*s* (C=O, butyl ester), 1519*m* (aryl C-F), 1466*m*, 1450*m*, 1382*w*, 1264*m*, 1239*m*, 1171*m*, 1142*s*, 1063*m*, 993*m*, 964*m*, 945*m*, 843*w*, 748*m*; GPC: M_n= 30.3 kDa, PDI =2.7.

P(BMA-S6MA) (S9)

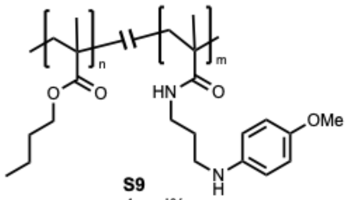

S9
m = 4 mol%

S8 (1.0 g) was dissolved in 10 mL of anhydrous DMF in a Schlenk tube equipped with rubber septum and magnetic stir bar. **S5** (0.23 g, 1.3 mmol) and TEA (0.20 mL, 0.15 g, 1.5 mmol) were dissolved in 2 mL of anhydrous DMF and added to the polymer solution. The reaction mixture was stirred at 50 °C under nitrogen atmosphere for 5 days until completion. The reaction progress was monitored via IR spectroscopy (disappearance of PFP ester band at 1777 cm^{-1}) of samples precipitated from the reaction mixture by addition to ice-cooled MeOH. After completion of the reaction, the polymer was precipitated into ice-cold methanol/water (5:1, 100 mL) with a few drops of concentrated ammonia and centrifuged. The polymer pellet was dissolved in THF and re-precipitated twice and then dried under vacuum to afford **S9** as white solid (0.98 g). Full conversion of the PFP active ester was confirmed via ^{19}F NMR from the complete disappearance of the fluorine signals. ^{1}H NMR (400 MHz, $CDCl_3$) δ/ppm: 6.78 (*d*, J = 8.3 Hz, 2 arom. H), 6.63 (*d*, J = 8.1 Hz, 2 arom. H), 6.35 – 6.25 (*br*, N-H, amide), 3.94 (*m*, OCH_2), 3.74 (*m*, OCH_3), 3.30 (*br s*, OCNHC*H*$_2$), 3.16 (*br s*, CHCNHC*H*$_2$), 2.00 – 1.81 (*m*, CH_2), 1.59 (*br s*, CH_2), 1.40 (*d*-like *m*, CH_2), 1.28 – 1.21 (*br*, CH_3), 1.02 (*br s*, CH_3), 0.94 (*br s*, CH_3), 0.86 (*br s*, CH_3); IR (ATR, cm^{-1}): 3418*w*, 2956*m*, 2932*m*, 2872*w*, 1721*s*, 1583*w*, ,1530*w*, 1465*m*, 1381*w*, 1264*m*, 1239*m*, 1142*s*, 1062*m*, 1019*w*, 963*m*, 944*m*, 844*w*, 769m, 748*m*; UV-Vis (DCM) λ_{max} = 312 nm; GPC: M_n= 32.0 kDa, PDI =2.9.

P(BMA-S7MA) (S10)

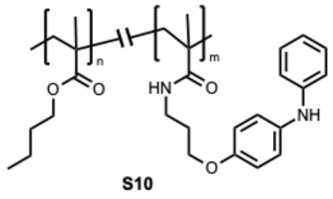

S10
m = 4 mol%

Same procedure as for **S9** using **S8** (1.0 g) and **S6** (0.26 g, 1.1 mmol). Yield: 0.86 g of an off-white solid. ^{1}H NMR (400 MHz, $CDCl_3$) δ/ppm: 7.21 (*t*, J = 7.5 Hz, 2 arom. H), 7.06 (*d*-like *m*, "J"= 7.1 Hz, 2 arom. H), 6.92 (*d*, J =7.7 Hz, 2 arom. H), 6.85–6.81 (*m*, 3 arom. H), 3.93 (*m*, OCH_2 butyl), 3.38 (*br s*, OCNHC*H*$_2$), 1.99 – 1.81 (*m*, CH_2), 1.60 (*br s*, CH_2), 1.39 (*d*-like *m*, CH_2), 1.28 – 1.21 (*br*, CH_3),

1.02 (*br s*, CH_3), 0.95 (*br s*, CH_3), 0.87 (*br s*, CH_3); IR (ATR, cm^{-1}): 3420*w*, 2956*m*, 2932*m*, 2872*w*, 1722*s*, 1666w, 1600*w*, 1511*w*, 1465*m*, 1385*w*, 1238*m*, 1142*s*, 1062*m*, 1019*m*, 996*w*, 963*m*, 944*m*, 843*w*, 747*m*, 693*w*; UV-Vis (DCM) λ_{max} = 284 nm; GPC: M_n= 35.5 kDa, PDI =2.7.

P1

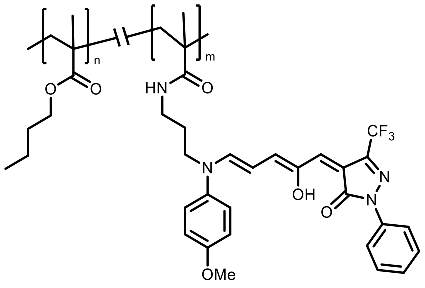

P1
m = 4 mol%

To **S9** (0.25 g) dissolved in DCM (1.5 mL) in a screw cap vial was added **1** (0.30 g, 1 mmol, 0.2 g/mL) and HFIP (0.38 mL). It was stirred at 23 °C in the dark for 5 h. The reaction was monitored via UV-Vis spectroscopy by taking samples and was stopped when the absorbance value of the DASA peak plateaued. Subsequently, the solvent was removed *in vacuo* and the polymer was purified by size exclusion chromatography over Bio-Beads S-X1 Support with distilled THF as the eluent. The excess of **1** could be recovered und reused after separation from the polymer. **P1** was obtained as a deep blue solid (0.25 g). IR (ATR, cm^{-1}): 3420*w*, 2956*w*, 2932*w*, 2872*w*, 1721*s*, 1600*w*, 1558*w*, 1471*m*, 1455*m*, 1379*w*, 1239*m*, 1145*s*, 1114*s*, 1062*m*, 1020*w*, 983*m*, 944*m*, 878*w*, 837*w*, 710*w*, 785*m*, 747*m*, 710*m*; NMR data in section *11.1*.

P2

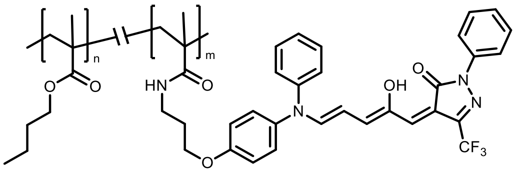

P2
m = 4 mol%

Same procedure as for **P1** using **S10** (0.20 g) and **1** (0.30 g, 1 mmol, 0.2 g/mL) reacting overnight giving **P2** as a deep blue solid (0.19 g). IR (ATR, cm^{-1}): 3420*w*, 2956*w*, 2932*w*, 2872*w*, 1722*s*, 1598*w*, 1464*m*, 1455*m*, 1385*w*, 1356*w*, 1238*m*, 1142*s*, 1062*m*, 1017*w*, 945*m*, 841*w*, 748*m*, 692*w*; NMR data in section *11.1*.

3. Kinetics Experiments

3.1 UV-Vis Spectroscopy

In situ UV-Vis kinetic experiments were performed between 23–25 ºC under pseudo-first order conditions using a 100-fold excess of amine reagent. Stock solutions of amine and furan adduct in DCM were freshly prepared and measurements were performed by adding small amounts of stock solutions to the respective DCM/HFIP mixture in a 3 mL quartz cuvette. The absorbance was monitored with an Agilent Cary 4000 UV-Vis spectrophotometer overtime.

3.1.1 Rates as Function of HFIP Concentration

Kinetic experiments were done using *N*-methylaniline and **1** for different volumetric ratios of HFIP in DCM (0–90 vol%). Measurements were performed at concentrations of $5x10^{-3}$ M (amine) and $5x10^{-5}$ M (furan adduct) at the absorption maximum of **1** (375 nm) and at 565 nm over the time course of 15 to 800 min (**Figure S2**). Apparent rate constants were obtained by fitting the absorbance changes to one-phase exponential decay functions using Origin 2018 software. R^2-values reached >0.99 for HFIP concentrations <50 vol%. At concentrations >50 vol% the absorbance changes are not strictly mono-exponential anymore and the rate of DASA formation decreases suggesting the presence of side reactions or degradation of the activated furan. Relative rates ($k_{relative}$) correspond to the apparent rate constants at a given HFIP concentration relative to the apparent rate constant of the reaction in pure DCM (**Figure S2d**). Note: due to

the lower concentrations used in UV-Vis spectroscopic measurements the amount of HFIP relative to **1** was considerably higher than in the NMR kinetic experiments (i.e. 1 vol% HFIP corresponds to a large excess of ~1900 equivalents).

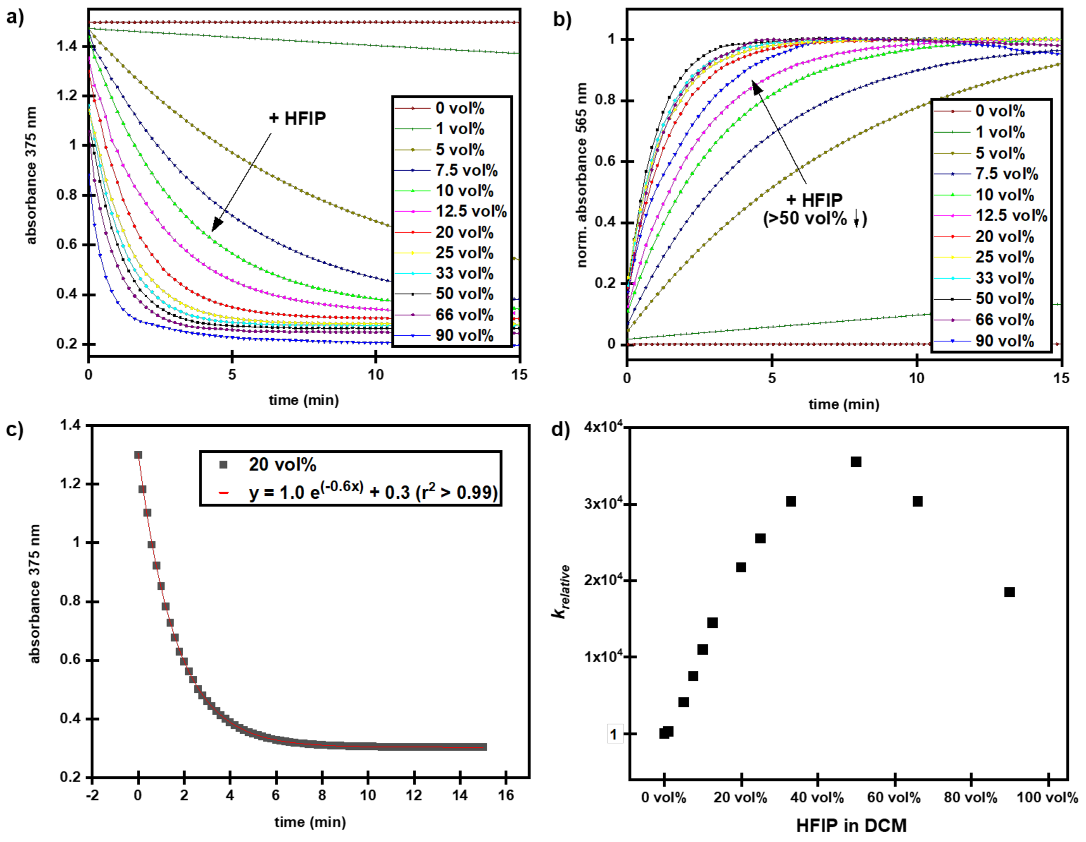

Figure S2. UV-Vis kinetic data for the reaction of **1** with *N*-methylaniline as a function of the HFIP concentration in DCM. a) Absorbance change at λ_{max} (375 nm) of **1** as a function of time. b) Absorbance change at 565 nm as a function of time. c) Example of non-linear fit of absorbance decay at 375 nm. d) Relative reaction rates as a function of the HFIP concentration.

3.1.2 Alkyl vs Aryl Amines

The rate of diethylamine (DEA) and *N*-methylaniline in the reaction with **1** was compared in DCM and DCM/HFIP 20 vol%. Measurements were performed at concentrations of 12.5x10^{-4} M (amine) and 12.5x10^{-6} M (furan adduct) at λ_{max} of the formed DASA in the respective solvent system (with *N*-methylaniline λ_{max}(DCM) = 614 nm, λ_{max}(DCM/HFIP) = 566 nm; with DEA λ_{max}(DCM) = 580 nm, λ_{max}(DCM/HFIP) = 520 nm).

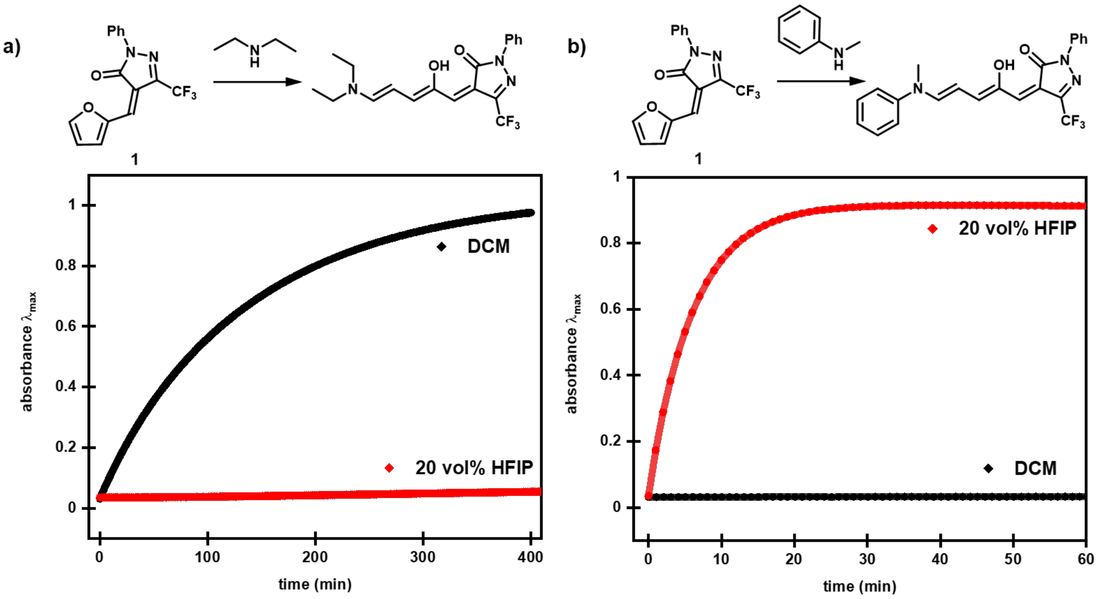

Figure S3: Absorbance change at λ_{max} (open form DASA) overtime for the reaction of **1** with a) DEA and b) *N*-methylaniline in DCM vs. DCM/HFIP 20 vol%.

3.1.3 Polymers

Experiments were done by preparing stock solutions of the amine modified polymer **S9** (1 mg/mL) and **1** (1.5 mM) in THF and DCM, respectively. Measurements were performed at concentrations of 0.1 mg/mL (polymer) and 1.5×10^{-4} M (furan adduct) in THF or DCM/HFIP 20 vol% in 3 mL quartz cuvettes and the absorbance at λ_{max} of the formed DASA in the respective solvent system for 800 min (λ_{max}(THF) = 615 nm, λ_{max}(DCM/HFIP) = 566 nm).

3.2 NMR Spectroscopy

In situ NMR experiments were performed at a concentration of 25 mM and 298 K. A solution of **1** in CD_2Cl_2 (0.35 mL, 50 mM, 1.0 eq.) and a solution of *N*-methylaniline in CD_2Cl_2 (0.35 mL, targeted at 55 mM, 1.1 eq.) were prepared and to the latter was added deuterated HFIP (0, 1 and 5 eq. relative to **1** or 0, ~0.2 and ~1 vol%). These solutions were combined in a standard 5 mm NMR tube and a series of 300 spectra was acquired on a time course of up to 15 hours on a Varian Unity Inova AS600 600 MHz spectrometer (delay before start of the first scan was 5 min). Conversion plots were calculated from the integrals of the signals of **1** at δ = 8.89 ppm and the signals of the *N*-methyl groups in the open (δ = 3.59 ppm) and closed (δ = 2.85 ppm) DASA respectively as given by:

$$Conversion = \frac{\mathbf{a}}{\mathbf{a} + \frac{\mathbf{b}}{3} + \frac{\mathbf{c}}{3}}$$

a = integrated signal of **1** (*d*, 1H, OCH, 8.89 ppm)
b = integrated signal *N*-Me group open form (*s*, 3H, CH_3, 3.59 ppm)
c = integrated signal *N*-Me group closed form (*s*, 3H, CH_3, 2.85 ppm)

The measurements were performed in triplicates. The actual amine concentrations (as determined by ^{1}H-NMR) varied between 1.1–1.5 equivalents relative to **1**. Analogous measurements were performed in acetonitrile-d_3 or by exchanging HFIP-d_2 with isopropanol or the methyl-ether of HFIP (HFIPME) (**Figure S4** and **Figures S8**–**S9**). Similar measurements on the uncatalyzed runs at higher concentration and longer reaction time (50 mM, 15 h) additionally confirmed that similar conversions are reached when the curve plateaus as for the runs with HFIP (80–85%). Also, it was found that higher excess of amine can push the conversion to >95%.

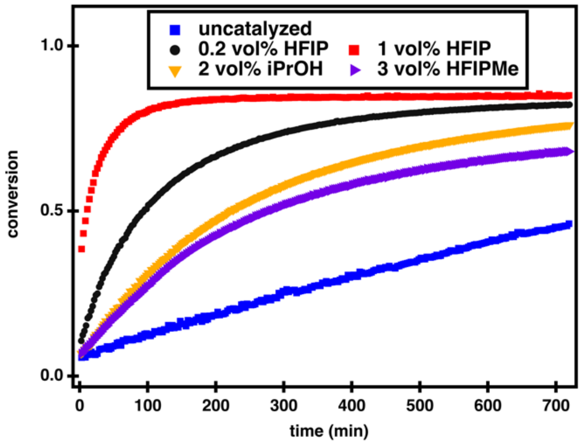

Figure S4: Conversion plots from *in situ* NMR experiments on the synthesis of DASA-**2** in DCM (*k*: 3 M^{-1} h^{-1}) and upon addition of small quantities of different additives: iPrOH (2 vol%, *k*: 9 M^{-1} h^{-1}), Me-ether of HFIP (HFIPMe, 3 vol%, *k*: 5 M^{-1} h^{-1}), HFIP (0.2 vol%, *k*: 12 M^{-1} h^{-1} and 1 vol%, *k*: 56 M^{-1} h^{-1}). Note: reactions were performed with similar excess of *N*-methylaniline (1.1–1.2 equivalents), except for the experiment with HFIPMe (1.5 equivalents).

3.2.1 Kinetic Fitting

To determine rate constants second-order kinetics was assumed. For this the following differential equation applies:

$$\frac{\mathrm{d}[N\text{-methylaniline}]}{\mathrm{dt}} = -k[\mathbf{1}][N\text{-methylaniline}]$$

The corresponding linearized integrated rate equation is:

$$\ln\frac{[N\text{-methylaniline}]}{[\mathbf{1}]} = k([N\text{-methylaniline}]_0 - [\mathbf{1}]_0)\mathrm{t} + \ln\frac{[N\text{-methylaniline}]_0}{[\mathbf{1}]_0}$$

with [x] being the concentration of compound x as a function of time (t) and $[x]_0$ being the initial concentration of compound x. The rate constant *k* was calculated from the slope of the regression line in the linear regime of a ln([*N*-methylaniline]/[**1**]) versus time plot (**Figures S5**–**S9**). The initial ratio of *N*-methylaniline to **1** was calculated through the x-axis intersection.

Table S1: Determined rate constants in dichloromethane.

**Additive**[a]	***k*** **(M^{-1} h^{-1})**
-	3 ± 1[b]
0.2 vol% (1 eq.) HFIP	11.6 ± 0.2[b]
1 vol% (5 eq.) HFIP	56 ± 5[b]
2 vol% (10 eq.) iPrOH	9
3 vol% (10 eq.) HFIPMe	5

[a] Equivalents relative to **1**. [b] Mean values of three measurements ± standard deviation.

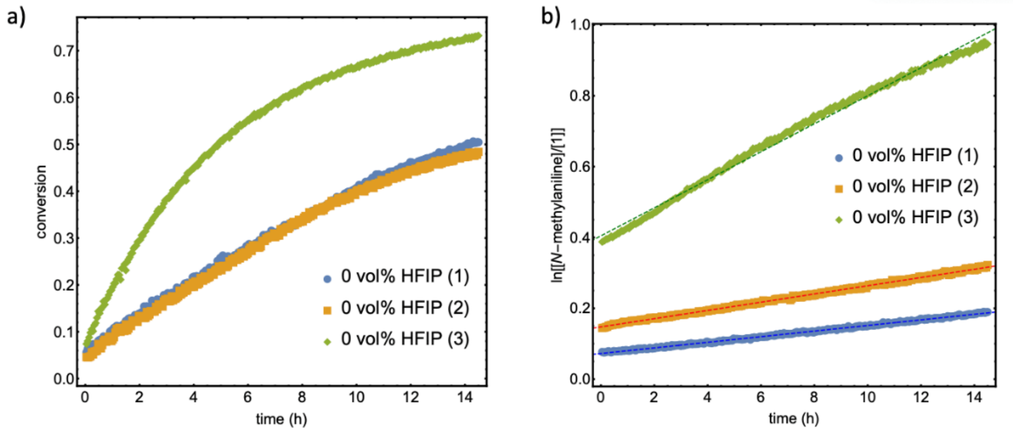

Figure S5: Kinetic fitting of uncatalyzed reaction of **1** with *N*-methylaniline in dichloromethane. a) Conversion of each measurement (1) 1.1 eq. (2) 1.2 eq. (3) 1.5 eq. of *N*-methylaniline. The increase in conversion of (3) in *a)* is due to the increased excess in *N*-methylaniline. b) Second-order kinetic fitting for each experiment. Calculated *k*: (1) 4.2 M^{-1} h^{-1}; (2) 1.9 M^{-1} h^{-1}; (3) 3.2 M^{-1} h^{-1}; 3 ± 1 M^{-1} h^{-1}.

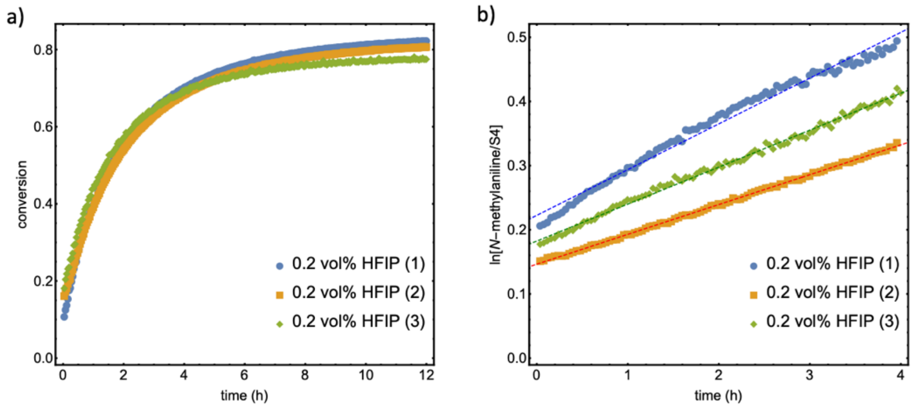

Figure S6: Kinetic fitting of the reaction of **1** with *N*-methylaniline in presence of 0.2 vol% (or 1 eq. relative to **1**) HFIP in dichloromethane. a) Conversion of each measurement (1) 1.3 eq. (2) 1.2 eq. (3) 1.2 eq. of *N*-methylaniline. b) Second-order kinetic fitting for each experiment. Calculated *k*: (1) 11.5 M^{-1} h^{-1}; (2) 11.8 M^{-1} h^{-1}; (3) 11.5 M^{-1} h^{-1}; 11.6 ± 0.2 M^{-1} h^{-1}.

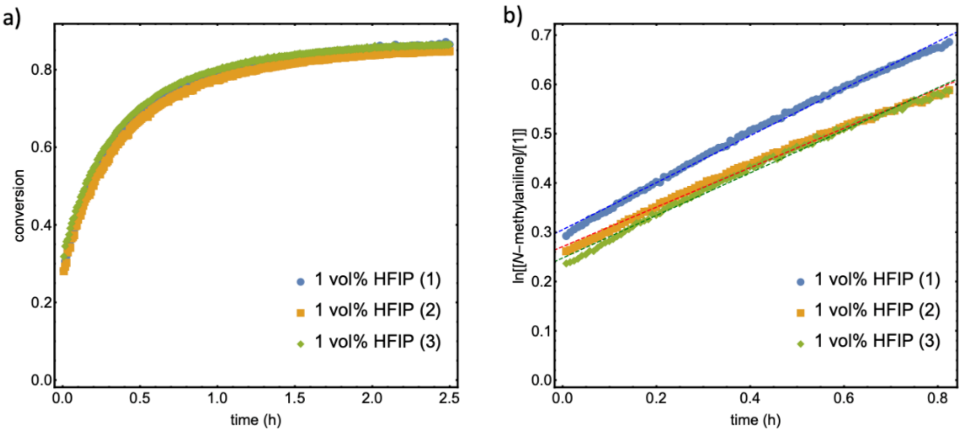

Figure S7: Kinetic fitting of the reaction of **1** with *N*-methylaniline in presence of 1 vol% (or 5 eq. relative to **1**) HFIP in dichloromethane. a) Conversion of each measurement (1) 1.4 eq. (2) 1.3 eq. (3) 1.3 eq. of *N*-methylaniline. b) Second-order kinetic fitting for each experiment. Calculated *k*: (1) 53.5 M^{-1} h^{-1}; (2) 51.5 M^{-1} h^{-1}; (3) 61.4 M^{-1} h^{-1}; 56 ± 5 M^{-1} h^{-1}.

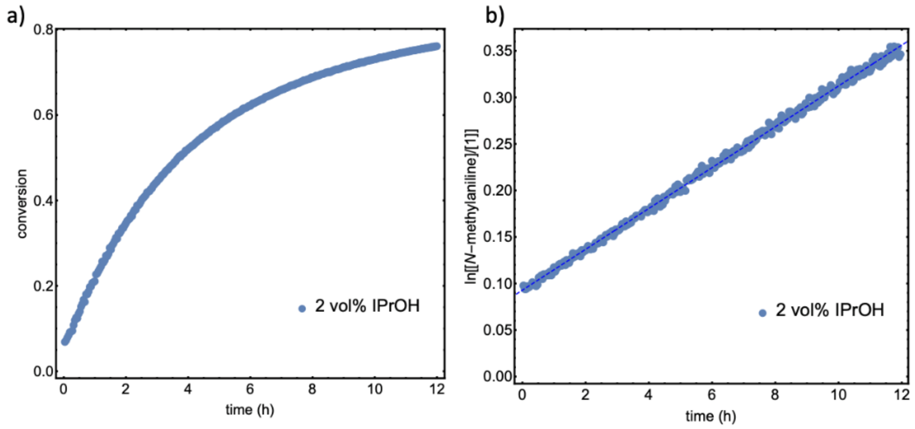

Figure S8: Kinetic fitting of the reaction of **1** with *N*-methylaniline in presence of 2 vol% (or 10 eq. relative to **1**) iPrOH in dichloromethane. a) Conversion, 1.1 eq. of *N*-methylaniline. b) Second-order kinetic fitting. Calculated *k*: 9 M^{-1} h^{-1}.

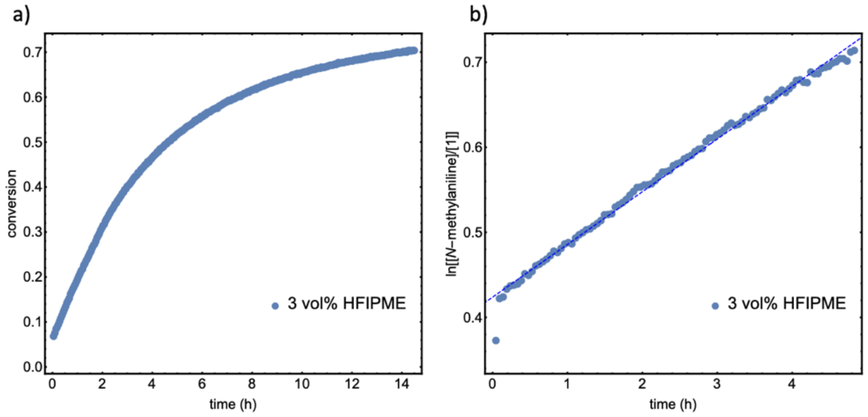

Figure S9: Kinetic fitting of the reaction of **1** with *N*-methylaniline in presence of 3 vol% (or 10 eq. relative to **1**) HFIPME in dichloromethane. a) Conversion, 1.5 eq. of *N*-methylaniline. b) Second-order kinetic fitting. Calculated *k*: 5 M^{-1} h^{-1}.

4. UV-Vis Absorption Spectra of Furan Adducts

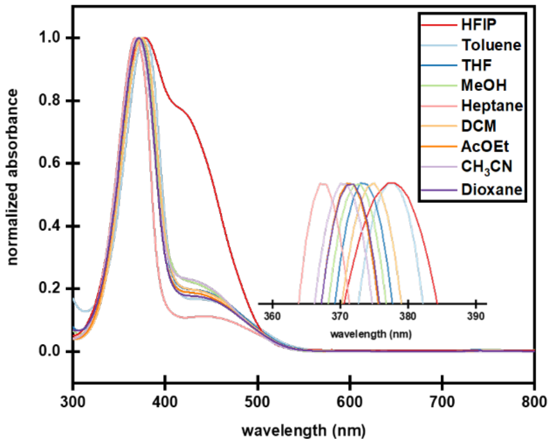

Figure S10: Normalized UV-Vis spectra of **1** in different solvents.

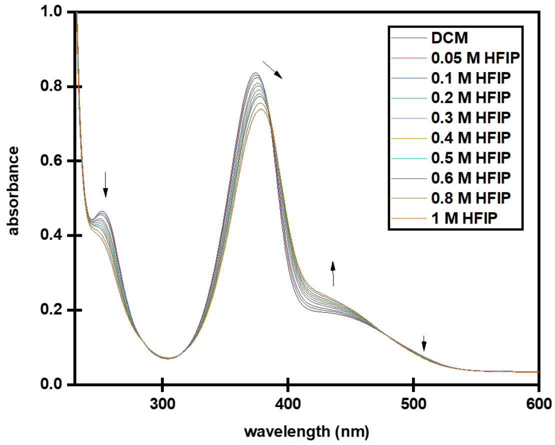

Figure S11: UV-Vis spectral changes during titration of **1** in DCM (2.5x10^{-5} M) with HFIP. The concentration of HFIP is indicated in the legend.

5. Effects on Thermodynamic Equilibrium

5.1 NMR Spectroscopy

To analyze closed form formation during synthesis NMR spectra from kinetic experiments in *section **3.2*** were compared at 2.5 h for differing conditions ("t= 0 h" refers to the first scan measured 5 min after setting up the reaction (**Figures S12–S17**).

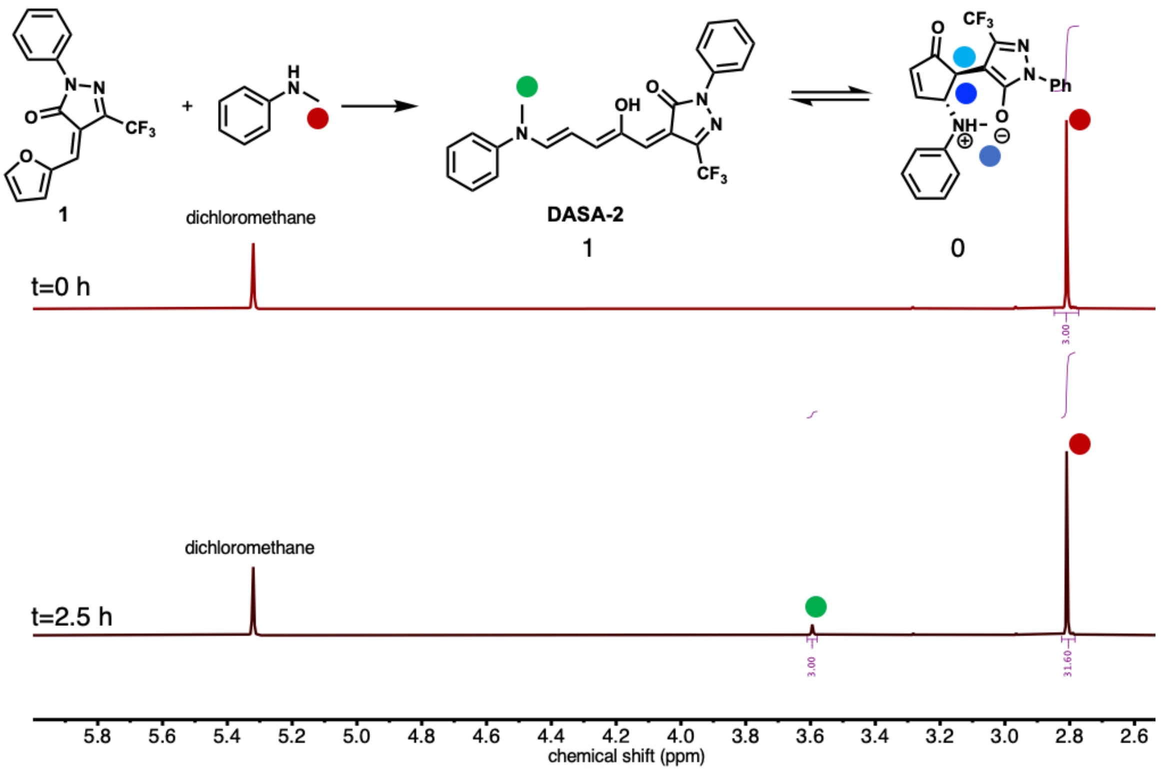

Figure S12: ^{1}H NMR (600 MHz, CD_2Cl_2) spectra from kinetic experiments for DASA-**2** in dichloromethane at t=0 and t= 2.5 h. No closed form can be observed.

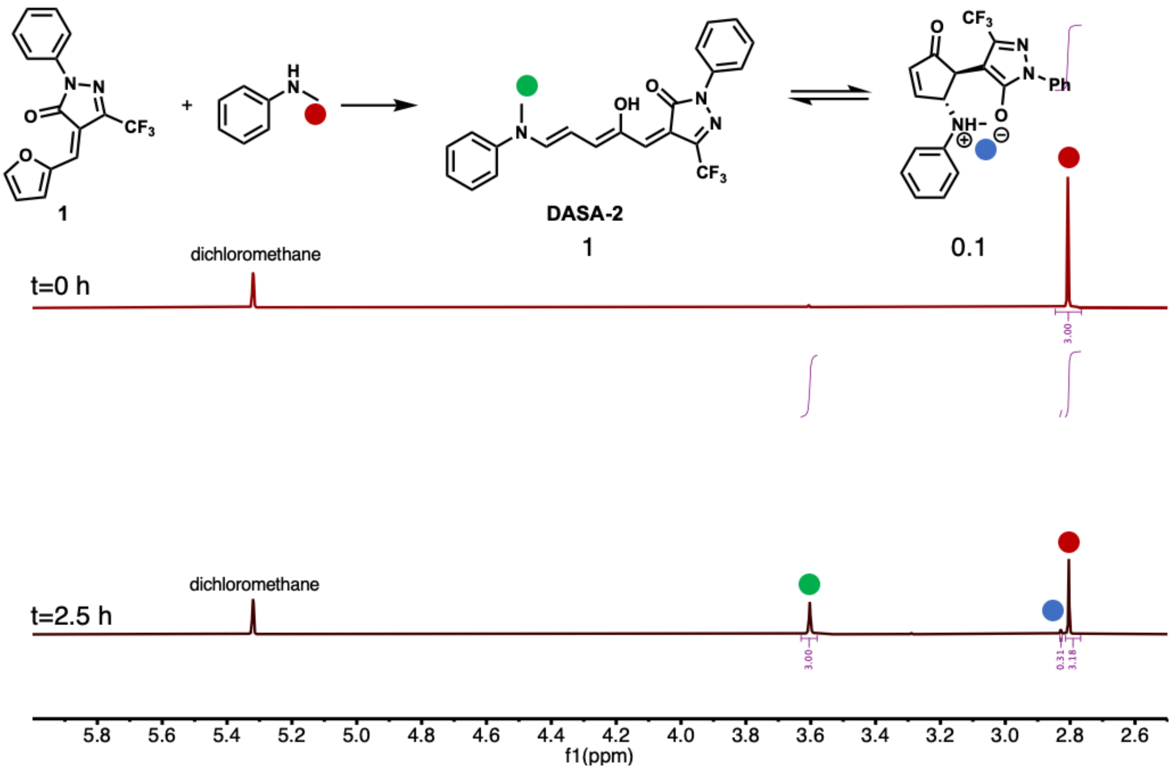

Figure S13: ^{1}H NMR (600 MHz, CD_2Cl_2) spectra from kinetic experiments for DASA-**2** in dichloromethane at t=0 and t= 2.5 h with 1 eq. of HFIP regarding **1**. 9% closed form can be observed.

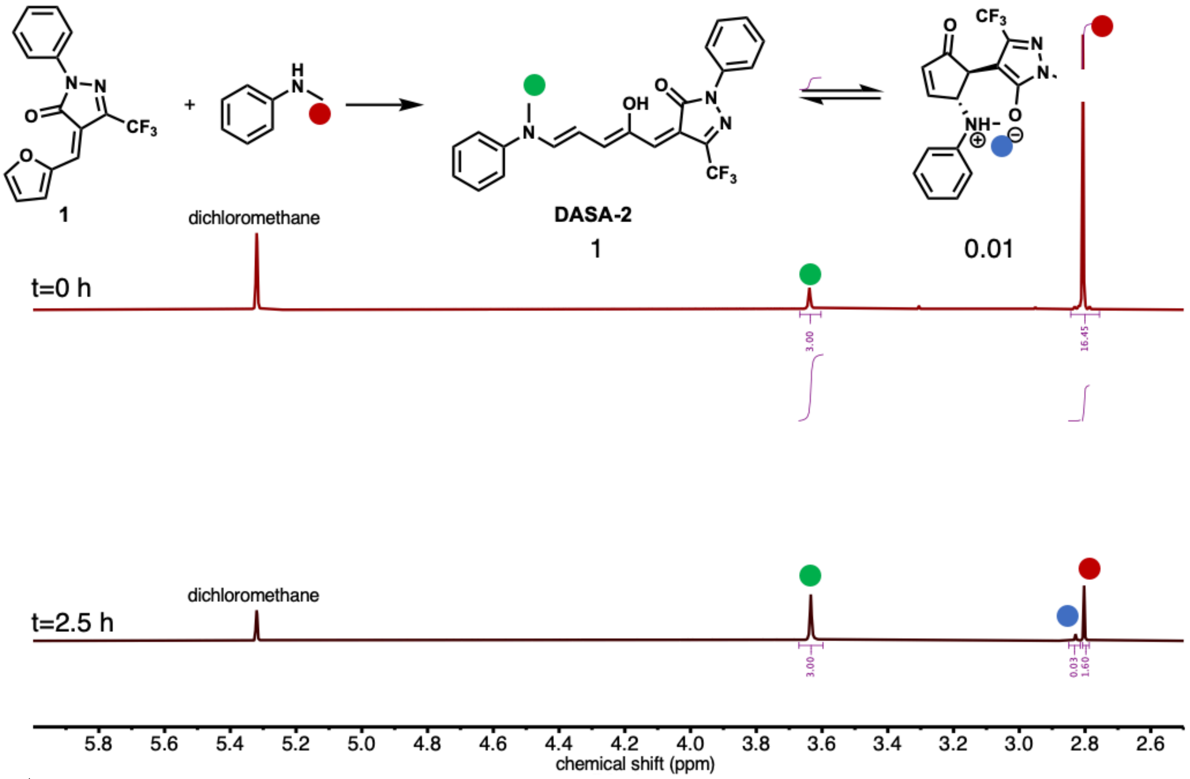

Figure S14: ^{1}H NMR (600 MHz, CD_2Cl_2) spectra from kinetic experiments for DASA-**2** in dichloromethane at t=0 and t= 2.5 h with 5 eq. of HFIP regarding **1**. 1% closed form can be observed.

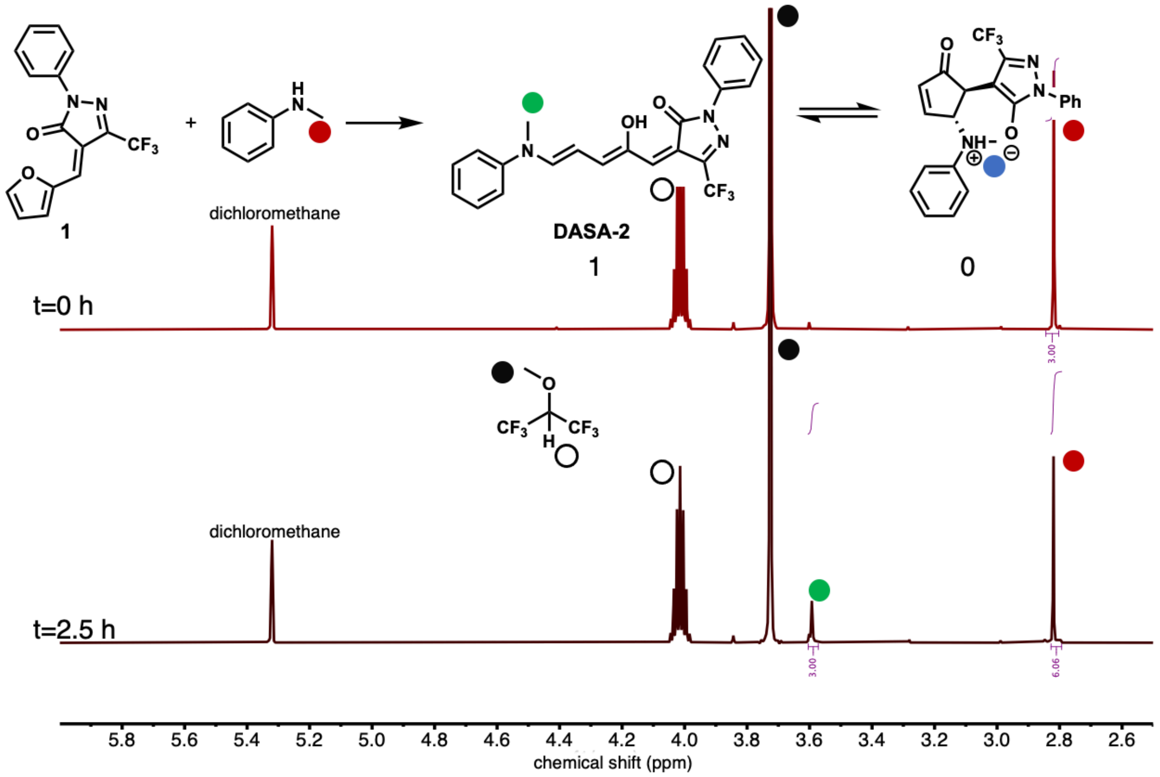

Figure S15: ^{1}H NMR (600 MHz, CD_2Cl_2) spectra from kinetic experiments for DASA-**2** in dichloromethane at t=0 and t= 2.5 h with 10 eq. of HFIPMe regarding **1**. No closed form can be observed.

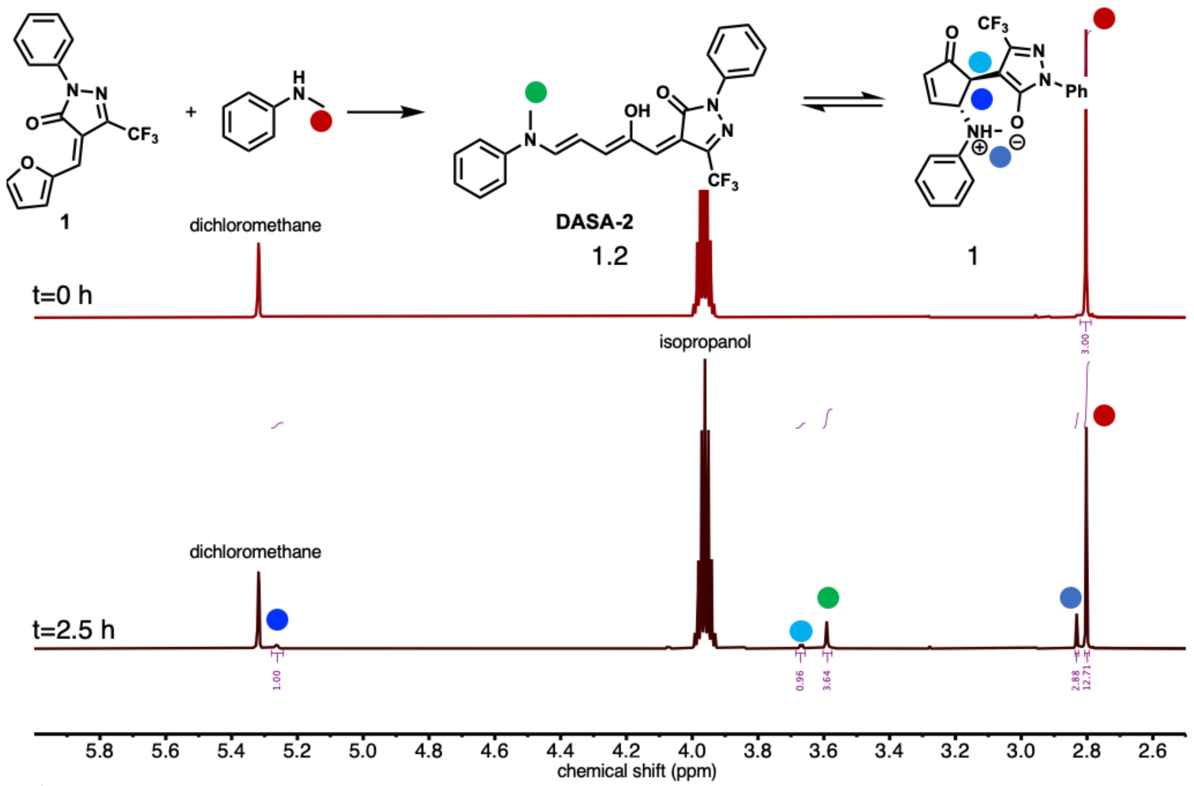

Figure S16: ^{1}H NMR (600 MHz, CD_2Cl_2) spectra from kinetic experiments for DASA-**2** in dichloromethane at t=0 and t= 2.5 h with 10 eq. of isopropanol regarding **1**. 45% closed form can be observed.

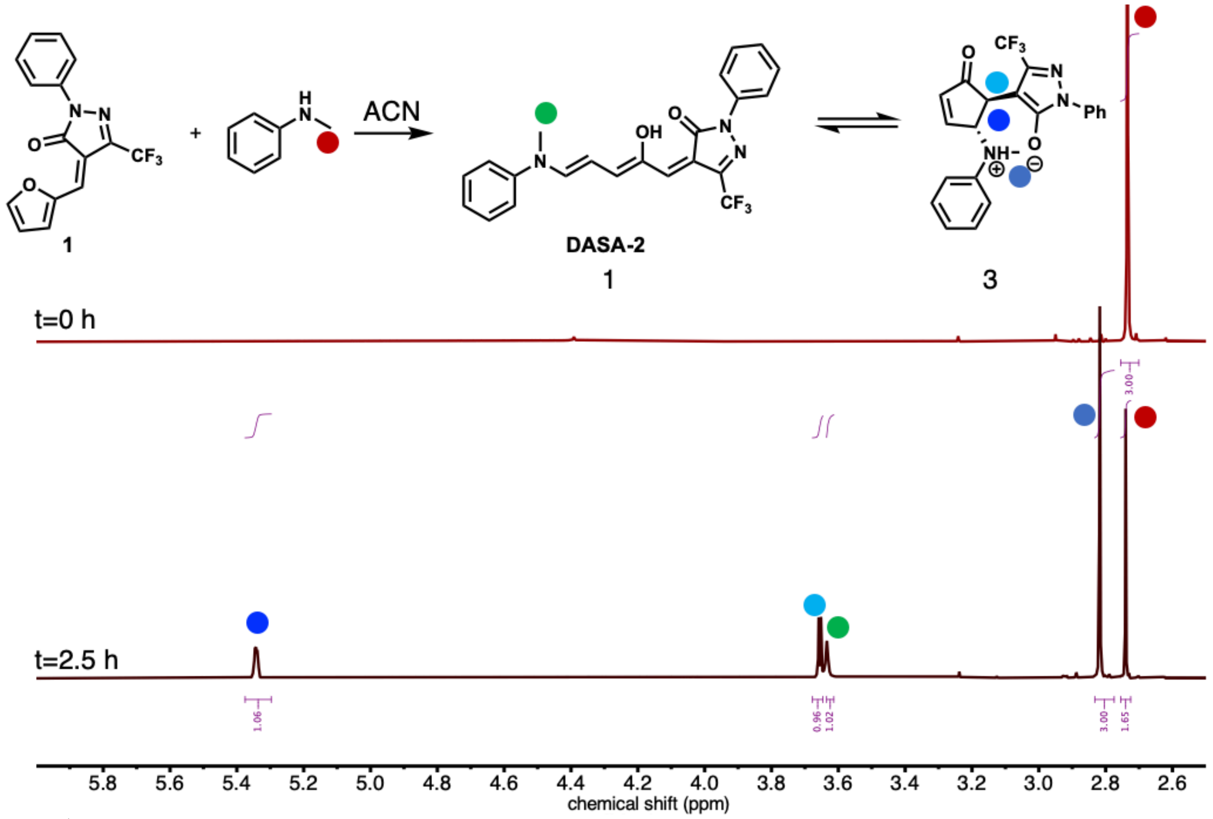

Figure S17: ^{1}H NMR (600 MHz, ACN-*d*$_3$) spectra from kinetic experiments for DASA-**2** in acetonitrile at t=0 and t= 2.5 h. 75% closed form can be observed.

5.2 Solvatochromic Shift Analysis

For **Figure 3a** DASA-**4**, DASA-**5**, DASA-**6** and DASA-**7** were measured via UV-Vis spectroscopy at a concentration of 10 µM in the following solvents: toluene ($E_{\mathrm{T}}^{\mathrm{N}}$: 0.099), diethyl ether ($E_{\mathrm{T}}^{\mathrm{N}}$: 0.117), tetrahydrofuran ($E_{\mathrm{T}}^{\mathrm{N}}$: 0.207), ethyl acetate ($E_{\mathrm{T}}^{\mathrm{N}}$: 0.228), chloroform ($E_{\mathrm{T}}^{\mathrm{N}}$: 0.259), dichoromethane ($E_{\mathrm{T}}^{\mathrm{N}}$: 0.309), acetone ($E_{\mathrm{T}}^{\mathrm{N}}$: 0.355), dimethyl sulfoxide ($E_{\mathrm{T}}^{\mathrm{N}}$: 0.444), acetonitrile ($E_{\mathrm{T}}^{\mathrm{N}}$: 0.46), methanol ($E_{\mathrm{T}}^{\mathrm{N}}$: 0.726), HFIP ($E_{\mathrm{T}}^{\mathrm{N}}$: 1.068).[13] Data for DASA-**5**, DASA-**6** and DASA-**7** was partially taken from literature.[5,10,14]

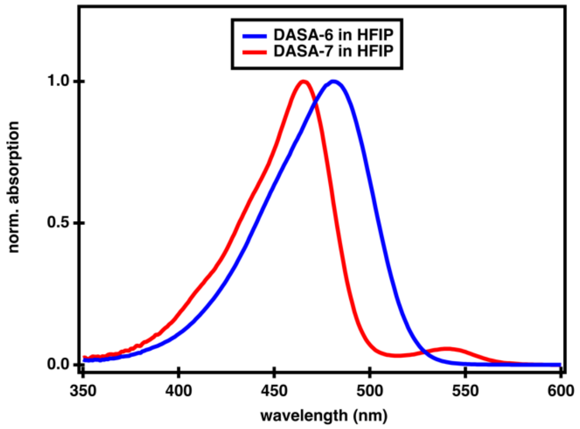

Figure S18: Normalized UV-Vis spectra of DASA-**6** and DASA-**7** in HFIP.

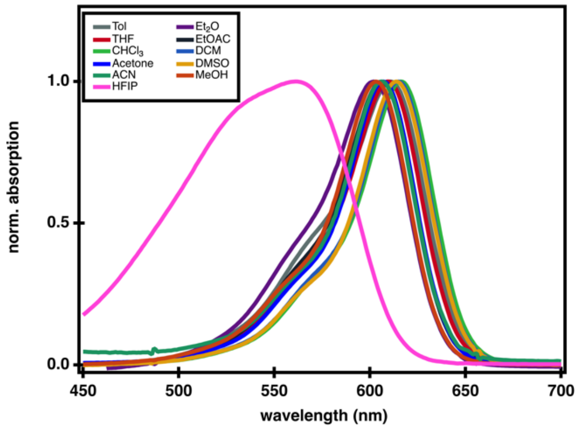

Figure S19: Normalized UV-Vis spectra of DASA-**4** in a range of solvents.

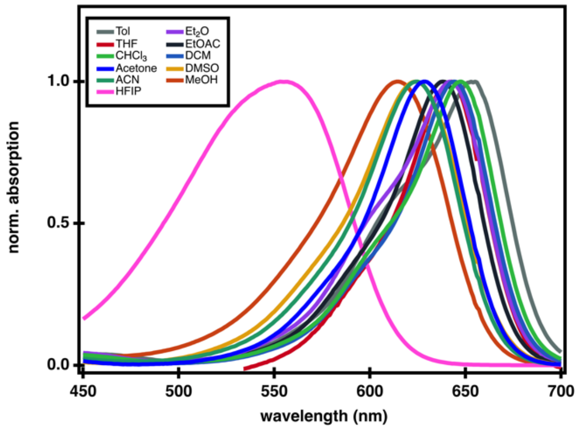

Figure S20: Normalized UV-Vis spectra of DASA-**5** in a range of solvents.

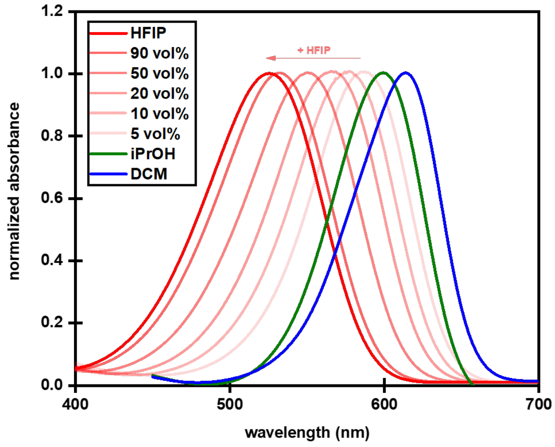

Figure S21: Normalized UV-Vis spectra of DASA-**2** in DCM/HFIP mixtures and in *i*PrOH.

5.3 Effect of HFIP on PTSS

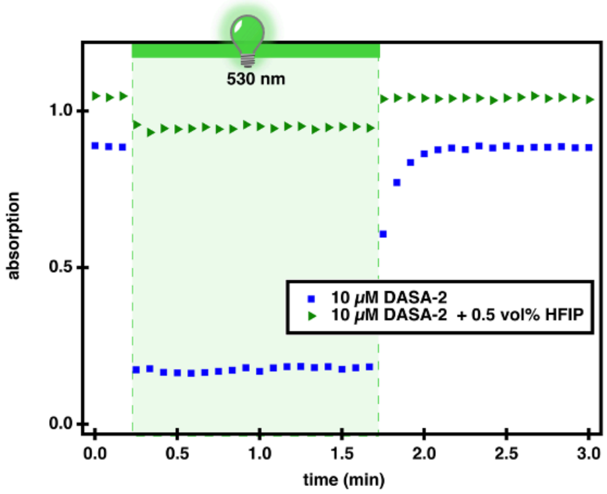

Figure S22: Time dependent UV-Vis spectroscopy to observe the photochromism of DASA-**2** in dichloromethane at 10 µM followed at λ_{max} (609 nm). Upon the addition of 0.5 vol% HFIP the photothermalstationary state (PTSS) decreases from 81% to 11% closed isomers.

6. UV-Vis Absorption Spectra

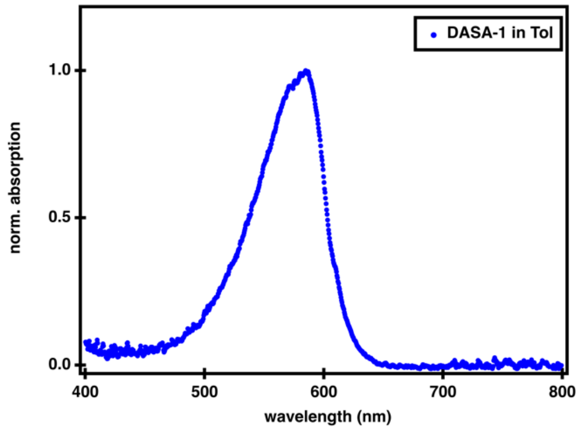

Figure S23: Normalized UV-Vis spectra of DASA-**1** in toluene. λ_{max}: 590 nm.

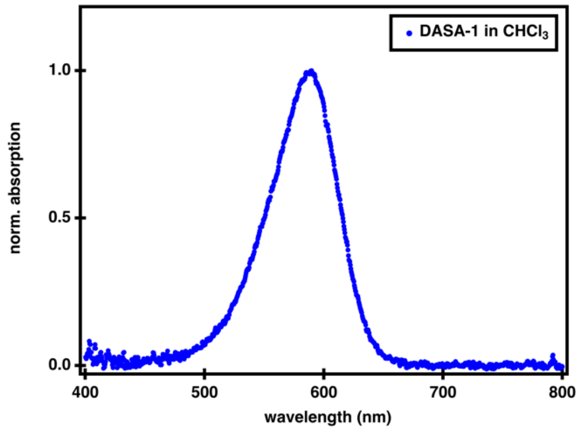

Figure S24: Normalized UV-Vis spectra of DASA-**1** in chloroform. λ_{max}: 588 nm.

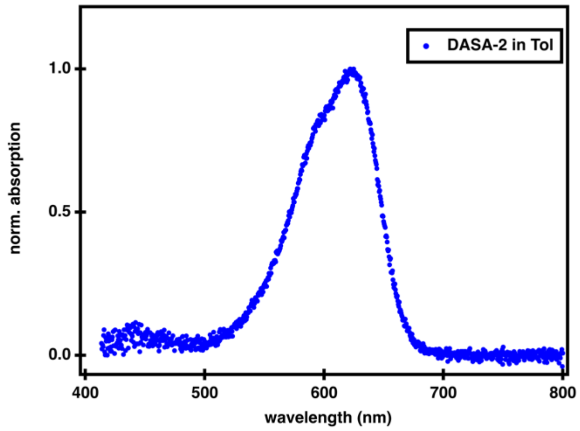

Figure S25: Normalized UV-Vis spectra of DASA-**2** in toluene. λ_{max}: 625 nm.

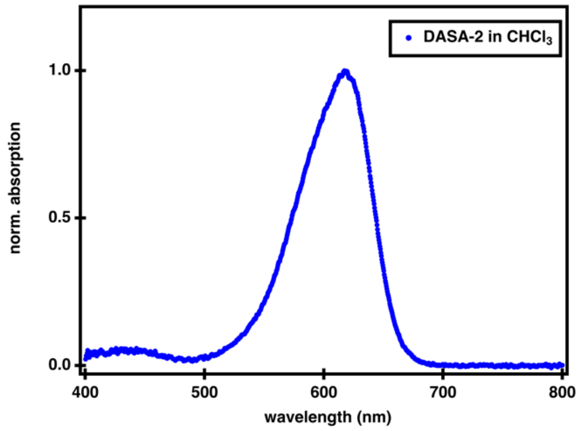

Figure S26: Normalized UV-Vis spectra of DASA-**2** in chloroform. λ_{max}: 617 nm.

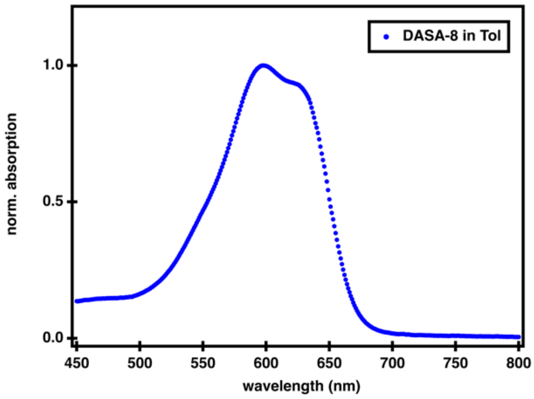

Figure S27: Normalized UV-Vis spectra of DASA-**8** in toluene. λ_{max}: 598/624 nm.

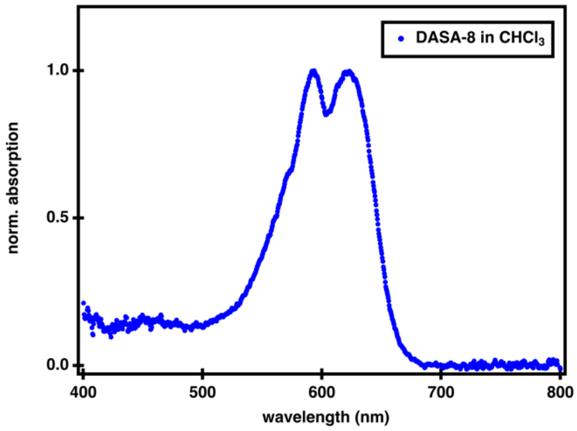

Figure S28: Normalized UV-Vis spectra of DASA-**8** in chloroform. λ_{max}: 594/621 nm.

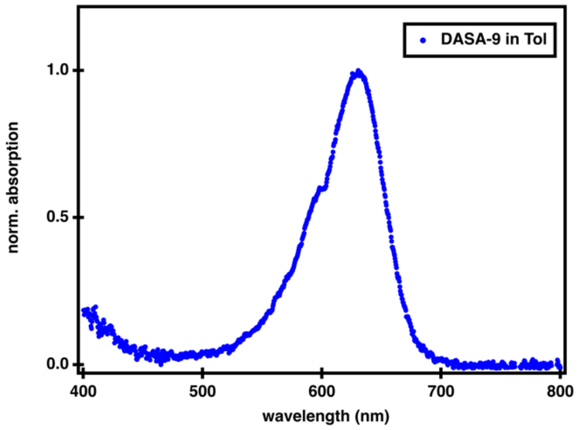

Figure S29: Normalized UV-Vis spectra of DASA-**9** in toluene. λ_{max}: 631 nm.

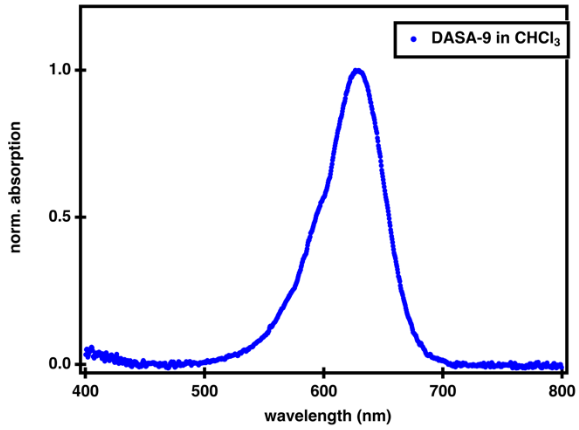

Figure S30: Normalized UV-Vis spectra of DASA-**9** in chloroform. λ_{max}: 625 nm.

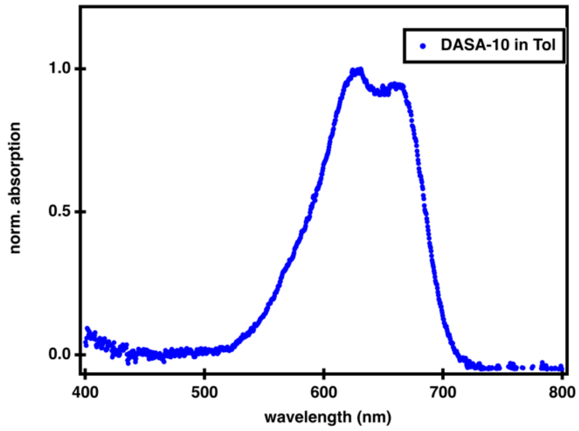

Figure S31: Normalized UV-Vis spectra of DASA-**10** in toluene. λ_{max}: 630/662 nm.

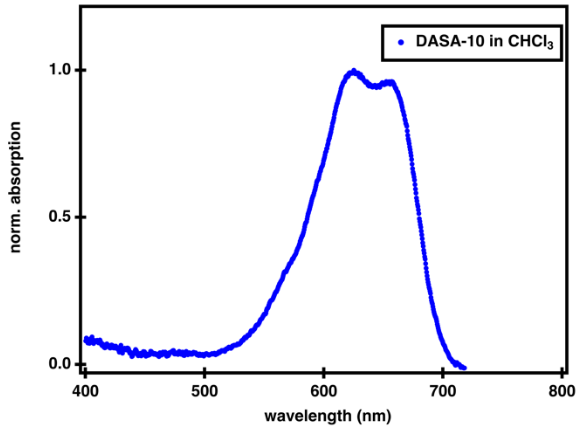

Figure S32: Normalized UV-Vis spectra of DASA-**10** in chloroform. λ_{max}: 625/656 nm.

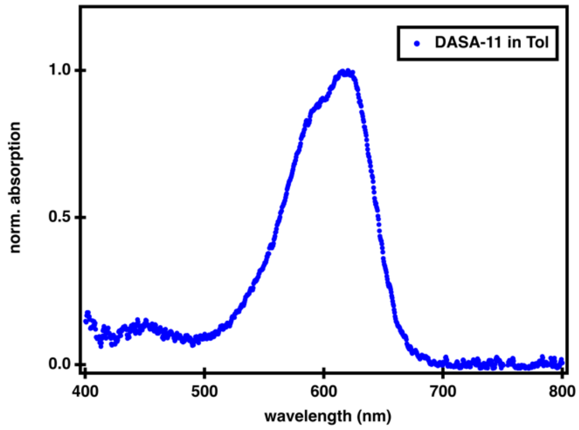

Figure S33: Normalized UV-Vis spectra of DASA-**11** in toluene. λ_{max}: 623 nm.

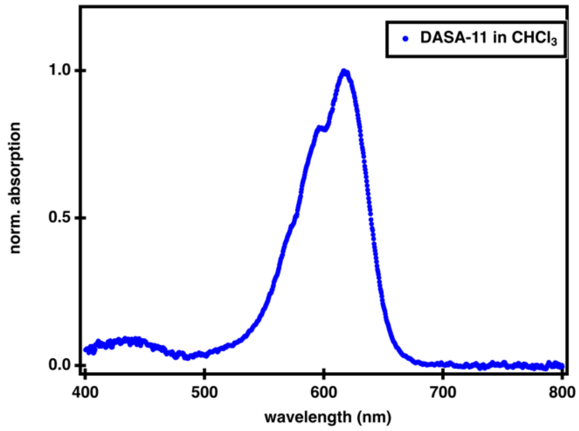

Figure S34: Normalized UV-Vis spectra of DASA-**11** in chloroform. λ_{max}: 616 nm.

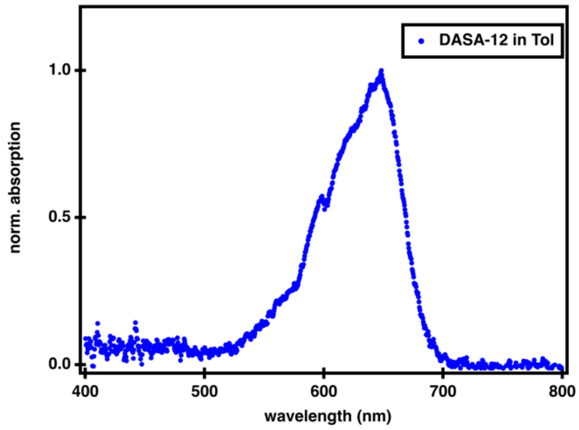

Figure S35: Normalized UV-Vis spectra of DASA-**12** in toluene. λ_{max}: 647 nm.

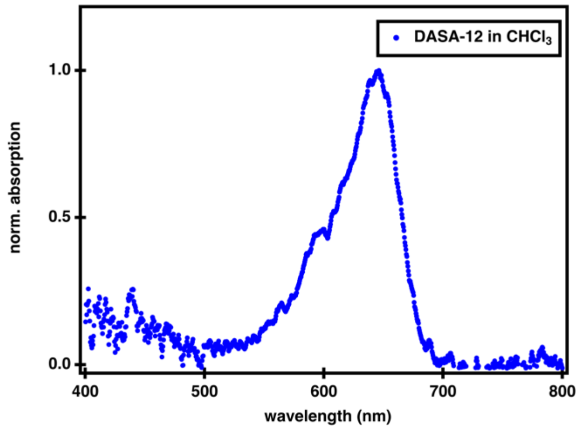

Figure S36: Normalized UV-Vis spectra of DASA-**12** in chloroform. λ_{max}: 646 nm.

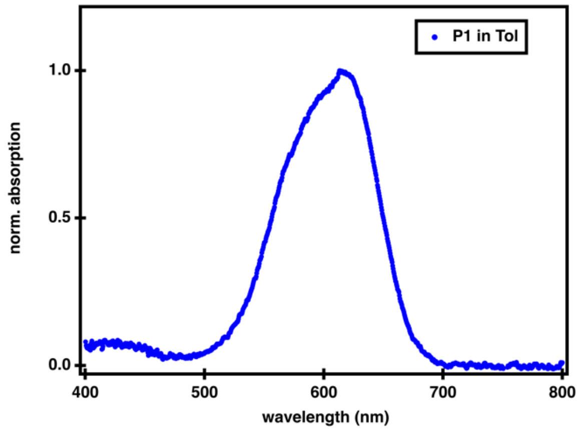

Figure S37: Normalized UV-Vis spectra of **P1** in toluene. λ_{max}: 613 nm.

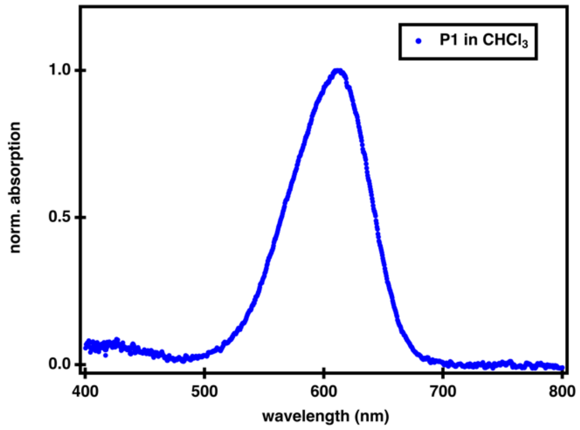

Figure S38: Normalized UV-Vis spectra of **P1** in chloroform. λ_{max}: 609 nm.

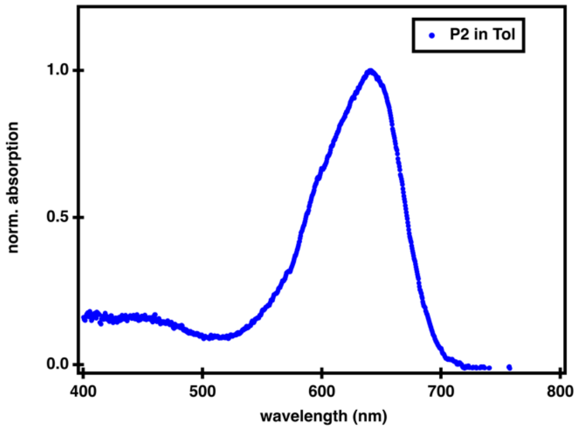

Figure S39: Normalized UV-Vis spectra of **P2** in toluene. λ_{max}: 641 nm.

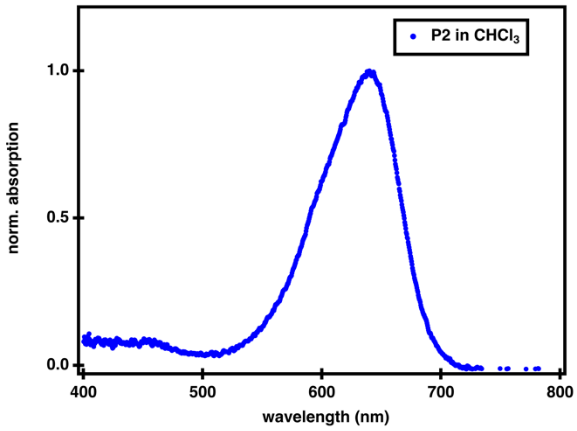

Figure S40: Normalized UV-Vis spectra of **P2** in chloroform. λ_{max}: 640 nm.

7. DFT Calculations

Density functional theory (DFT) calculations at B3LYP-GD3BJ/6-31G(d) level of theory were used for geometry optimizations of the open form DASAs to determine the dihedral angle between the acceptor and donor groups, as described before for second generation DASAs.[11] Calculations at B3LYP-GD3BJ/6-311++(d,p) level of theory were performed to predict the character of the frontier orbitals and the optical excitation energy. The calculations were performed with the Gaussian 16 Rev.A.03 software and solvent effects were considered using a conductor-like polarizable continuum model (CPCM, toluene). The calculated HOMO-LUMO gaps correlate well with the relative ordering of the experimentally observed absorption peaks, however, the absolute energies are systematically over-predicted by 0.4 eV (~ 100 nm blue-shifted λ_{max} relative to experimentally determined value in toluene), which is in accordance with previous findings for second generation DASAs.[11] The calculated HOMO-LUMO gaps listed in **Table S2** were adjusted by this amount. Time-dependent DFT methods and other functionals were also tested but the predictions of the ordering of the HOMO-LUMO gaps in the series were less consistent with experimental results. Dihedral angles ($\Phi_{D\text{-}A}$) between acceptor and donor groups were determined from the optimized geometries and frontier orbitals graphically represented by using the Avogadro software (**Figure S41**–**S42**).

Table S2. DFT predicted λ_{max} of the open form DASA compared to their actual λ_{max} in nm and dihedral angles between donor and acceptor groups.

DASA	Observed λ_{max} (nm)[a]	Calc. λ_{max} (nm)[b]	Deviation from experimental λ_{max} (nm)	Dihedral angles ΦD-A [c]	
				R on OH side	R opposite OH side
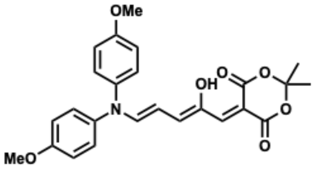 **DASA-1**	590	599	+9	57.6°	37.6°
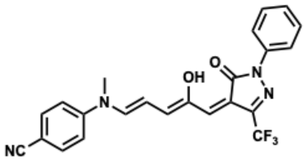 **DASA-8**	598/624[c]	622	–2	-	35.4°
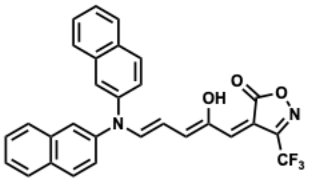 **DASA-9**	631	633	+2	55.9°	37.5°
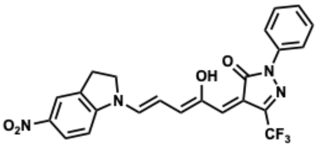 **DASA-10**	630/662[c]	664	+2	0.1°	0°
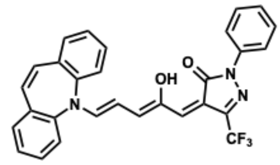 **DASA-11**	623	611	–12	61.8°	59.3°

[a] Measured in toluene at concentrations of approx. 10–20 µM. [b] The energy values were systematically over predicted by 0.4 eV and all the listed values are corrected by this value. [c] Splitted absorbance maxima were observed. See *section 6* for UV-Vis spectra.

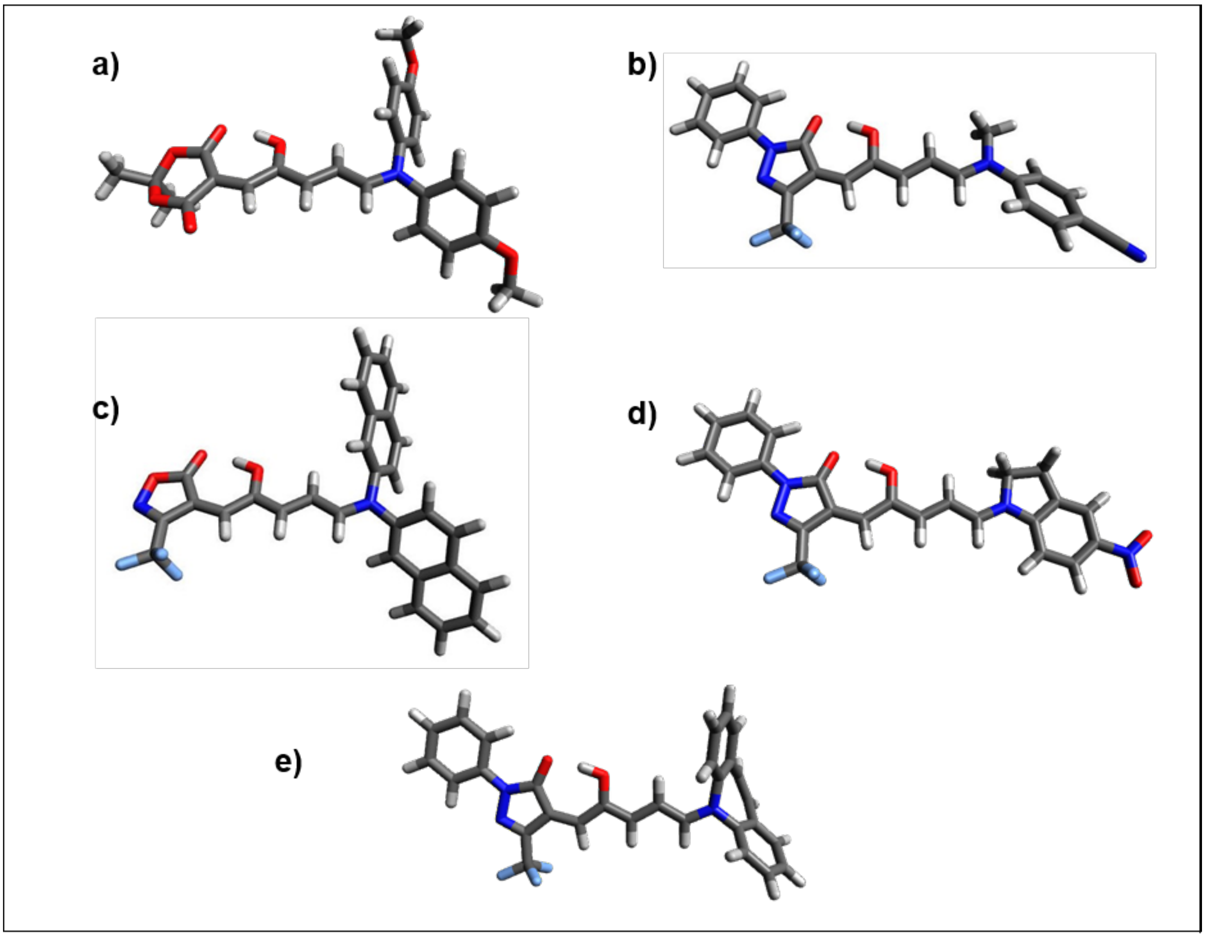

Figure S41: Geometry optimized structures of the open form of a) DASA-**1**, b) DASA-**8**, c) DASA-**9**, d) DASA-**10** and e) DASA-**11** calculated with DFT.

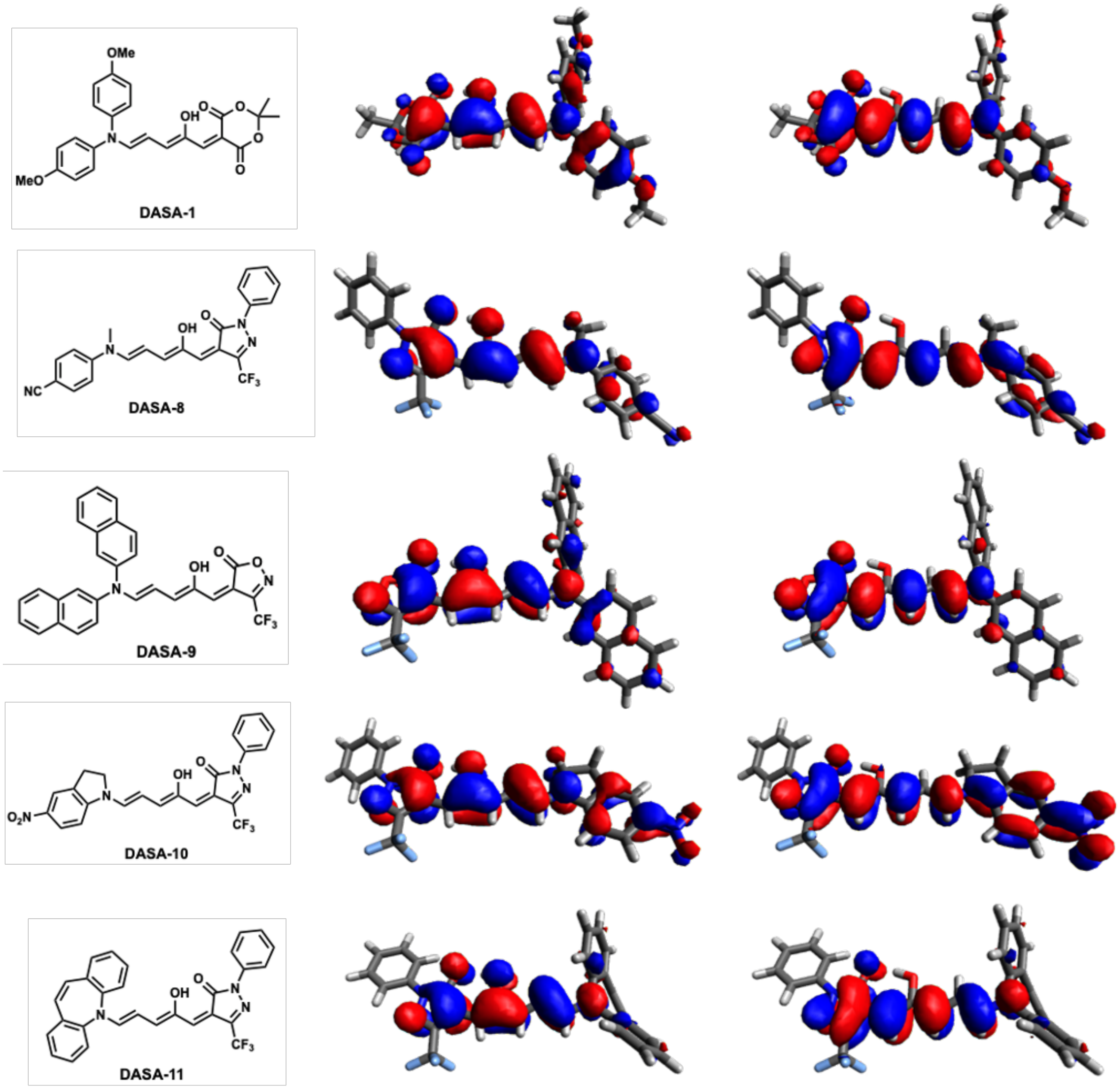

Figure S42: DFT predicted frontier orbitals of the open form. HOMOs are on the left, LUMOs on the right.

8. Photoswitching Experiments

The photoinduced optical absorption kinetics were measured on a pump-probe setup. The pump beam was generated by a light emitting diode (LED) source (Thorlabs) coupled into a multimode optical fiber terminated with an output collimator. The LED intensity was controlled through a digital-to-analog converter (National Instruments USB-6009) using LabVIEW. The probe beam was produced by High Power MINI Deuterium Tungsten Halogen Source w/shutter 200–2000 nm (Ocean Optics DH-MINI) coupled into a multimode fiber with an output collimator for the light delivery. The probe light was modulated by a shutter (Uniblitz CS25) which could be controlled manually or through a digital output port (National Instruments USB-6009) using LabVIEW. Pump and probe beams were overlapped using steering and focusing optics at a 90° angle inside a sample holder, which allowed for a 10x10 mm rectangular spectrophotometer cells that was connected to a circulating bath for temperature control. Additionally, the solutions were stirred during the measurements by a miniature stirring plate inserted into the sample holder (Starna Cells SCS 1.11). The sample holder was placed into a metal enclosure to prevent exposure to ambient light. Both pump and probe beams were nearly collimated inside the cell with a diameter of about 2 mm. The pump beam was blocked after passing through the sample and the probe beam was directed by a system of lenses into the detector (Ocean Optics Flame-S1-XR spectrometer), which acquired spectra of the probe light. The detector was connected to a PC via USB port. The experiment was controlled by a National Instrument LabVIEW program which collected the probe light spectra, determined sample optical absorption spectra, controlled pump and probe light sources, and stored the data on the computer S3 hard

drive according to the experimental protocol. Experiments were performed in at 10 μM concentration unless otherwise stated. Samples were left to equilibrate overnight prior to measurements unless otherwise stated.

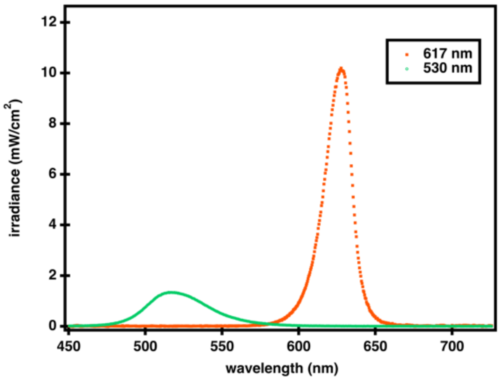

Figure S43: irradiance of Thorlabs 530 nm and 617 nm LED used in experiments. Total irradiance for 530 nm: 69.7 mW/cm^2; 617 nm: 240.2 mW/cm^2. Measured using an Ocean Optics hand-held spectrometer with cosine corrector and radiometric calibration (model USB 2000).

8.1 Overview Switching Parameters

Table S3. Photoswitching properties for novel DASAs of this report.

DASA	Solvent	λ_{max} (nm)	thermal equilibrium (% open form at eq.)	thermal recovery	half-life $t_{½}$ (s^{-1})	PTSS (% closed under irradiation)
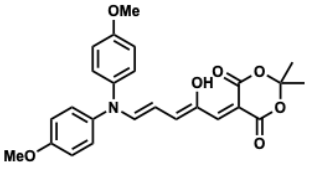 DASA-1	Toluene	590	19%	-	-	100%
	Chloroform	588	32%	100%	3558	100%
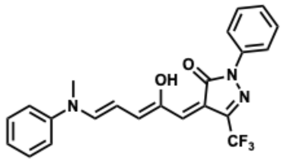 DASA-2	Toluene	625	84%	100%	94	100%
	Chloroform	617	87%	100%	13	93%
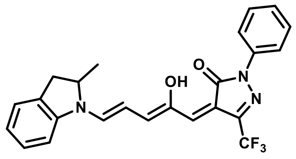 DASA-5	Chloroform	645[a]	>95%[a]	100%[a]	5[a]	94%[a]
	Toluene	655[a]	100%[a]	88%[a]	40[a]	100%[a]
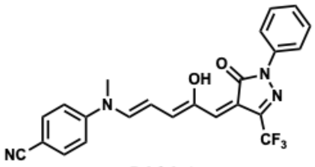 DASA-8	Toluene	598/ 624[b]	insoluble	-	-	-
	Chloroform	594/ 621[b]	<5%	100%	973	n.d.[c]
	Toluene	631	83%	100%	868	100%

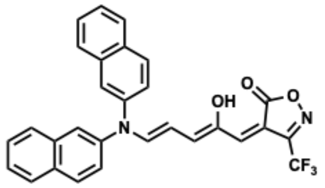 DASA-9	Chloroform	625	>95%	100%	67	88%
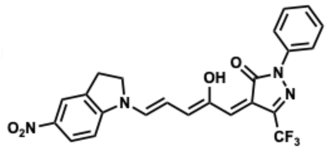 DASA-10	Toluene	630/ 662 [b]	insoluble	n.d.[d]	-[d]	100%
	Chloroform	625/ 656 [b]	<5%[e]	-	-	100%
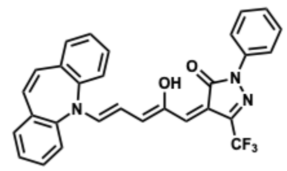 DASA-11	Toluene	623	76%	100%	1594	100%
	Chloroform	616	89%	100%	202	15%
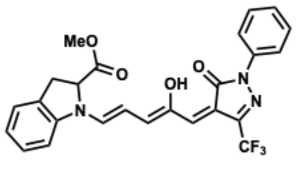 DASA-12	Toluene	647	29%	n.d. [d]	17498	100%
	Chloroform	646	9%	100%	3234[g]	100%
**P1**	Toluene	613	n.d.[f]	100%	23	40%
	Chloroform	609	n.d.[f]	100%	9	29%
**P2**	Toluene	641	n.d.[f]	100%	53	85%
	Chloroform	640	n.d.[f]	100%	24	73%

All compounds were also tested in acetonitrile, but did not show any absorbance after equilibration at 10 µM. [a] Data taken from Hemmer et al.[5] [b] Two absorbance maxima were observed. See *section 6 for c*omplete UV-Vis. [c] Conversion was not completed after 100 s of irradiation [d] Recovery was not completed after 22 h. [e] Limited solubility. [f] Equilibrium DASA signals not clearly observable by NMR spectroscopy for DASA polymers. [g] 1545 s at 100 µM.

8.2 Time Dependent UV-Vis Absorption Spectra

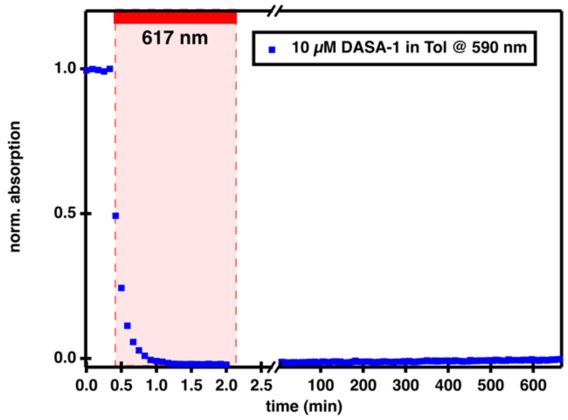

Figure S44: Time dependent UV-Vis spectroscopy to observe the photochromism of DASA-**1** in toluene at 10 μM (initial absorbance: 0.35) followed at λ_{max} (590 nm). Quantitative conversion of the open form to the closed form under light irradiation with 617 nm for 100 seconds. No subsequent thermal recovery in the dark can be observed.

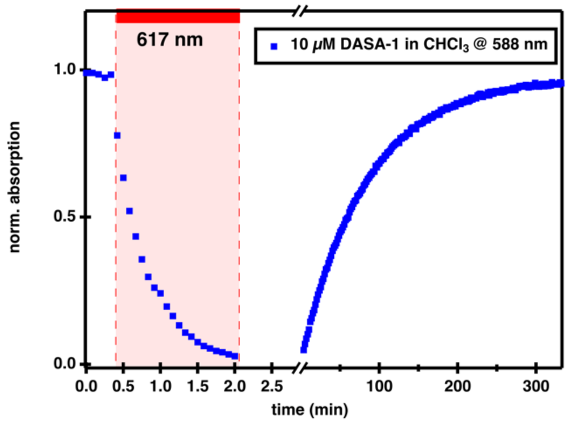

Figure S45: Time dependent UV-Vis spectroscopy to observe the photochromism of DASA-**1** in chloroform at 10 μM (initial absorbance: 0.41) followed at λ_{max} (588 nm). Quantitative conversion of the open form to the closed form under light irradiation with 617 nm for 100 seconds and subsequent thermal recovery in the dark can be observed.

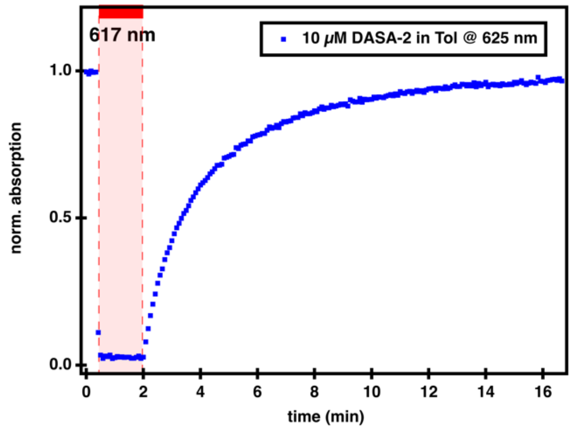

Figure S46: Time dependent UV-Vis spectroscopy to observe the photochromism of DASA-**2** in toluene at 10 μM (initial absorbance: 0.4) followed at λ_{max} (625 nm). Quantitative conversion of the open form to the closed form under light irradiation with 617 nm for 100 seconds and subsequent thermal recovery in the dark can be observed.

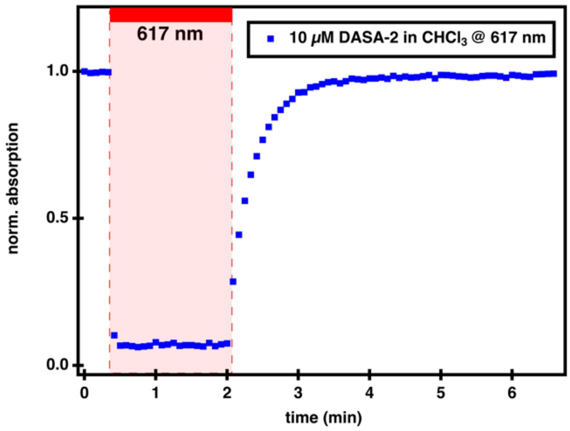

Figure S47: Time dependent UV-Vis spectroscopy to observe the photochromism of DASA-**2** in chloroform at 10 μM (initial absorbance: 1.1) followed at λ_{max} (617 nm). Quantitative conversion of the open form to the closed form under light irradiation with 617 nm for 100 seconds and subsequent thermal recovery in the dark can be observed.

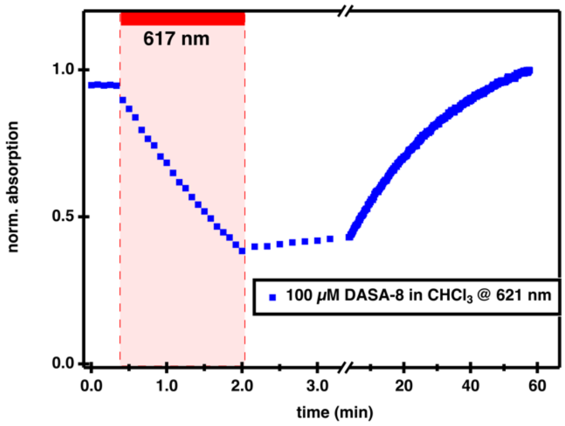

Figure S48: Time dependent UV-Vis spectroscopy to observe the photochromism of DASA-**8** in chloroform at 100 μM (initial absorbance: 0.35) followed at λ_{max} (619 nm). Conversion of 60% of the open form to the closed form under light irradiation with 617 nm for 100 seconds and subsequent thermal recovery in the dark can be observed.

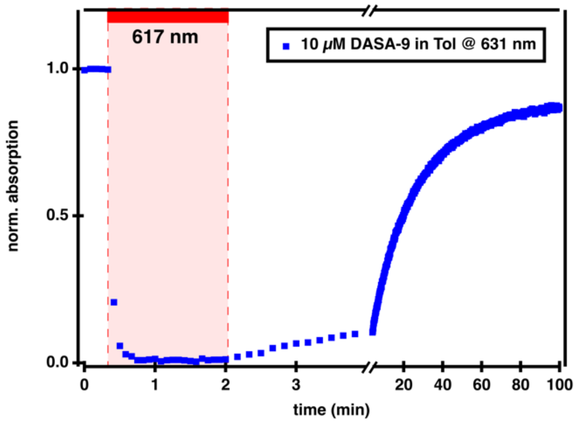

Figure S49: Time dependent UV-Vis spectroscopy to observe the photochromism of DASA-**9** in toluene at 10 μM (initial absorbance: 0.32) followed at λ_{max} (631 nm). Quantitative conversion of the open form to the closed form under light irradiation with 617 nm for 100 seconds and subsequent thermal recovery in the dark can be observed.

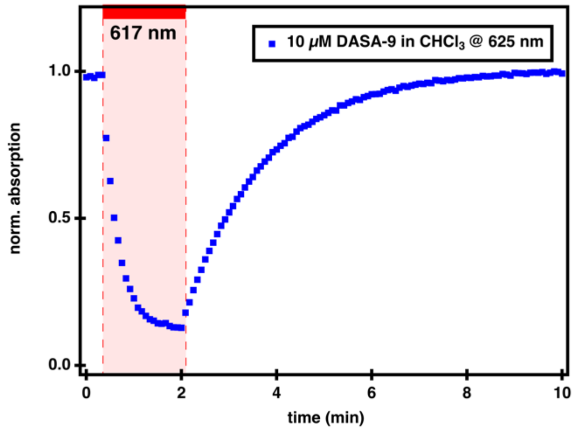

Figure S50: Time dependent UV-Vis spectroscopy to observe the photochromism of DASA-**9** in chloroform at 10 μM (initial absorbance: 0.56) followed at λ_{max} (625 nm). Conversion of 87% the open form to the closed form under light irradiation with 617 nm for 100 seconds and subsequent thermal recovery in the dark can be observed.

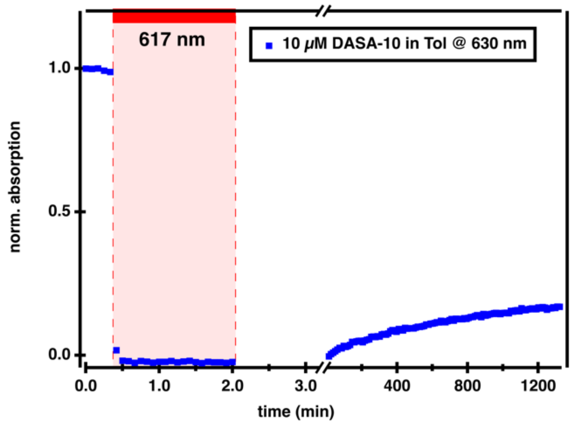

Figure S51: Time dependent UV-Vis spectroscopy to observe the photochromism of DASA-**10** in toluene at 10 μM (initial absorbance: 0.41) followed at λ_{max} (630 nm). Quantitative conversion of the open form to the closed form under light irradiation with 617 nm for 100 seconds and minimal subsequent thermal recovery in the dark can be observed.

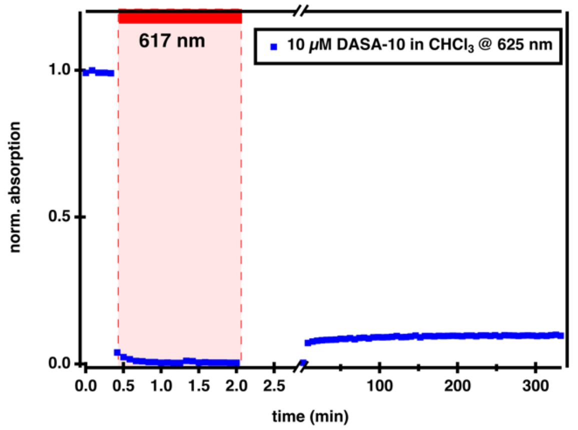

Figure S52: Time dependent UV-Vis spectroscopy to observe the photochromism of DASA-**10** in chloroform at 10 μM (initial absorbance: 0.41) followed at λ_{max} (625 nm). Quantitative conversion of the open form to the closed form under light irradiation with 617 nm for 100 seconds and minimal subsequent thermal recovery in the dark can be observed.

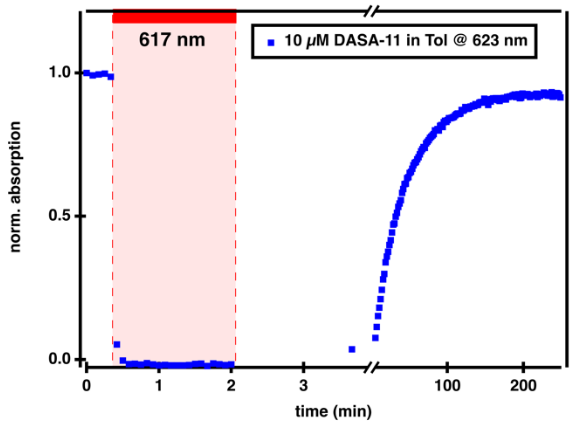

Figure S53: Time dependent UV-Vis spectroscopy to observe the photochromism of DASA-**11** in toluene at 10 μM (initial absorbance: 0.31) followed at λ_{max} (623 nm). Quantitative conversion of the open form to the closed form under light irradiation with 617 nm for 100 seconds and subsequent thermal recovery in the dark can be observed.

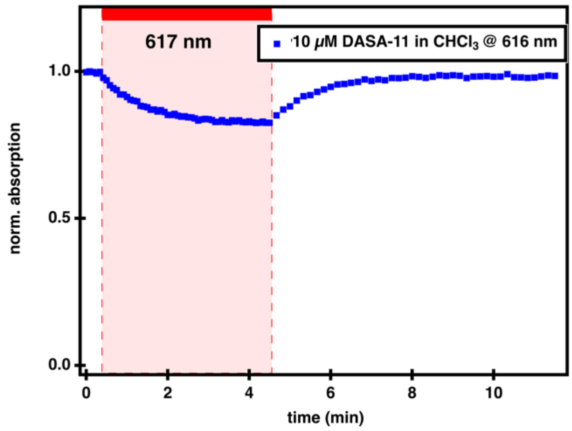

Figure S54: Time dependent UV-Vis spectroscopy to observe the photochromism of DASA-**11** in chloroform at 10 μM (initial absorbance: 0.56) followed at λ_{max} (616 nm). Conversion of 18% of the open form to the closed form under light irradiation with 617 nm for 100 seconds and subsequent thermal recovery in the dark can be observed.

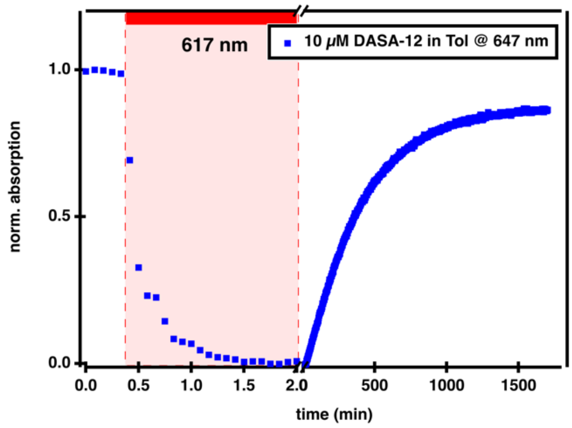

Figure S55: Time dependent UV-Vis spectroscopy to observe the photochromism of DASA-**12** in toluene at 10 μM (initial absorbance: 0.43) followed at λ_{max} (647 nm). Quantitative conversion of the open form to the closed form under light irradiation with 617 nm for 100 seconds and subsequent thermal recovery in the dark can be observed.

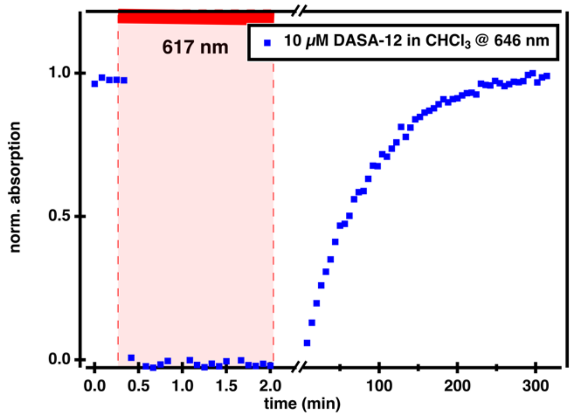

Figure S56: Time dependent UV-Vis spectroscopy to observe the photochromism of DASA-**12** in chloroform at 10 μM (initial absorbance: 0.05) followed at λ_{max} (646 nm). Quantitative conversion of the open form to the closed form under light irradiation with 617 nm for 100 seconds and subsequent thermal recovery in the dark can be observed.

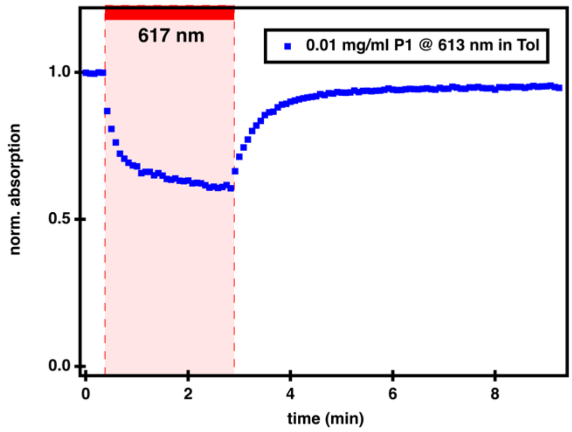

Figure S57: Time dependent UV-Vis spectroscopy to observe the photochromism of **P1** in toluene at 0.01 mg/mL (initial absorbance: 0.76) followed at λ_{max} (613 nm). Conversion of 40% of the open form to the closed form under light irradiation with 617 nm for 150 seconds and subsequent thermal recovery in the dark can be observed.

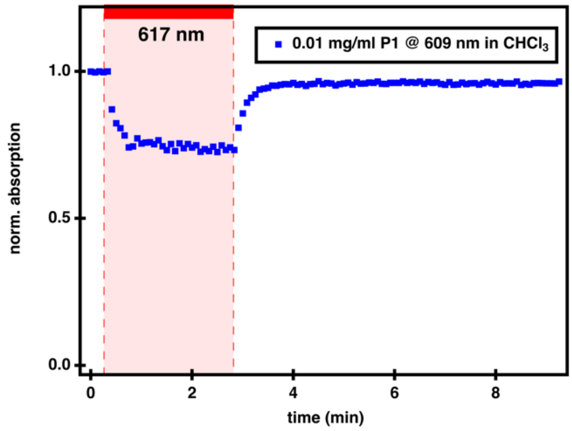

Figure S58: Time dependent UV-Vis spectroscopy to observe the photochromism of **P1** in chloroform at 0.01 mg/mL (initial absorbance: 0.73) followed at λ_{max} (609 nm). Conversion of 29% of the open form to the closed form under light irradiation with 617 nm for 150 seconds and subsequent thermal recovery in the dark can be observed.

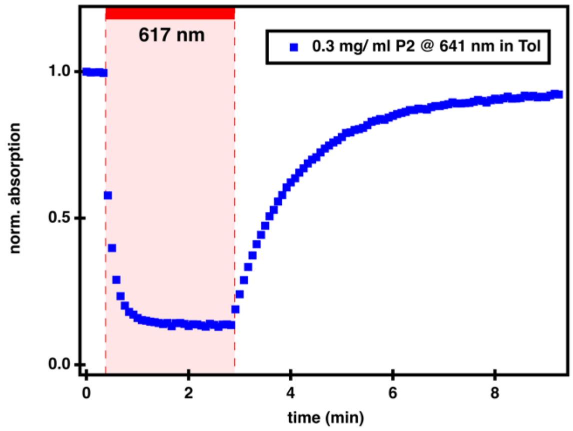

Figure S59: Time dependent UV-Vis spectroscopy to observe the photochromism of **P2** in toluene at 0.3 mg/mL (initial absorbance: 0.76) followed at λ_{max} (641 nm). Conversion of 85% of the open form to the closed form under light irradiation with 617 nm for 150 seconds and subsequent thermal recovery in the dark can be observed.

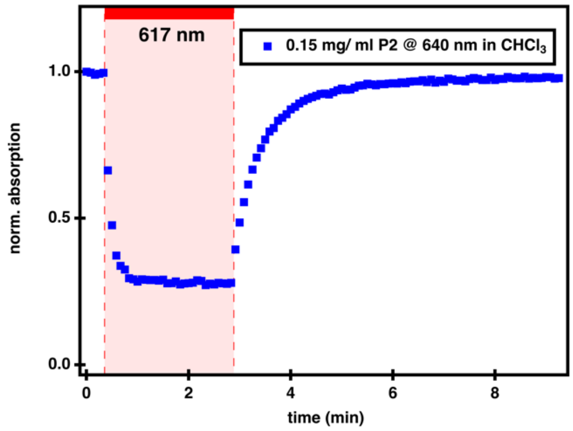

Figure S60: Time dependent UV-Vis spectroscopy to observe the photochromism of **P2** in chloroform at 0.15 mg/mL (initial absorbance: 0.88) followed at λ_{max} (640 nm). Conversion of 72% of the open form to the closed form under light irradiation with 617 nm for 150 seconds and subsequent thermal recovery in the dark can be observed.

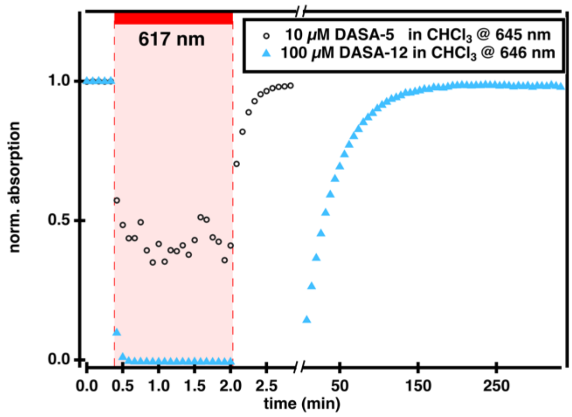

Figure S61: Time dependent UV-Vis spectroscopy to observe the photochromism of DASA-**5** and DASA-**12** in chloroform at 10 µM and 100 µM (initial absorbance: 0.9 and 1.0) followed at λ_{max} (645 nm and 646 nm). Quantitative conversion of the open form to the closed form under light irradiation with 617 nm for 100 seconds and subsequent thermal recovery in the dark can be observed. The half-life of the closed isomer of DASA-**12** is 1545 s at 100 µM. This difference from the value of 10 µM is due to the concentration dependence of DASA.

8.3 Thermodynamic Equilibrium NMR Spectroscopy

Samples were stored in the dark at room temperature overnight. Closed and open isomer were identified by ^{1}H NMR spectroscopy (**Figures S62–S73**).

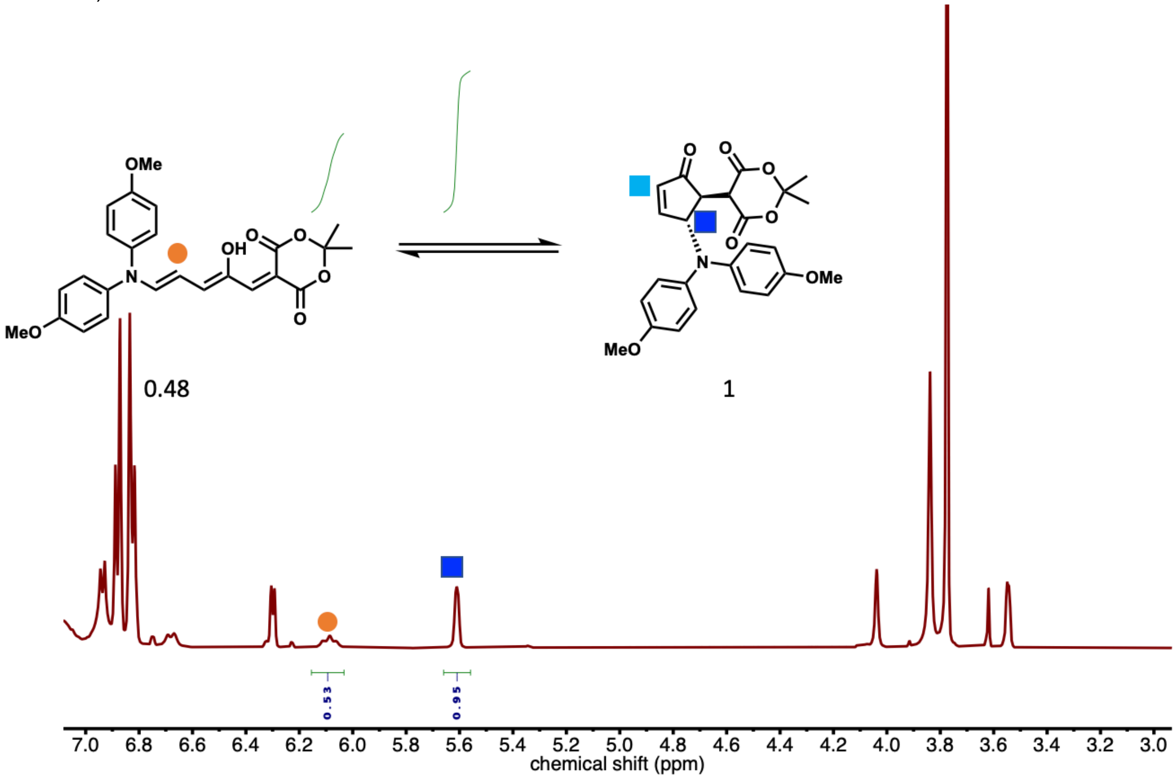

Figure S62: ^{1}H NMR (600 MHz, $CDCl_3$) spectra of the equilibrated DASA-**1**.

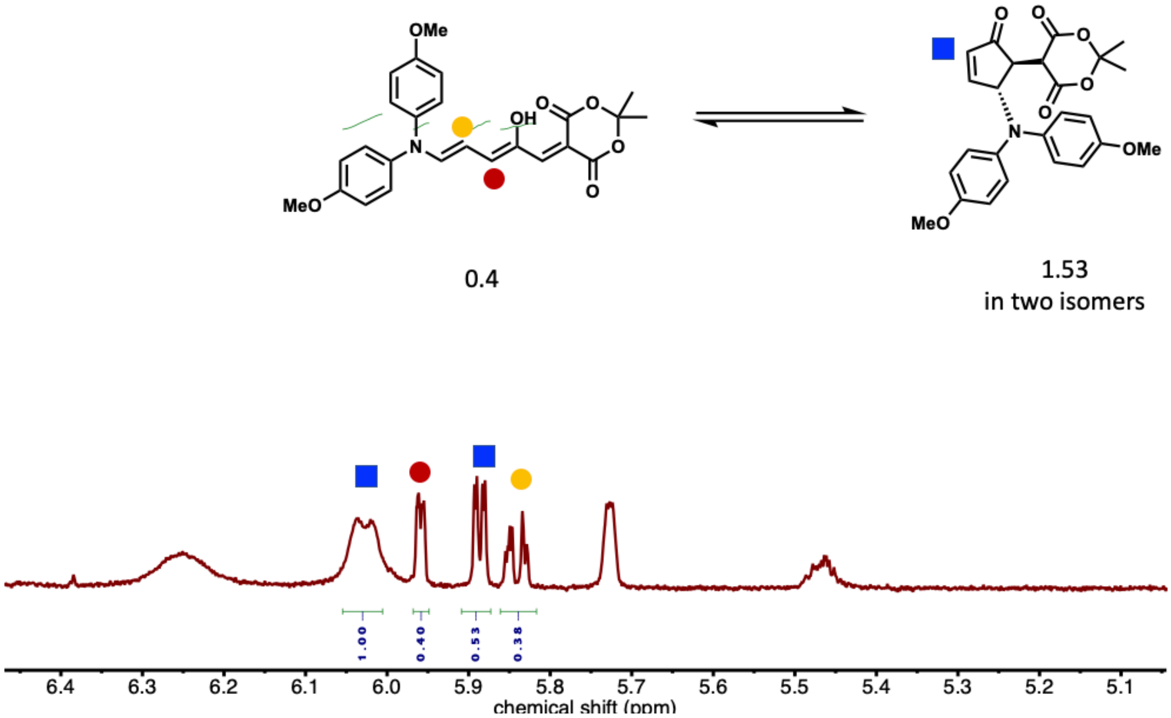

Figure S63: ^{1}H NMR (600 MHz, Tol-d_8) spectra of the equilibrated DASA-**1**.

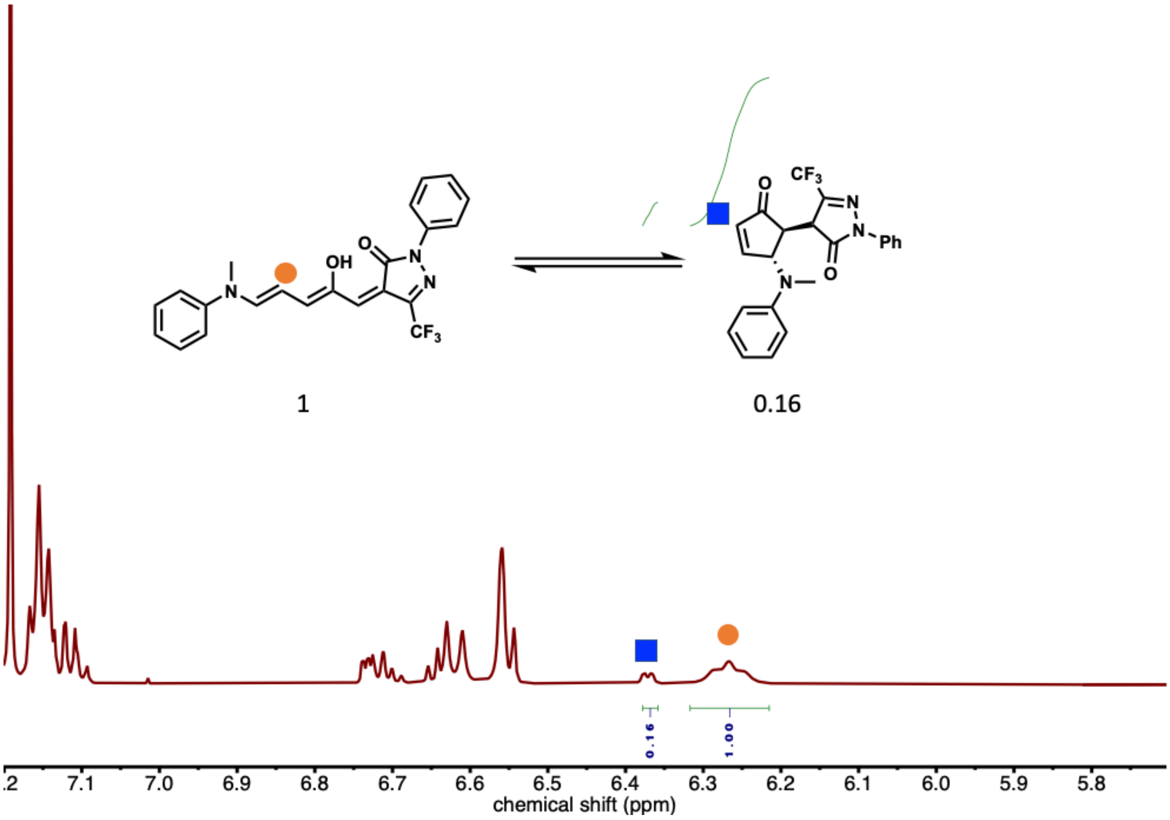

Figure S64: ^{1}H NMR (600 MHz, $CDCl_3$) spectra of the equilibrated DASA-**2**.

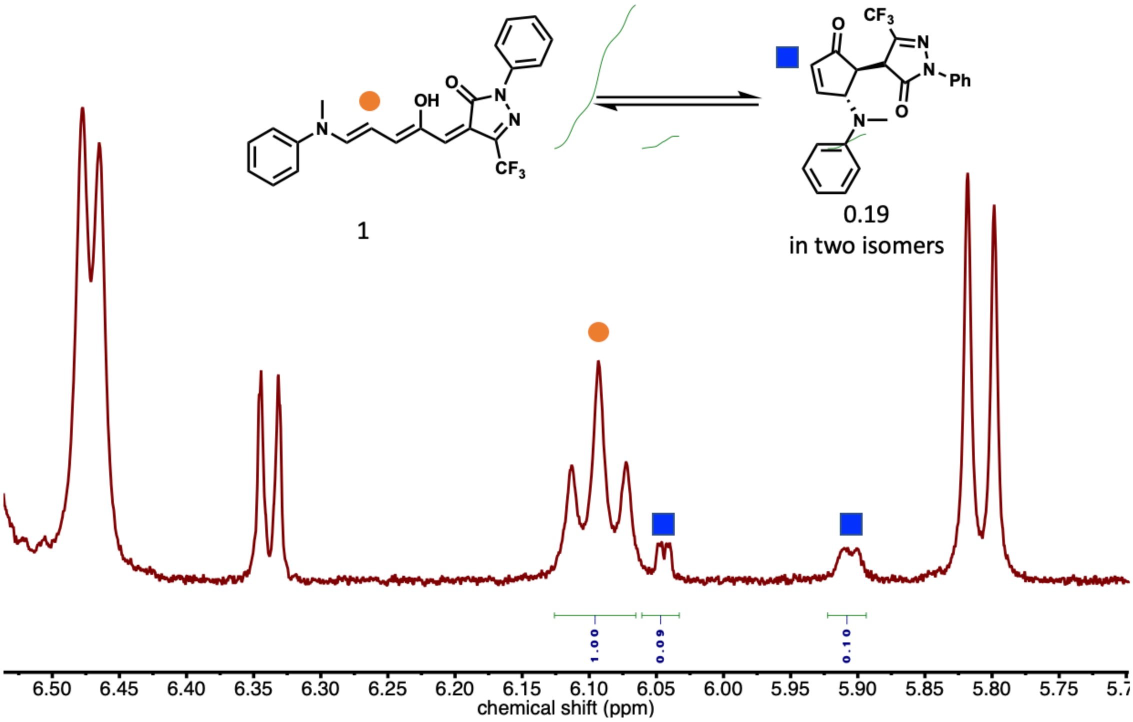

Figure S65: ^{1}H NMR (600 MHz, Tol-d_8) spectra of the equilibrated DASA-**2**.

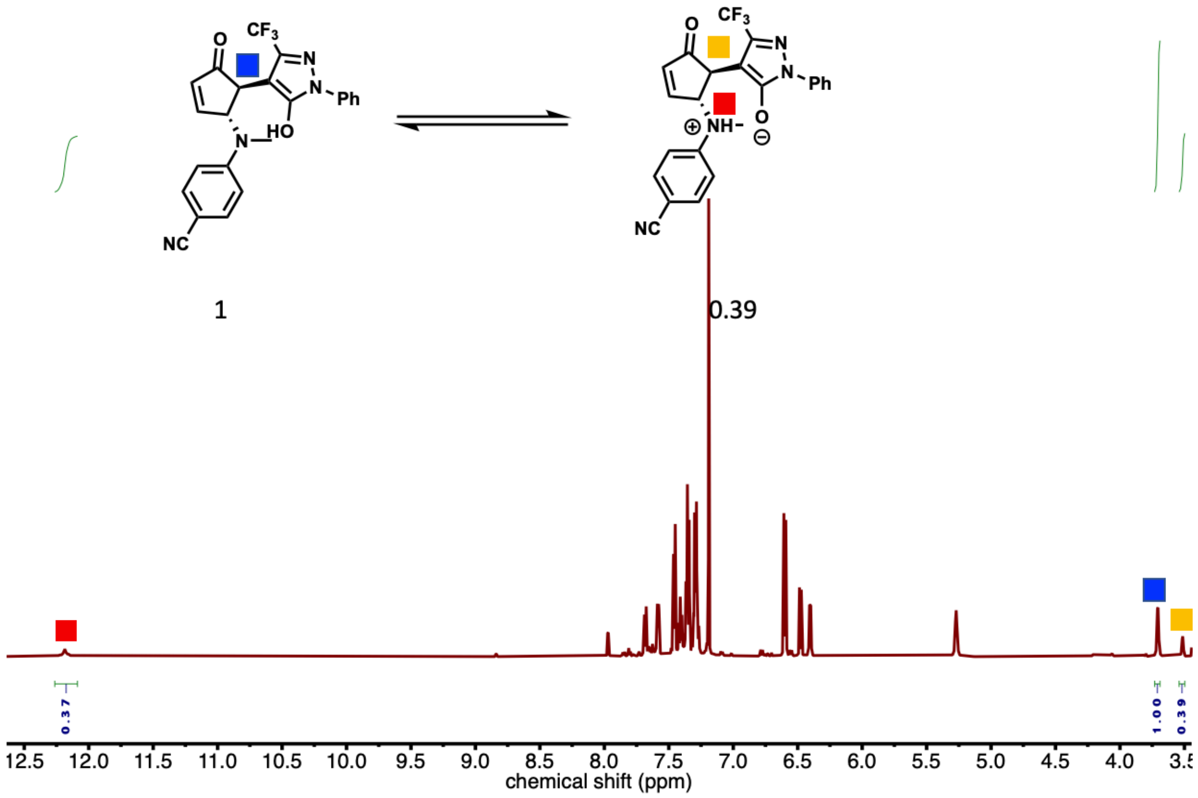

Figure S66: ^{1}H NMR (600 MHz, $CDCl_3$) spectra of the equilibrated DASA-**8**. No equilibration to the open form can be observed, but a zwitterionic closed isomer is formed over time.

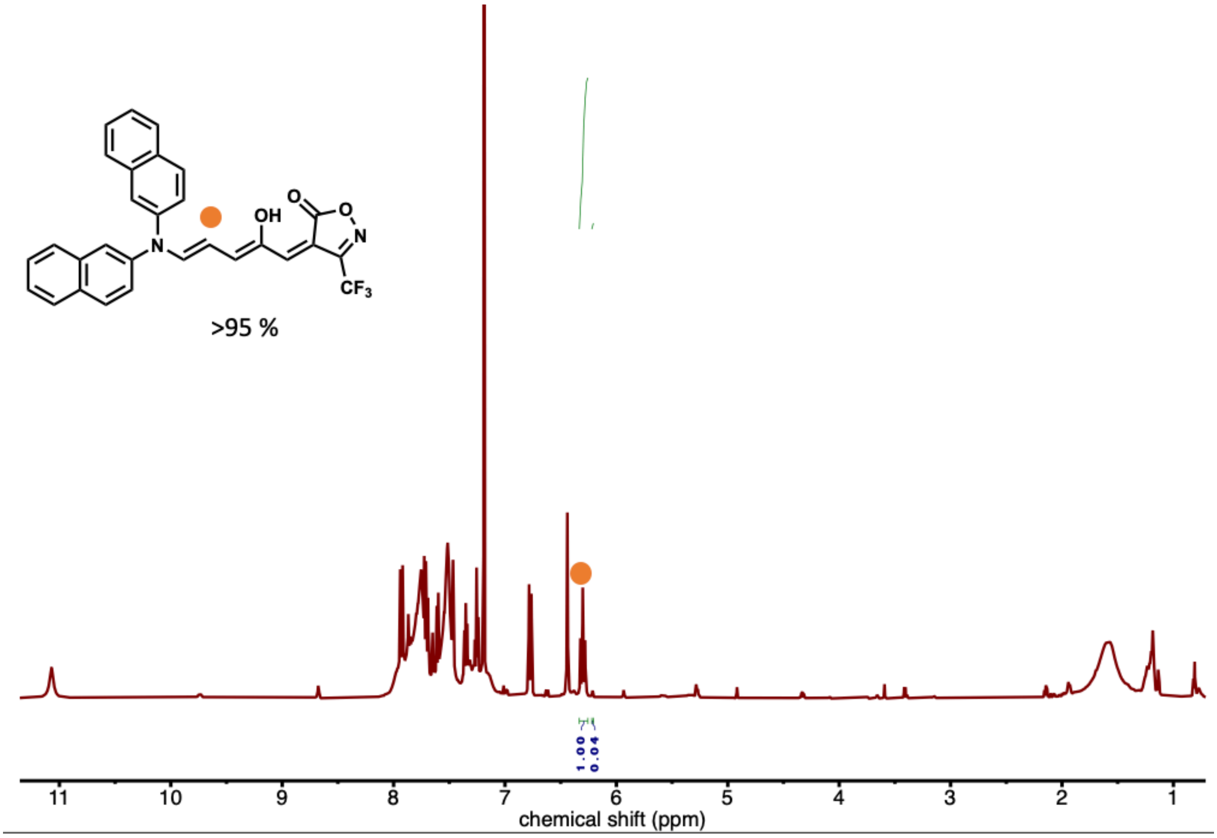

Figure S67: ^{1}H NMR (600 MHz, $CDCl_3$) spectra of the equilibrated DASA-**9**. No closed form can be observed.

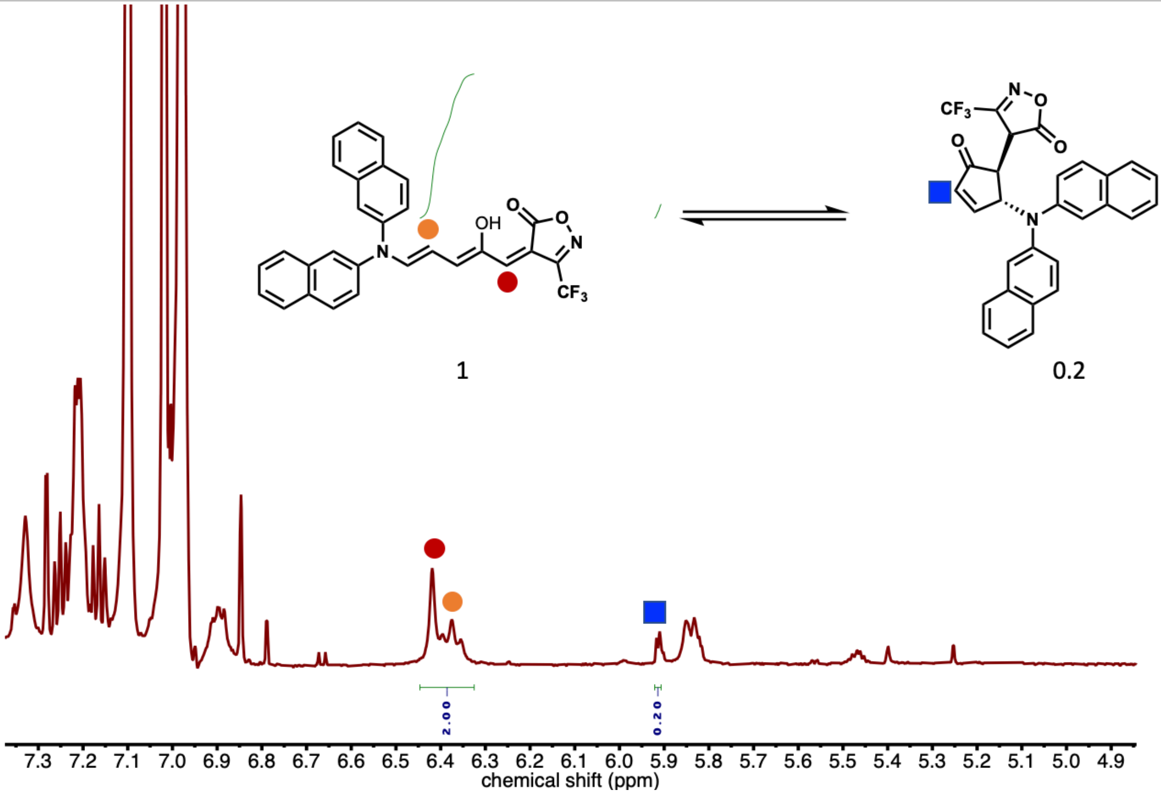

Figure S68: ^{1}H NMR (600 MHz, Tol-d_8) spectra of the equilibrated DASA-**9**.

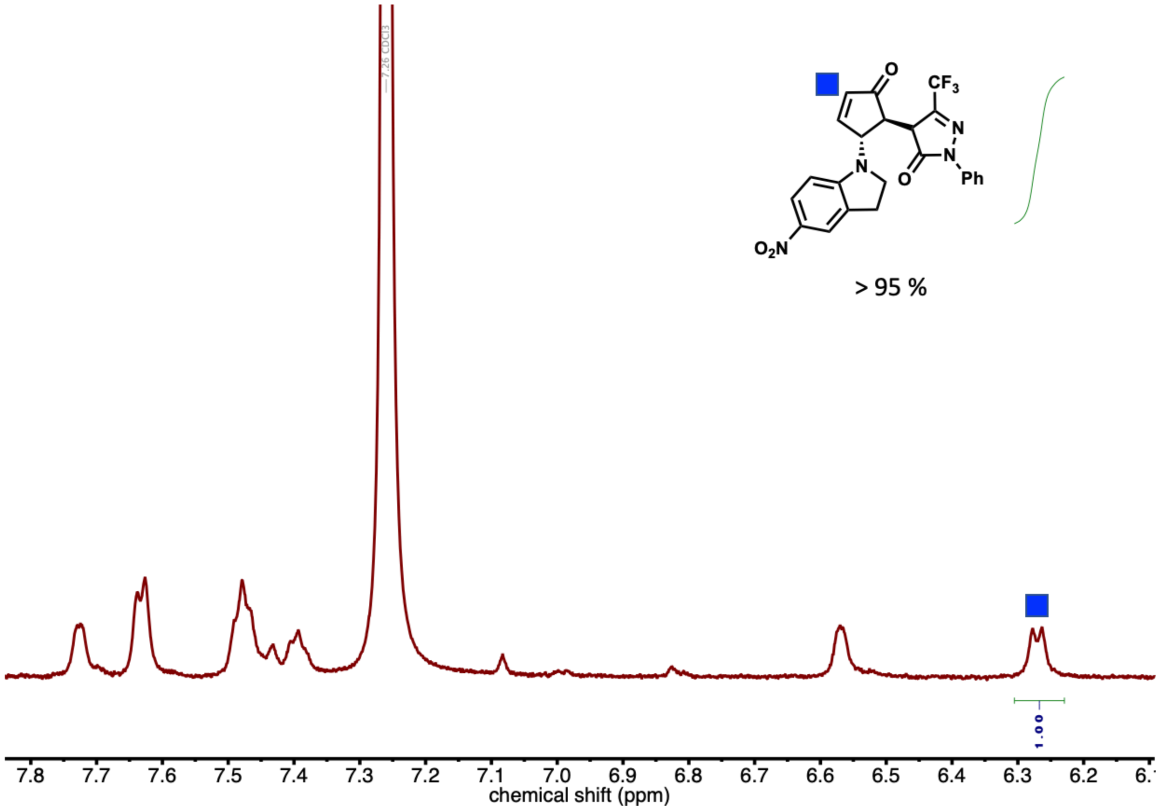

Figure S69: ^{1}H NMR (600 MHz, $CDCl_3$) spectra of the equilibrated DASA-**10**. No open form can be observed.

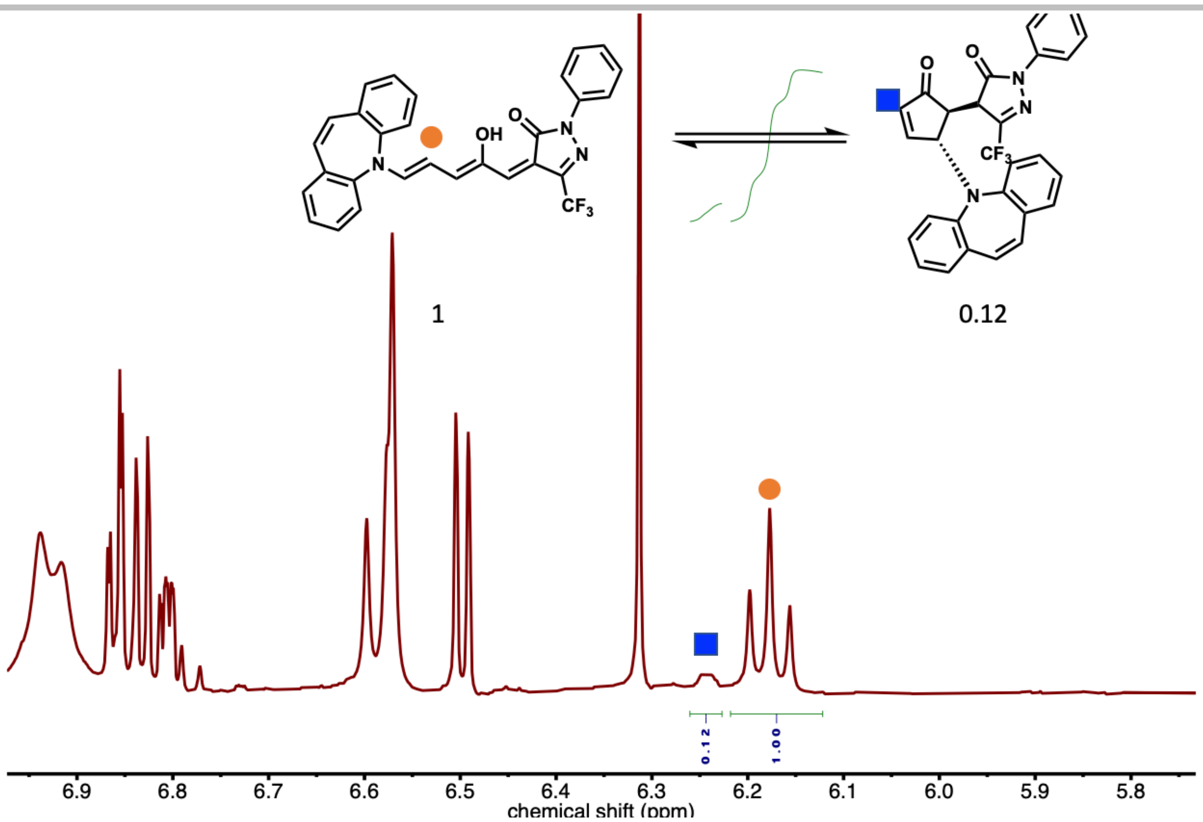

Figure S70: ^{1}H NMR (600 MHz, $CDCl_3$) spectra of the equilibrated DASA-**11**.

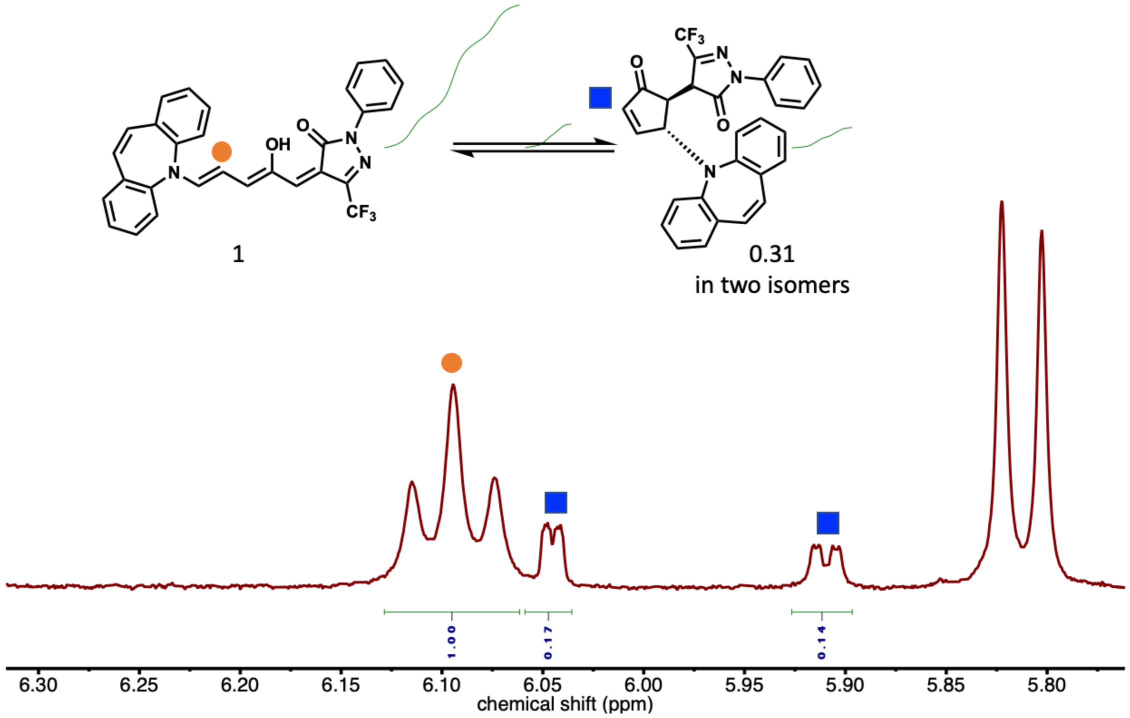

Figure S71: ^{1}H NMR (600 MHz, Tol-d_8) spectra of the equilibrated DASA-**11**.

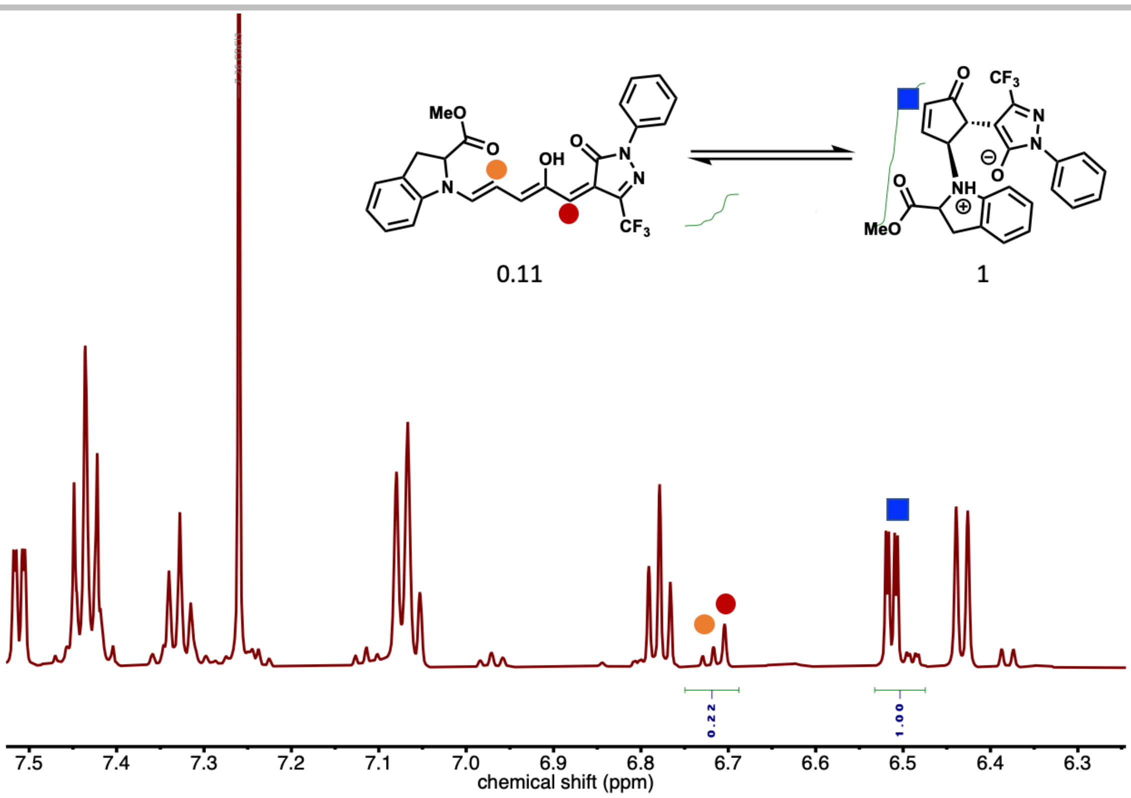

Figure S72: ^{1}H NMR (600 MHz, $CDCl_3$) spectra of the equilibrated DASA-**12**.

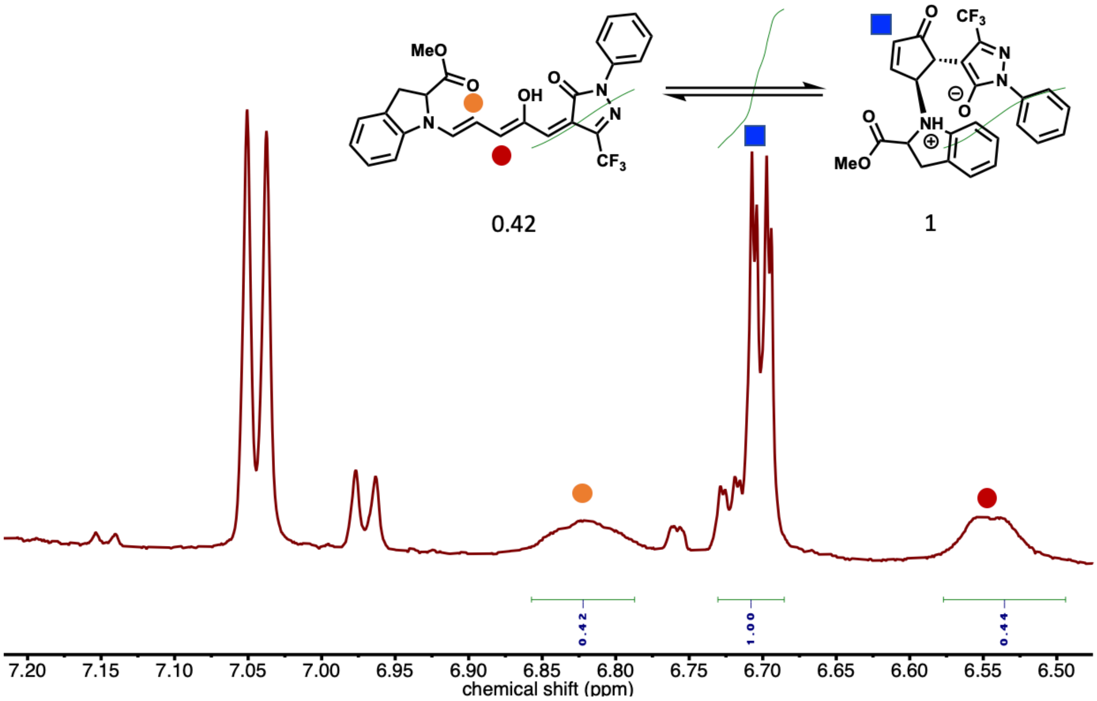

Figure S73: ^{1}H NMR (600 MHz, Tol-d_8) spectra of the equilibrated DASA-**12**.

8.4 2D-NMR Spectroscopy

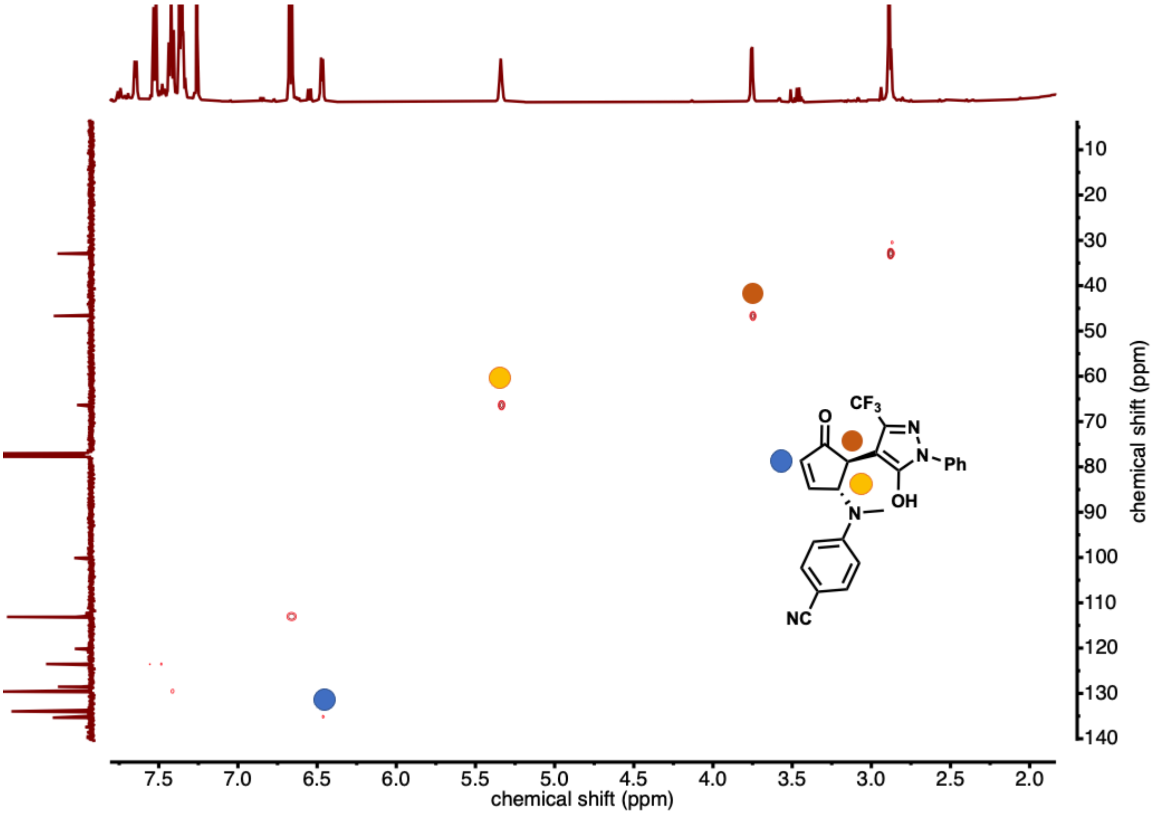

Figure S74: HSQC NMR (600 MHz, 297 K, $CDCl_3$) spectra of DASA-**8**.

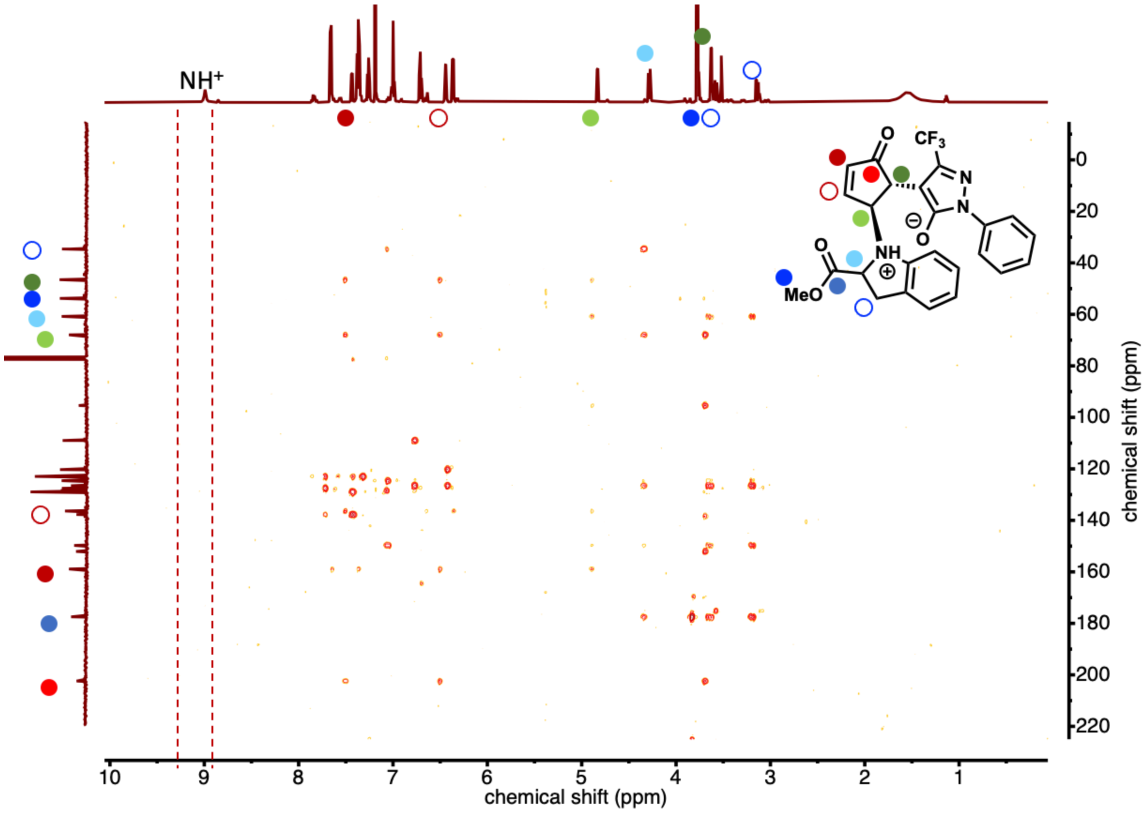

Figure S75: HMBC NMR (600 MHz, 297 K, $CDCl_3$) spectra of DASA-**12**.

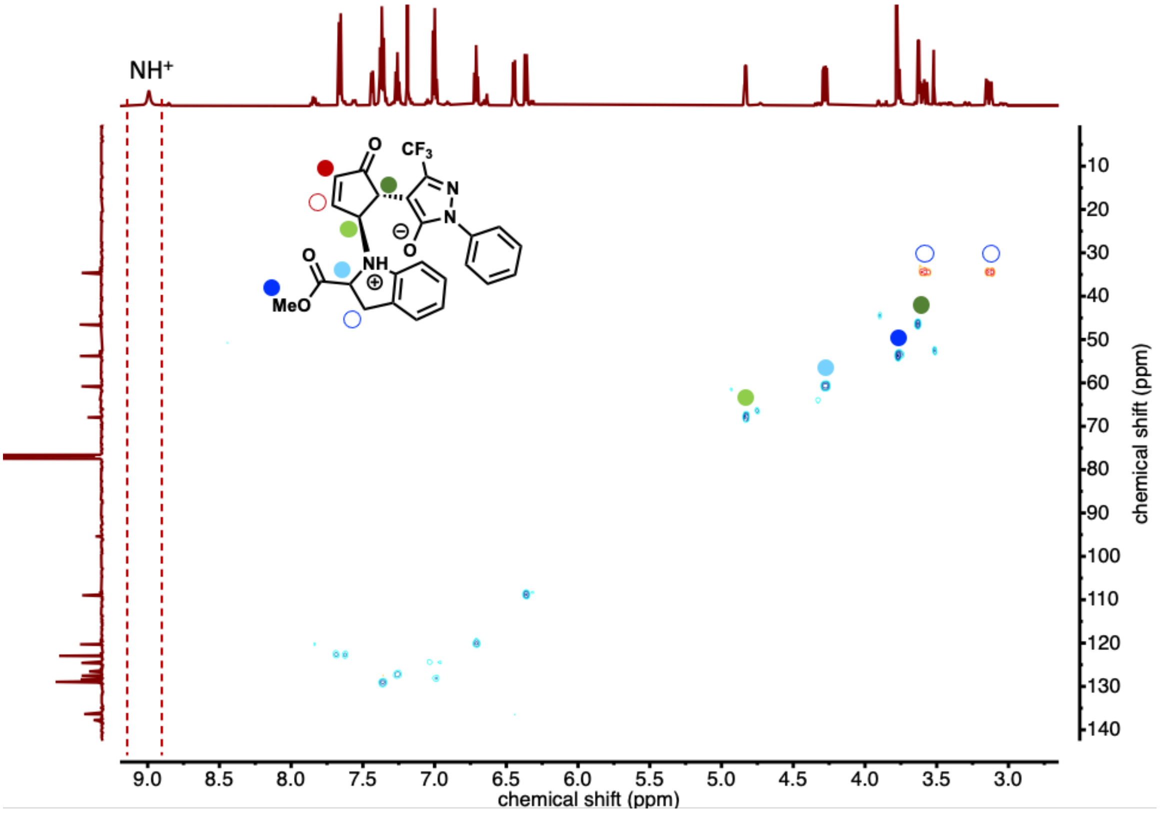

Figure S76: HSQC NMR (600 MHz, 297 K, $CDCl_3$) spectra of DASA-**12**.

9. Fluorescence

For DASA-**9** in toluene (in contrast to the parent 2,2'-dinaphthylamine) we found a dependence of the emission spectrum on the excitation wavelength (**Figure S77**). This suggests the existence of multiple electronic transitions being involved, which can be explained by the existence of different fluorescent species in the ground state (i.e. open/closed form DASA and/or conformers thereof and/or minor amounts of residual free amine and/or conformers thereof). In general, the emission spectra of DASA-**9** are slightly red-shifted when compared to the parent dinapthylamine in toluene (λ_{max} of 396 nm vs. 388 nm). For dinaphthylamine in toluene we did not observe any visible-light dependence on the fluorescent properties, while for DASA-**9** we observed an increase in intensity, but no change in band shape or position, when repeatedly irradiated with a MINTF4 fiber-coupled LED (Thorlabs, nominal wavelength 554 nm) for ~60 seconds (10 µM in toluene, **Figure S78**). Control measurements of dinaphthylamine (0.1 µM) in a toluene solution of DASA-**2** (20 µM), however, did show some degree of excitation wavelength dependent emission spectra and trials with visible light irradiation were inconclusive (**Figure S79**). We can therefore not exclude that trace amounts of free dinaphthylamine are causing or interfering with the measured fluorescence response. We additionally found that a toluene solution of DASA-**9** when constantly irradiated with 350 nm light shows irreversible increase in fluorescence emission after some time suggesting possible photodegradation (no such changes were found for dinaphthylamine in toluene in the investigated time window, **Figure S79**). As described elsewhere,[15] open form DASAs exhibit weak fluorescence emission when excited at their maximum absorption wavelength (π-π* transition). We also observed fluorescence in DASA-**9** upon excitation at 630 nm but similarly weak in intensity as observed previously for other DASA derivatives employing simpler amine donors. The intensity of this fluorescence band decreases upon converting the DASA to the closed form isomer and increases again upon thermal recovery in the dark (**Figure S78)**.

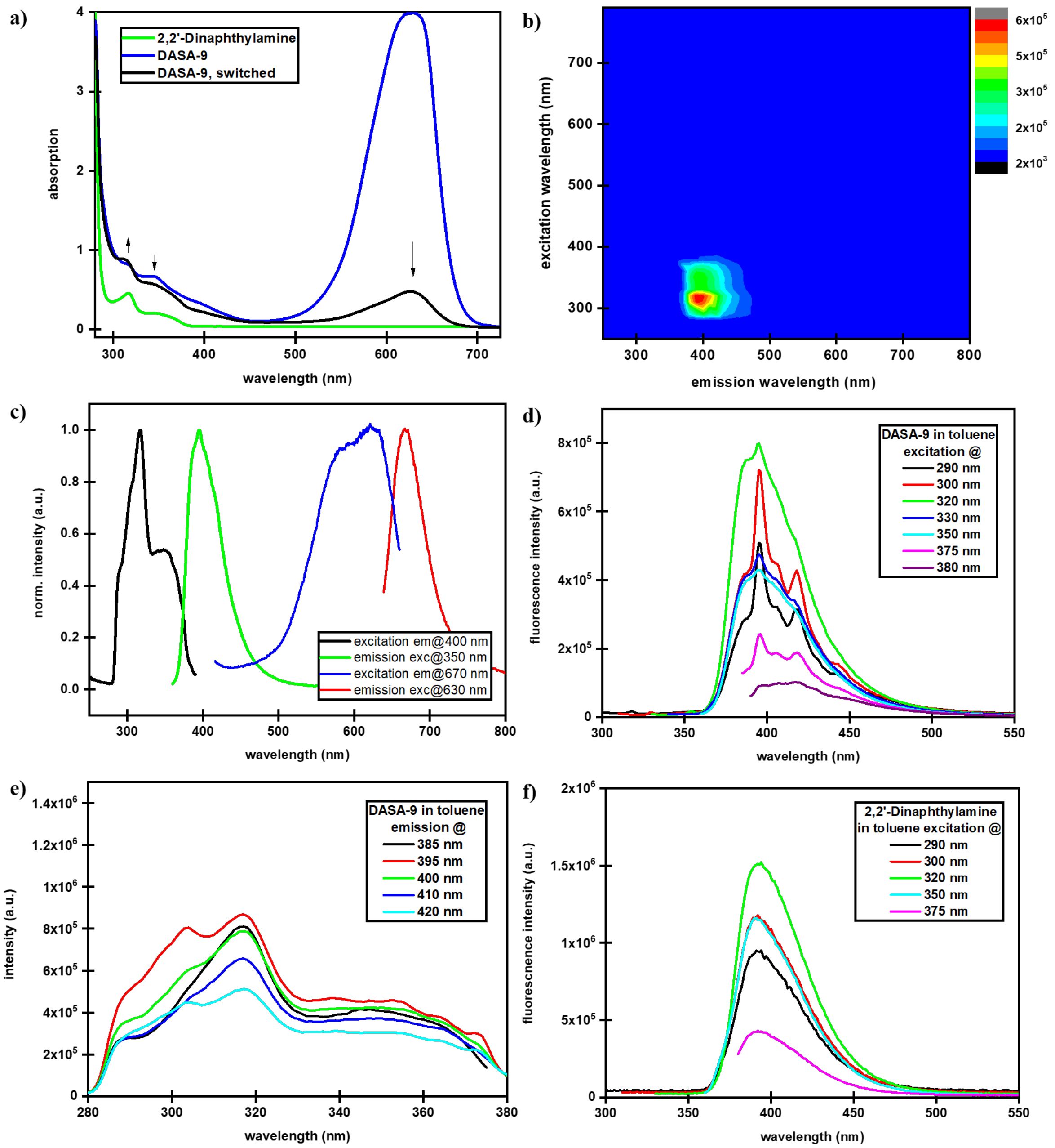

Figure S77. Absorption, emission and excitation spectra of DASA-**9** (equilibrated solution in toluene) and 2,2'-dinaphthylamine in toluene. a) Absorption spectra showing the absorbance bands characteristic for the naphthyl-units at 325–350 nm (2,2'-dinaphhylamine, 20 µM; DASA-**9**, 100 µM, before and after visible light irradiation for ~100 s). b) Excitation vs emission spectrum of an equilibrated solution of DASA-**9** in toluene (10 µM). c) Normalized excitation and emission spectra: λ_{em} = 400 nm/ λ_{exc} = 350 nm (2 nm slit widths) and λ_{em} = 670 nm/ λ_{exc} = 630 nm (5 nm slit widths). d) Emission spectra of DASA-**9** in toluene (10 µM) for different excitation wavelengths. e) Excitation spectra of DASA-**9** in toluene (10 µM) for different emission wavelengths. f) Emission spectra of 2,2'-dinaphthylamine in toluene (20 µM) for different excitation wavelengths (1 nm slit widths).

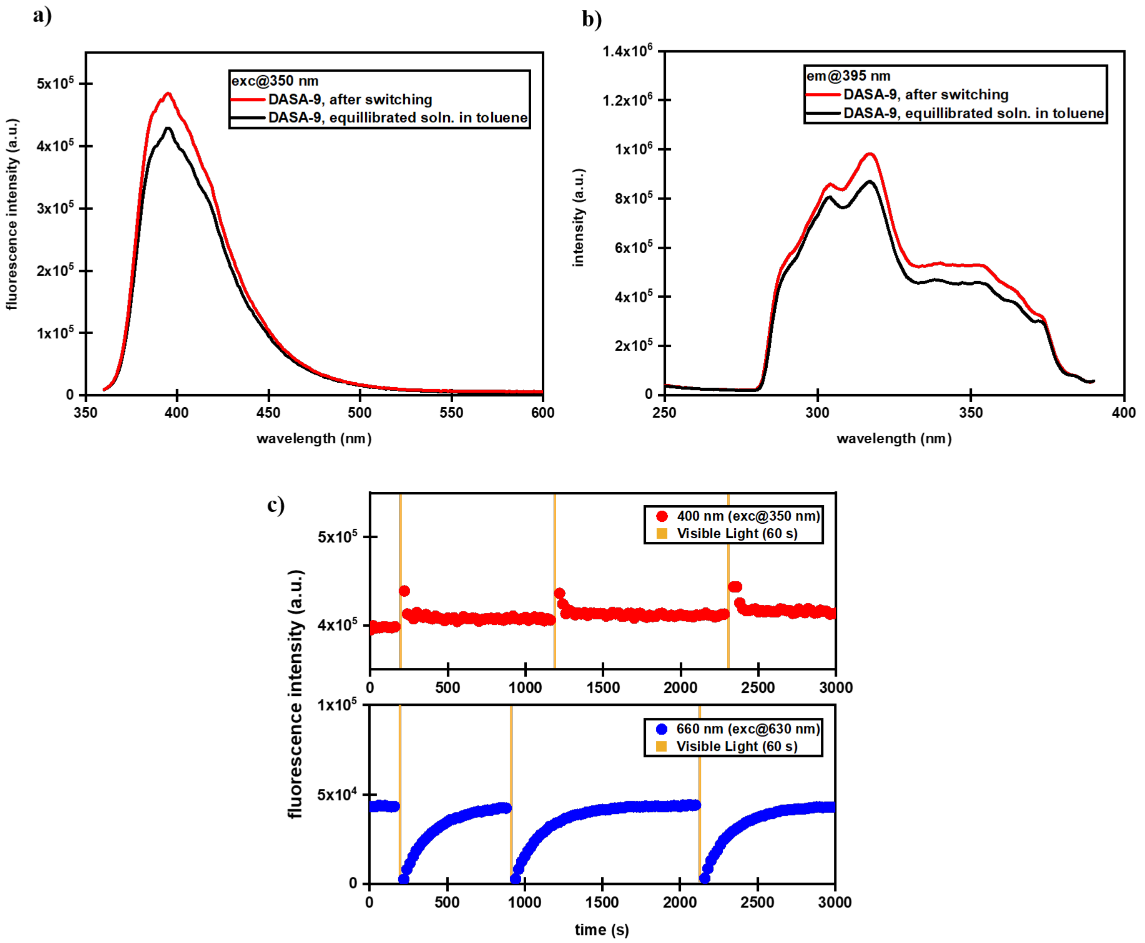

Figure S78: Changes in emission and excitation spectra of DASA-**9** (10 µM, toluene). a) Emission spectra (λ_{exc} = 350 nm) before and after visible light irradiation (MINTF4 fiber-coupled LED, Thorlabs, nominal wavelength 554 nm) for ~60 seconds. b) Excitation spectra (λ_{em} = 395 nm) before and after visible light irradiation. c) Fluorescence intensity monitoring at 400 nm (λ_{exc} = 350 nm, 2 nm slit widths) and 660 nm (λ_{exc} = 630 nm, 5 nm slit widths) before and after visible light irradiation (MINTF4 fiber-coupled LED, Thorlabs, nominal wavelength 554 nm for ~60 seconds inside fluorimeter, temperature: 31 °C).

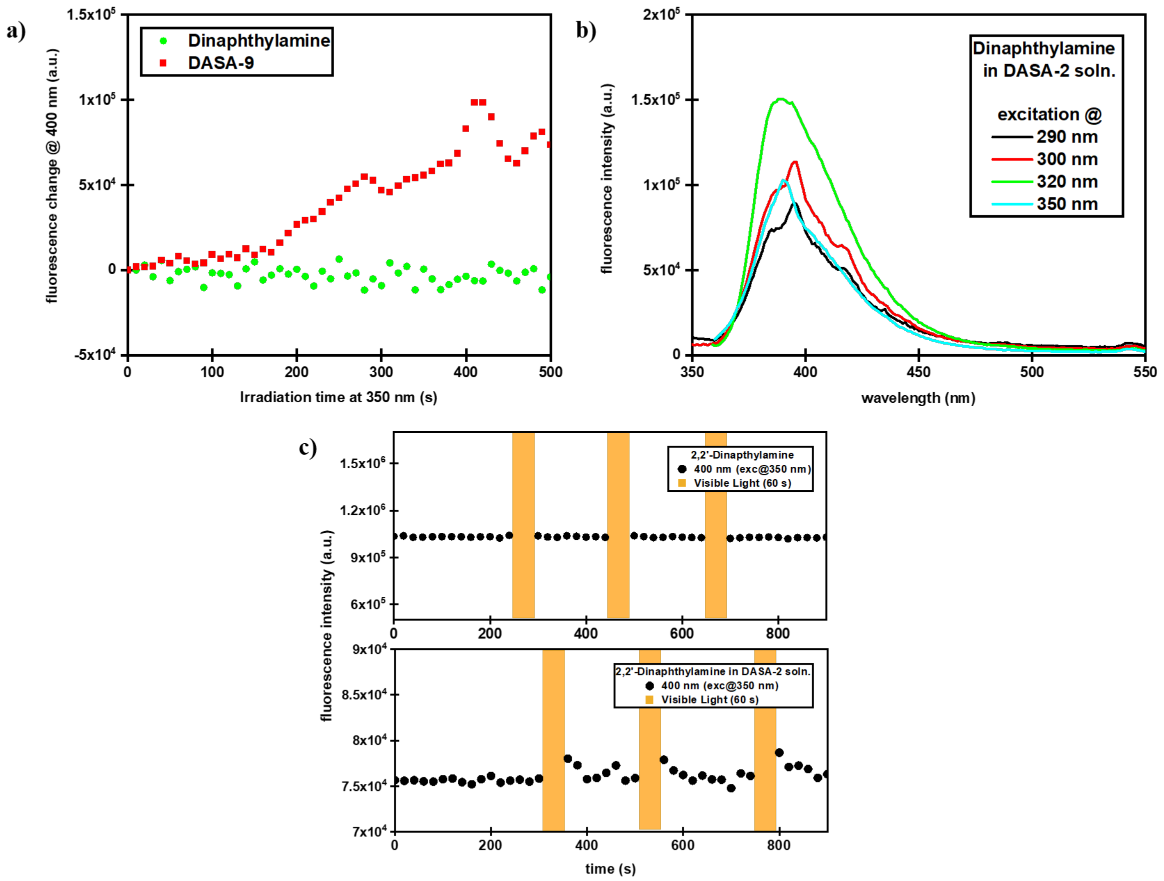

Figure S79: Control experiments for luminescence of DASA-**9**. a) Fluorescence intensity change at 400 nm (λ_{exc} = 350) upon constant irradiation at 350 nm (excitation light source of fluorimeter) for solutions of dinaphthylamine (20 µM in toluene) and DASA-**9** (10 µM in toluene). b) Emission spectra at different excitation wavelengths of dinaphthylamine (0.1 µM) in a solution of DASA-**2** in toluene (20 µM). Slit widths set to 3 nm. c) Fluorescence intensity monitoring at 400 nm (λ_{exc} = 350 nm) before and after visible light irradiation (MINTF4 fiber-coupled LED, Thorlabs, nominal wavelength 554 nm for ~60 seconds inside fluorimeter, temperature: 31 °C) of dinaphthylamine in toluene (20 µM) or in a toluene solution (0.1 µM) of DASA-**2** (20 µM).

10. Probing the H-bonding in the Presence of HFIP

As confirmed in previous studies by other groups,[10] the hydroxy protons in DASAs are easily exchangeable and their ^{1}H-NMR signals are completely absent when the spectra are measured in, e.g., deuterated alcohols. We also observed the disappearance of these signals when measuring ^{1}H-NMR spectra of DASAs in solutions containing larger amounts of HFIP (deuterated and non-deuterated). In the following **Figure S80,** the changes of the hydroxy proton ^{1}H-NMR signal under different conditions are displayed. Here, it is visible that HFIP leads to a disappearance of the hydroxy proton signal, whereas additives such as HFIPMe at similar concentrations have none (or less, only slight broadening is observed) of an effect.

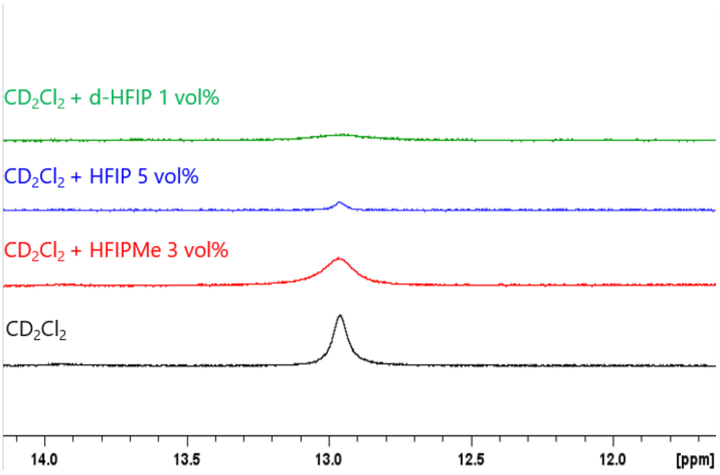

Figure S80: ^{1}H NMR (500 MHz, CD_2Cl_2) spectra of DASA-**2** upon addition of different additives. The signal of hydroxy proton on the triene is shown.

As also shown in **Figure S81**, the presence of HFIP, however, seems to not effect a change of the chemical shift of the OH-signal. On the other hand, the chemical shifts of the polyene and aromatic protons do change, which we believe can also be a result of simple solvent effects and not necessarily specific interactions (it is also established that different solvents favor different resonance structures of the DASA open form).

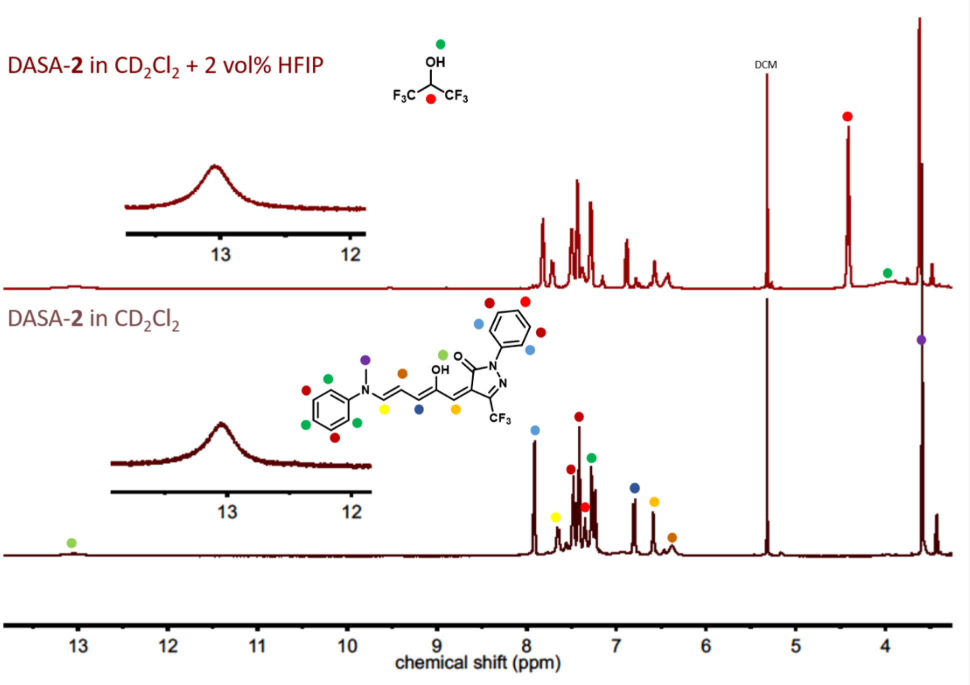

Figure S81: ^{1}H NMR (500 MHz) spectra of DASA-**2** (10 mg /mL) in CD_2Cl_2 and CD_2Cl_2 containing 2 vol% HFIP.

To further probe the potential of H-bonding, IR spectroscopic measurements in DCM vs. HFIP on a Meldrum's acid based DASA (DASA-**6**) were conducted. As indicated in **Figure S82** below, the observation of two bands for the carbonyl stretching modes in the Meldrum's acid moiety can be found for measurements in DCM or in the solid state (~1615 cm^{-1}: H-bonded ring carbonyl stretch, ~1700 cm^{-1} second non H-bonded carbonyl, in agreement with previous assignments from literature).[10] On the other hand, measurements in HFIP did not show a pronounced band at 1700 cm^{-1} for a non H-bonded carbonyl and the band previously observed at ~1615 cm^{-1} red-shifts pointing towards a large change in the strength of the H-bond. Although further studies are required, this does provide additional supporting evidence for the proposed modulation of the H-bonding.

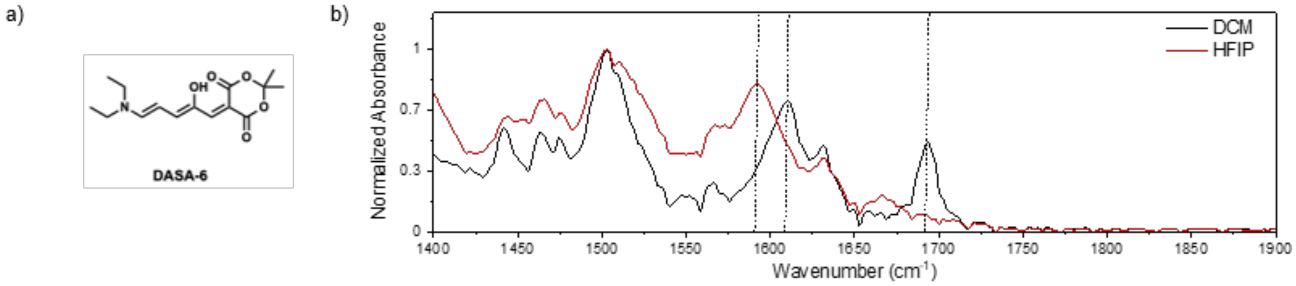

Figure S82: a) DASA-**6**. b) IR spectra of DASA-**6** in DCM and HFIP showing the spectral changes in carbonyl region.

11. Spectra

11.1 NMR Spectra

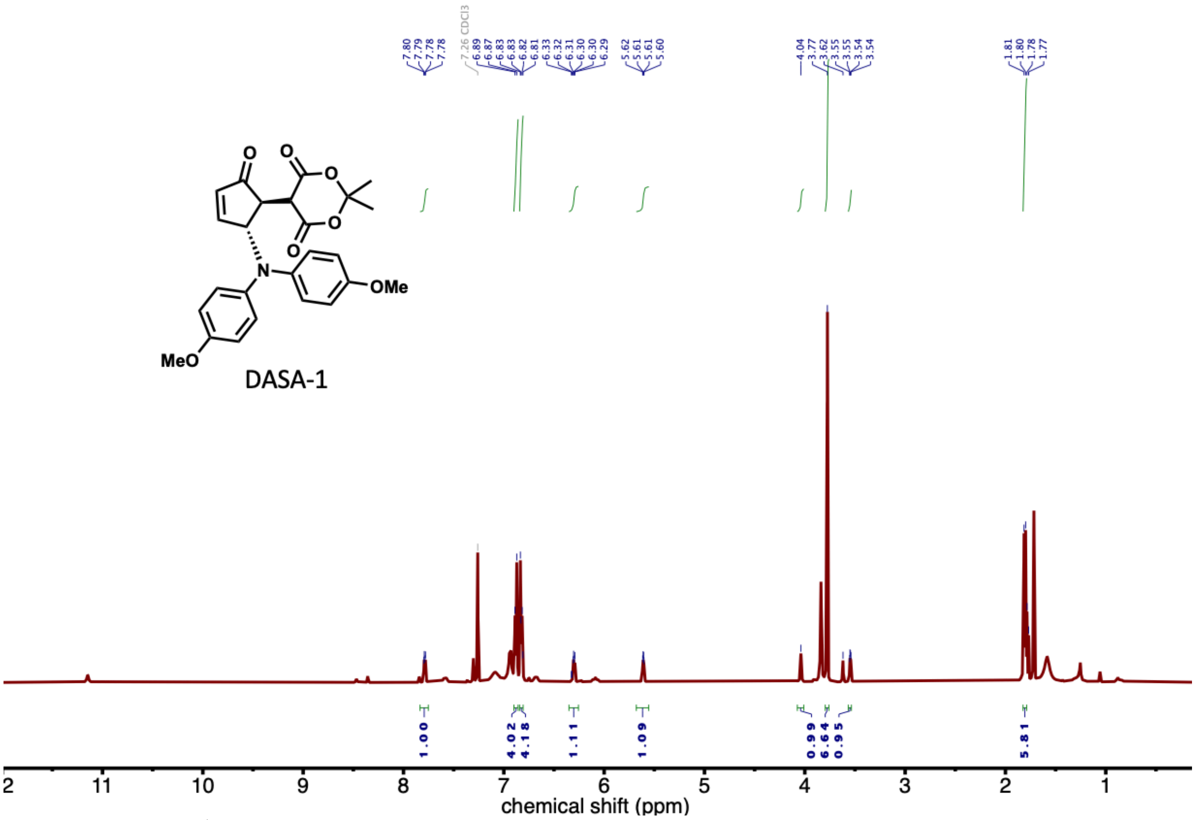

Figure S83: ^{1}H NMR (500 MHz, $CDCl_3$) spectra of the closed isomer of DASA-**1**. Some open form can also be observed.

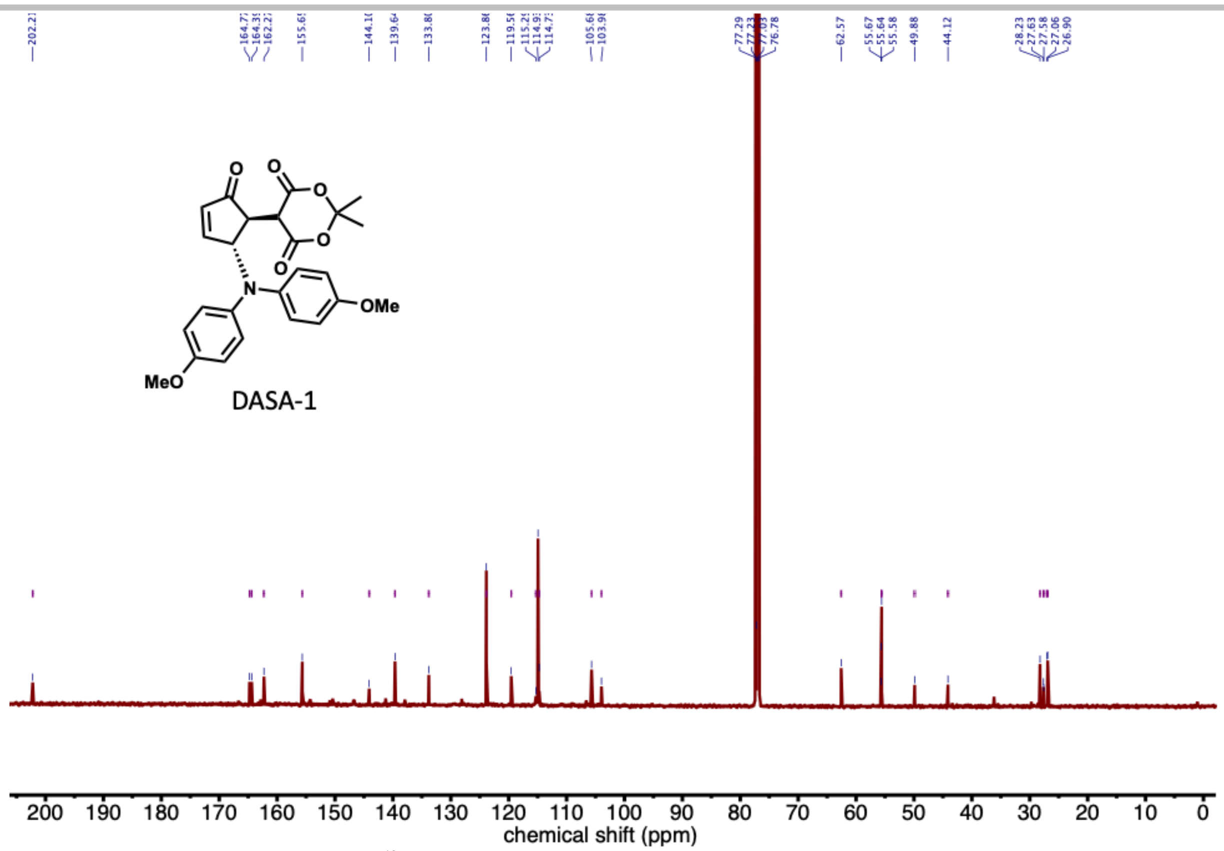

Figure S84: ^{13}C NMR (125 MHz, $CDCl_3$) spectra of the closed isomer of DASA-**1**.

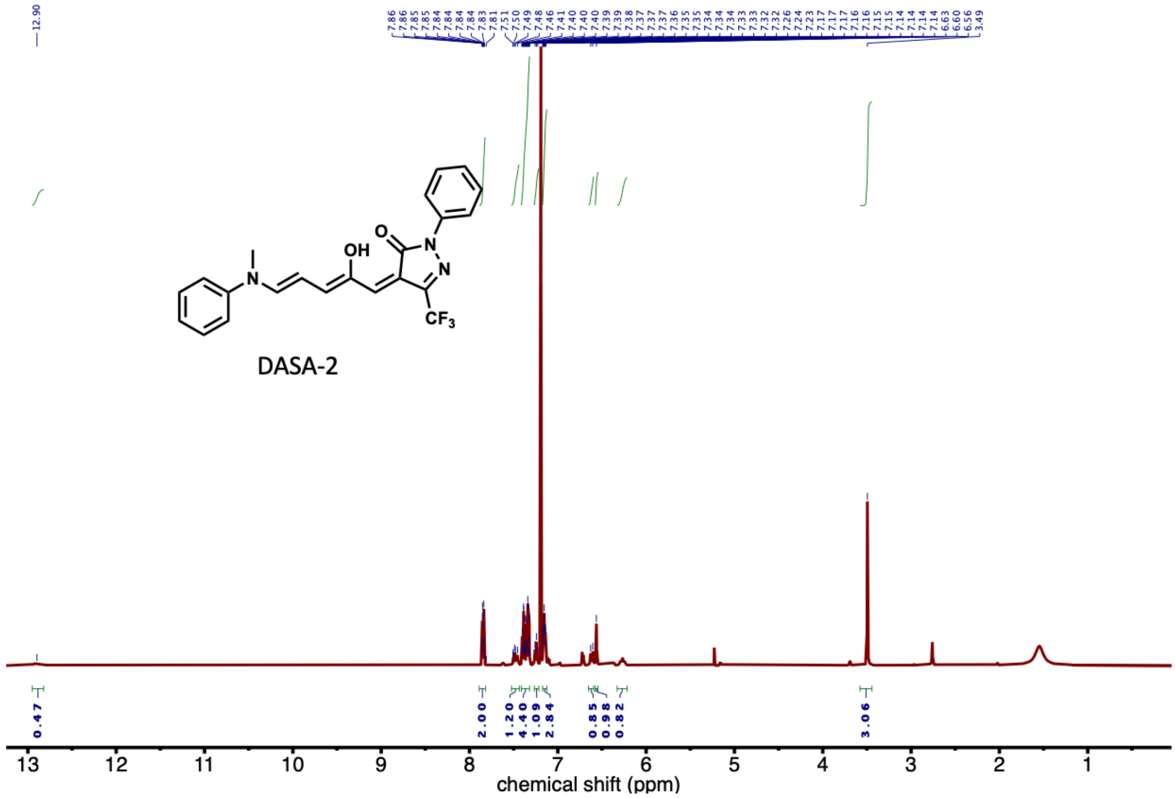

Figure S85: ^{1}H NMR (500 MHz, $CDCl_3$) spectra of the open isomer of DASA-**2**. Some closed form can also be observed.

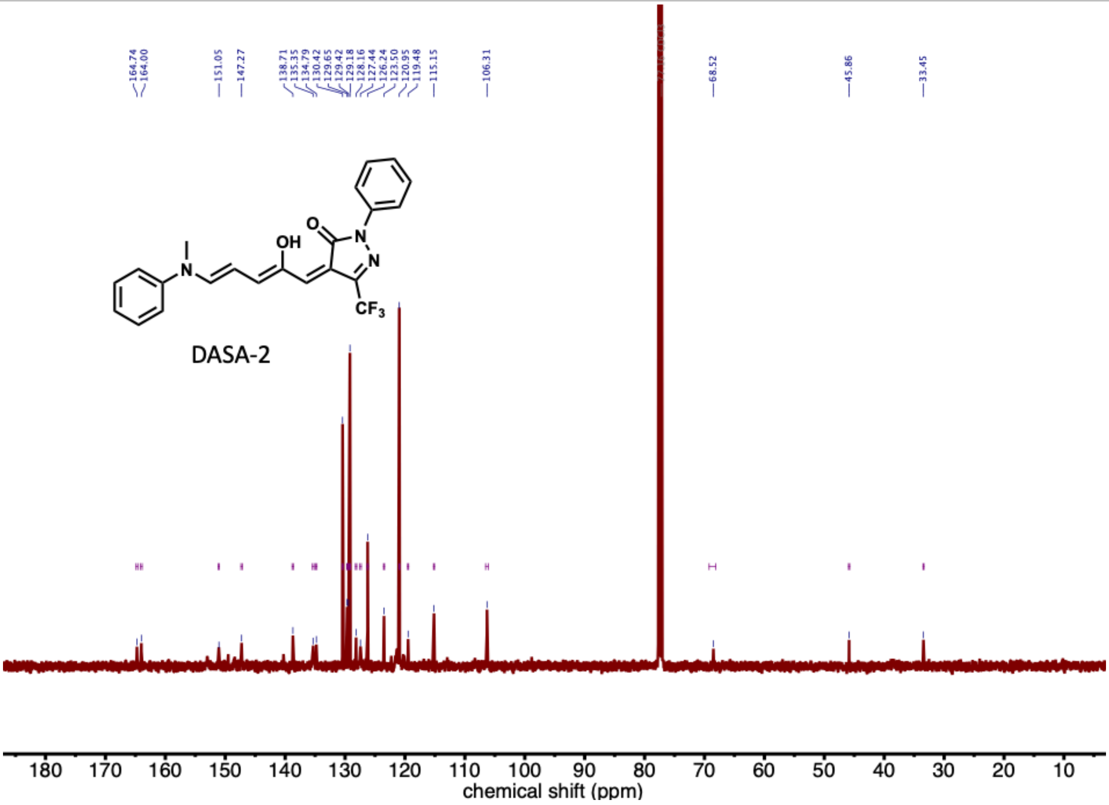

Figure S86: ^{13}CNMR (125 MHz, $CDCl_3$) spectra of the open isomer of DASA-**2**

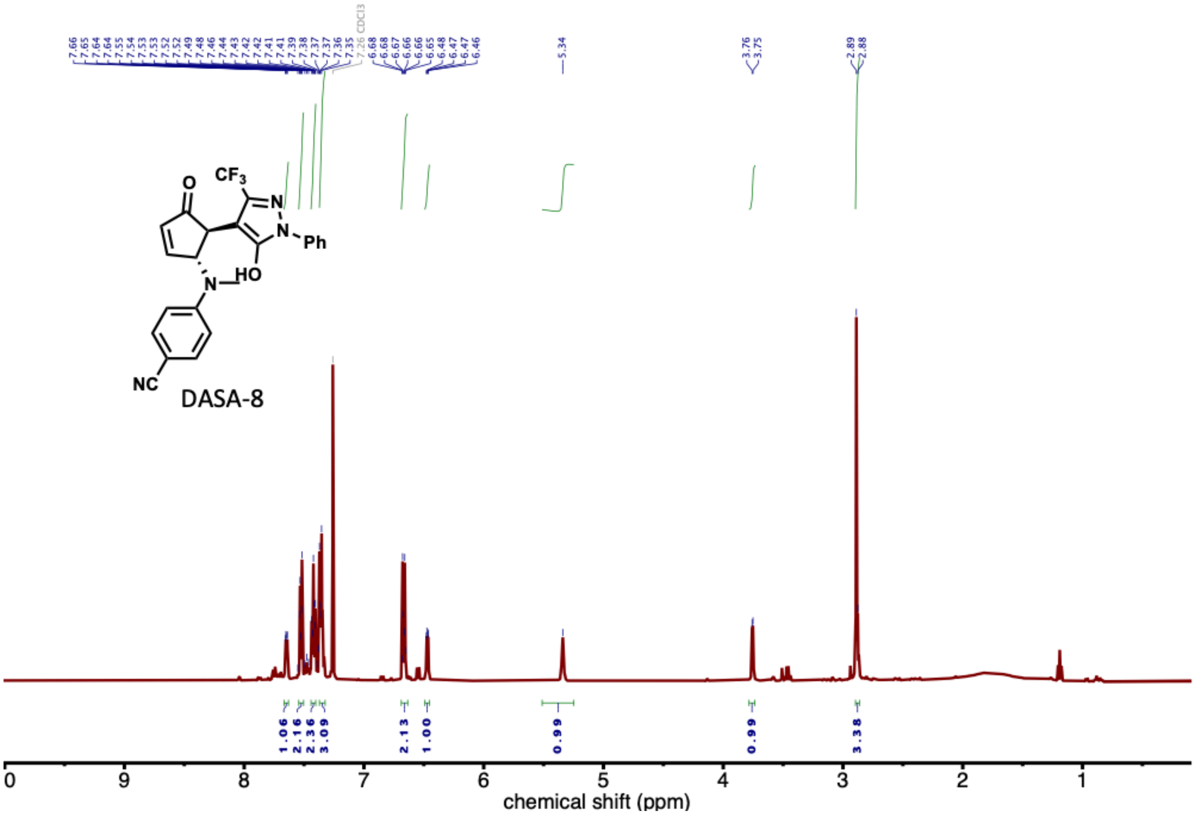

Figure S87: ^{1}H NMR (500 MHz, $CDCl_3$) spectra of the closed isomer of DASA-**8**.

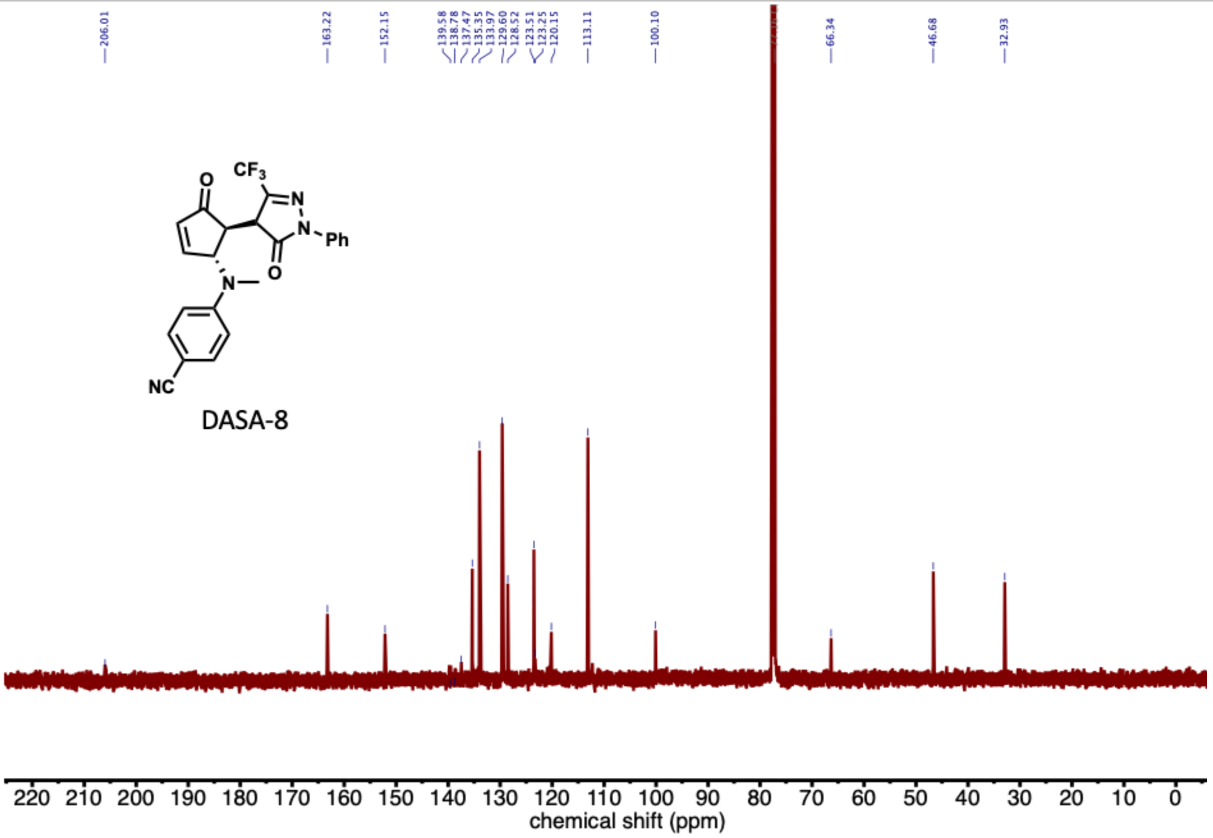

Figure S88: 1H NMR (125 MHz, CDCl3) spectra of the closed isomer of DASA-**8**.

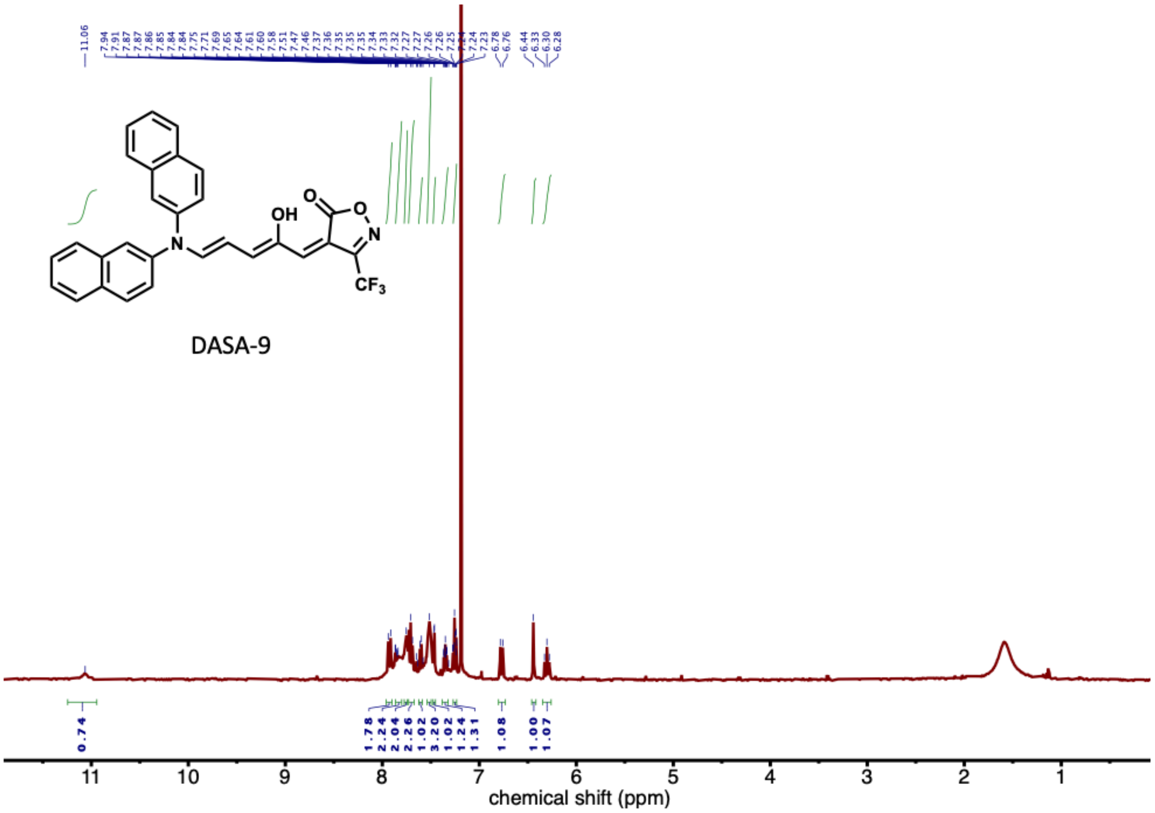

Figure S89: 1H NMR (500 MHz, CDCl3) spectra of the open isomer of DASA-**9**.

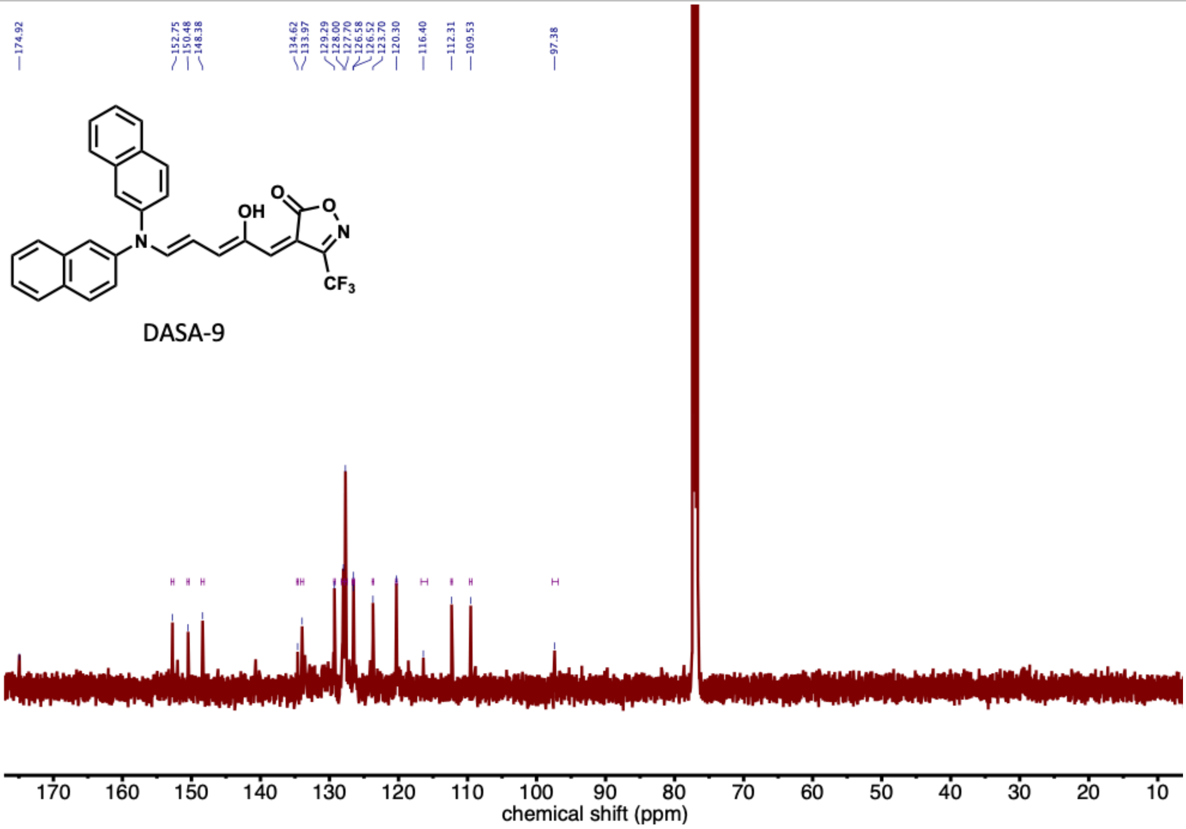

Figure S90: ^{13}C NMR (125 MHz, $CDCl_3$) spectra of the open isomer of DASA-**9**.

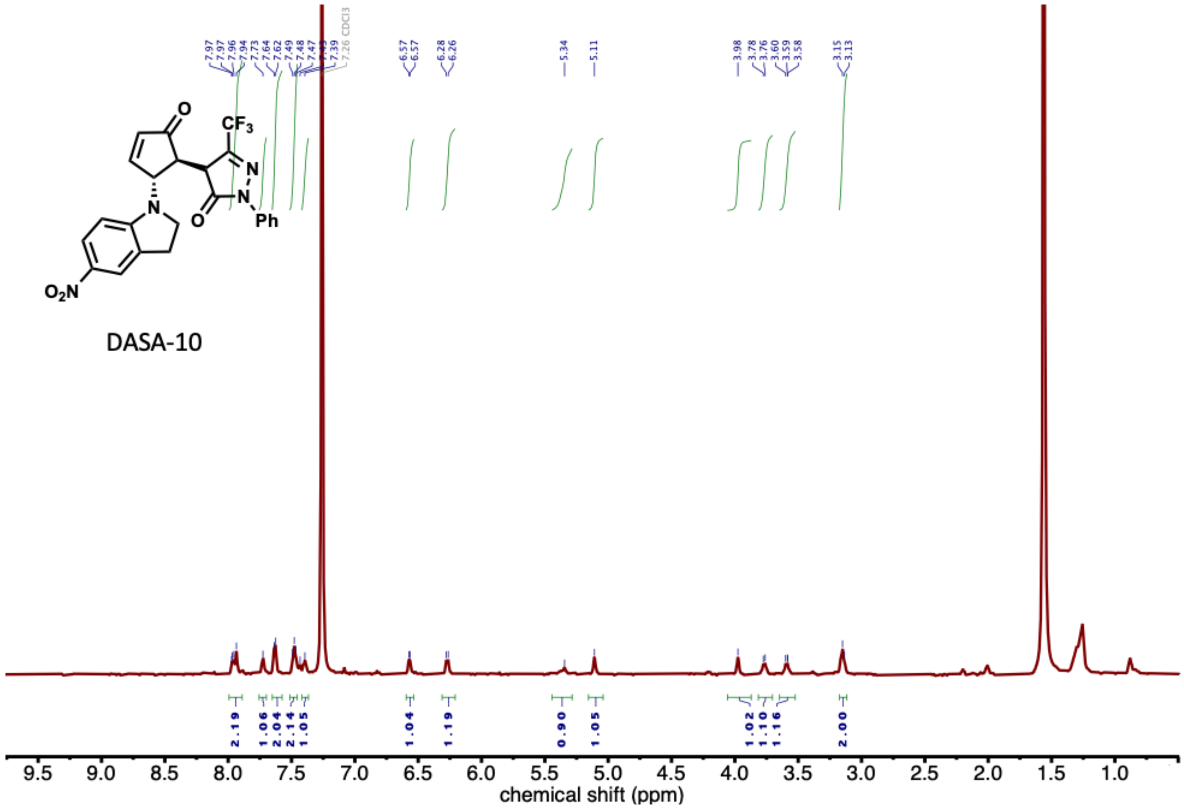

Figure S91: ^{1}H NMR (600 MHz, $CDCl_3$) spectra of the closed isomer of DASA-**10**.

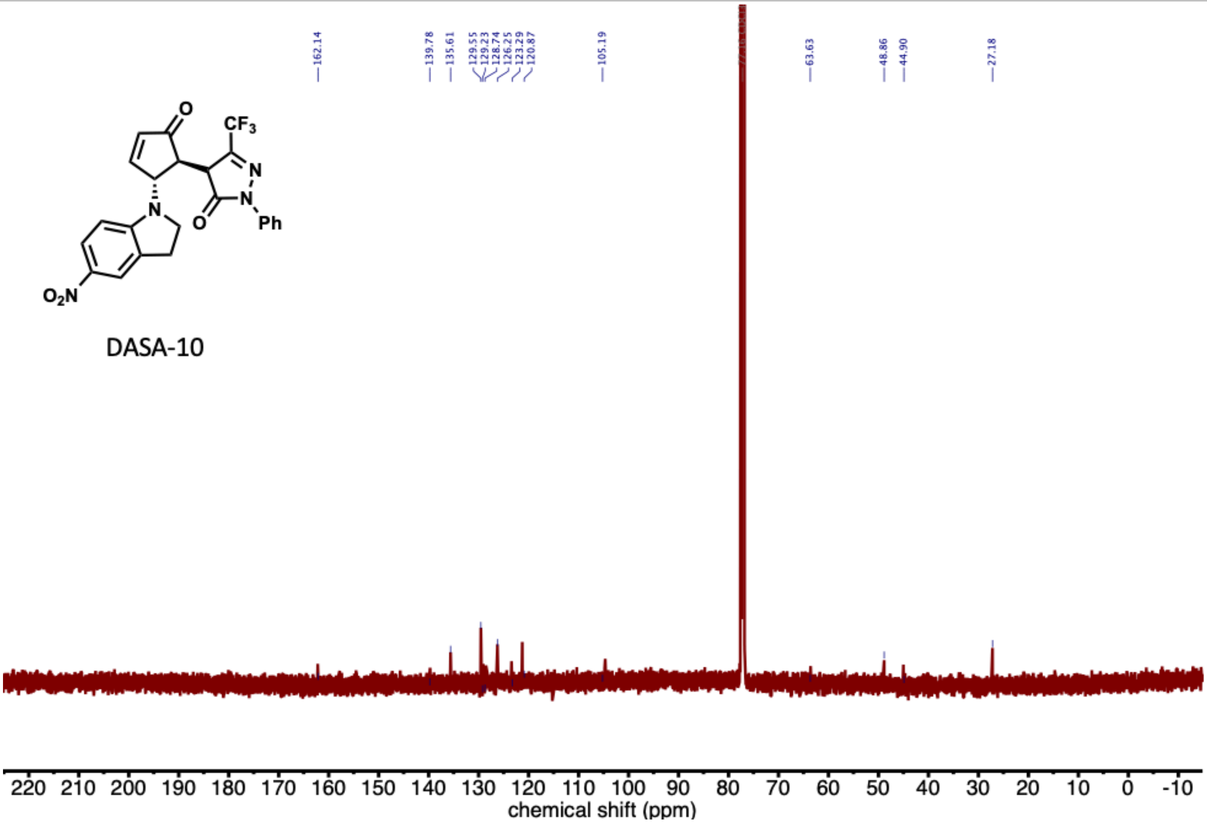

Figure S92: ^{13}C NMR (125 MHz, $CDCl_3$) spectra of the open isomer of DASA-**10**. Limited solubility of the compound.

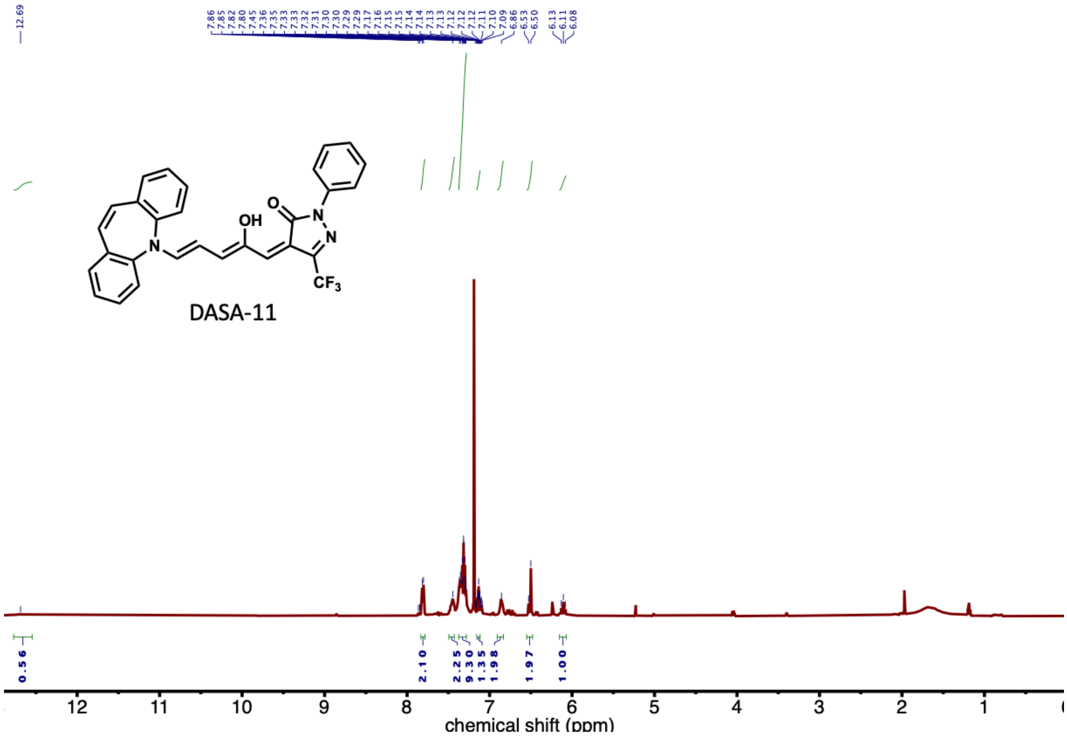

Figure S93: ^{1}H NMR (500 MHz, $CDCl_3$) spectra of the open isomer of DASA-**11**. Small amounts of closed isomer are also visible.

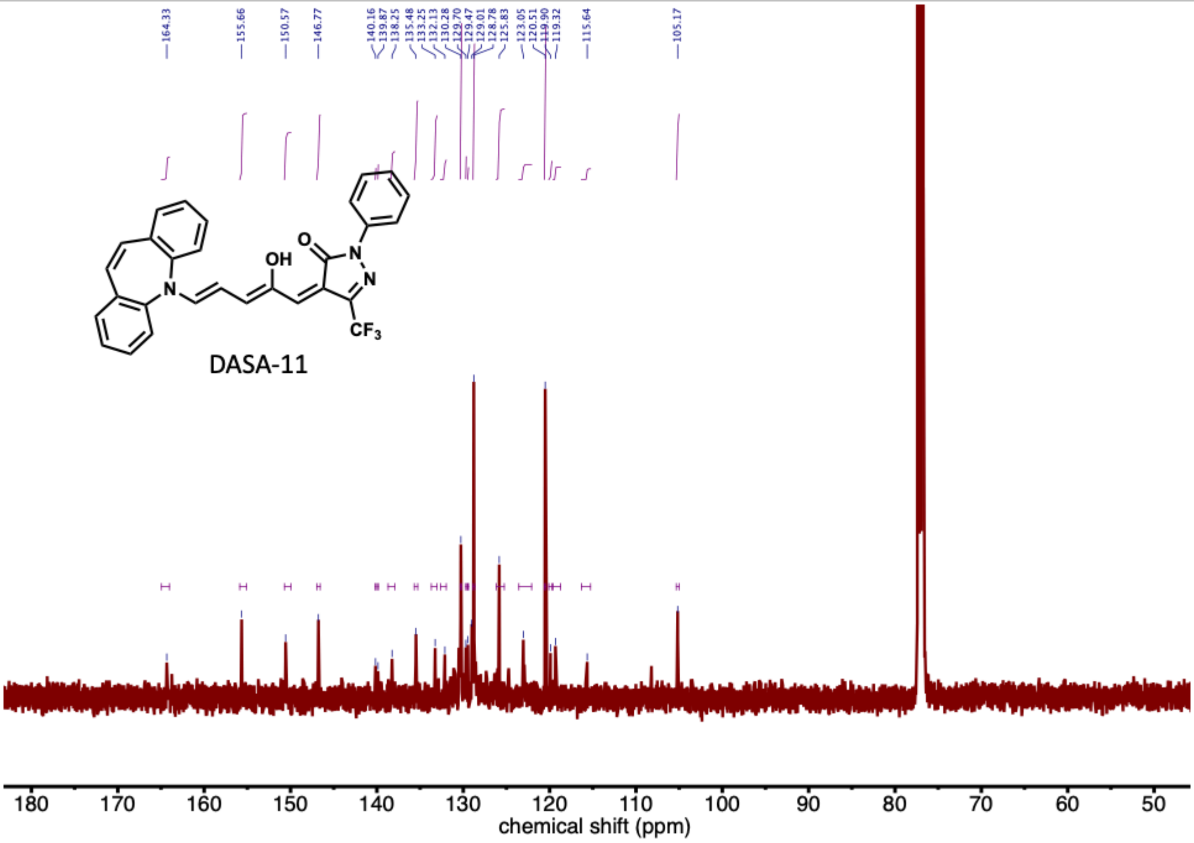

Figure S94: ^{13}C NMR (125 MHz, $CDCl_3$) spectra of the open isomer of DASA-**11**.

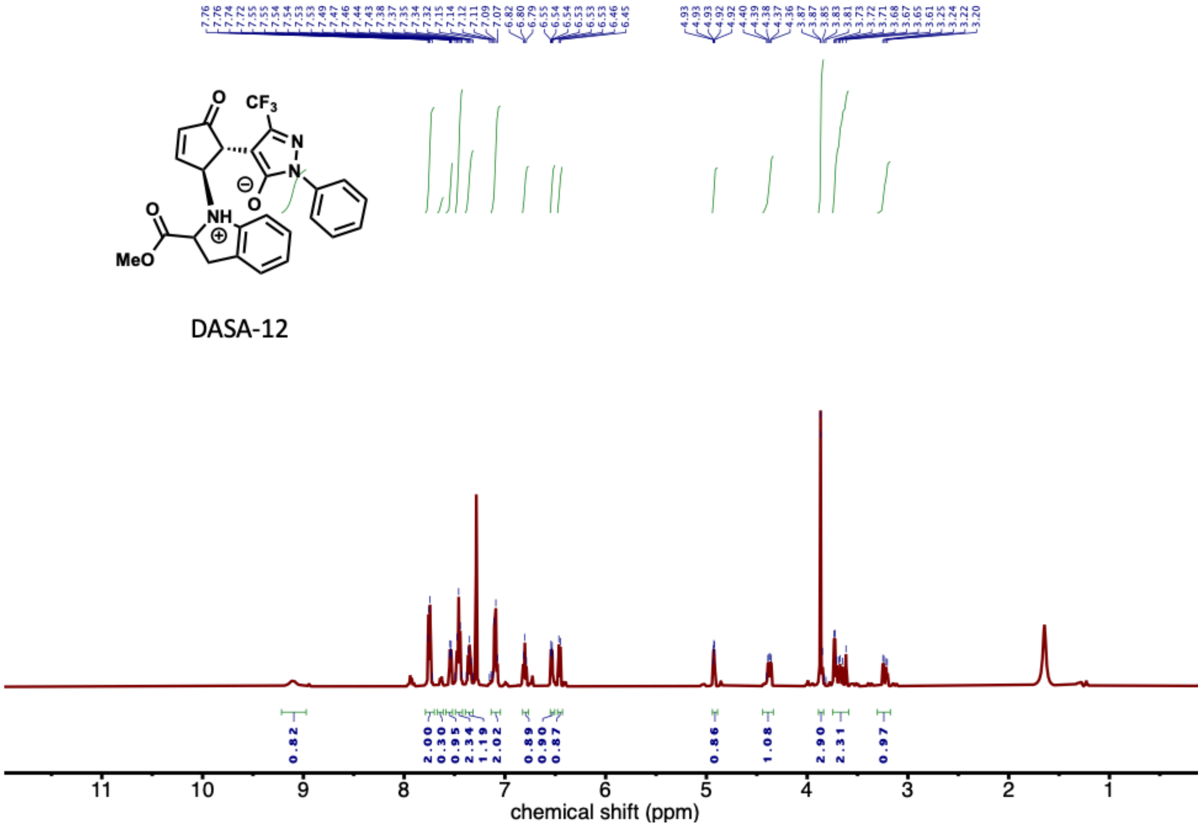

Figure S95: ^{1}H NMR (500 MHz, $CDCl_3$) spectra of the closed isomer of DASA-**12**. Small amounts of open form are also visible.

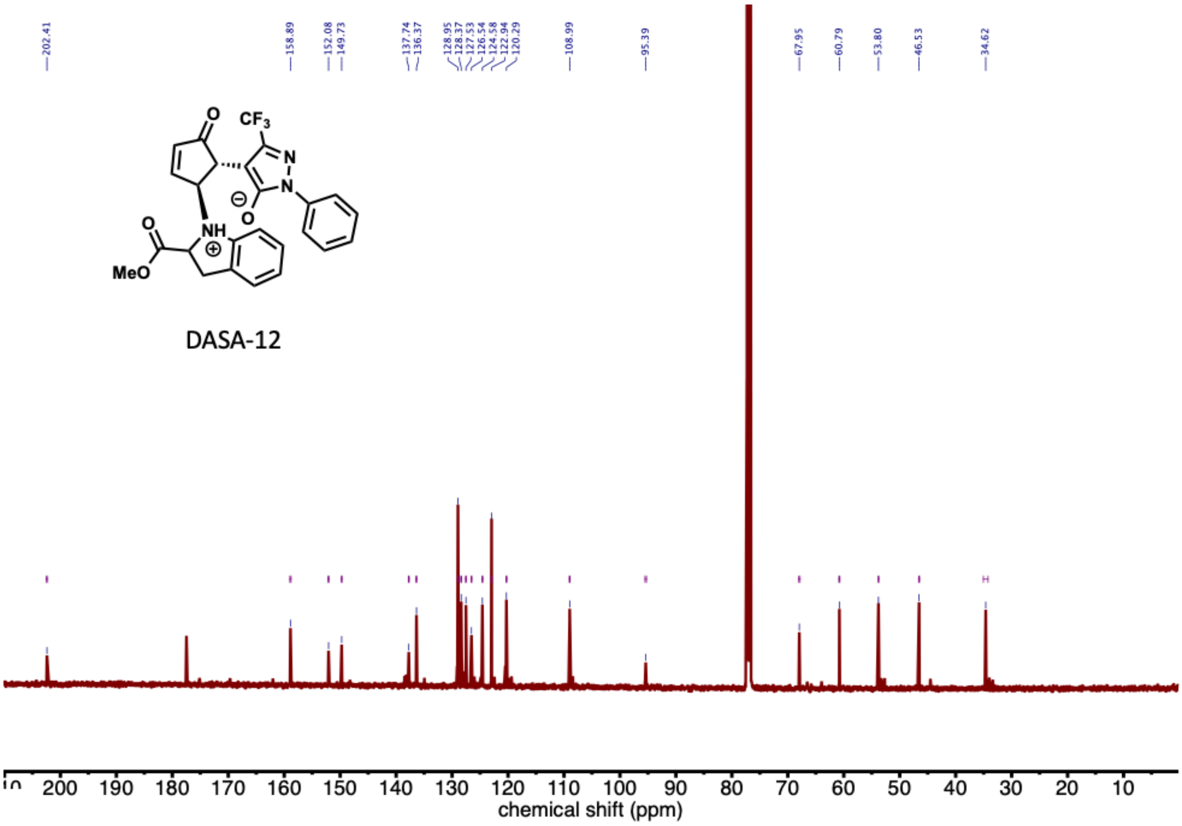

Figure S96: ^{13}C NMR (125 MHz, $CDCl_3$) spectra of the closed isomer of DASA-**12**.

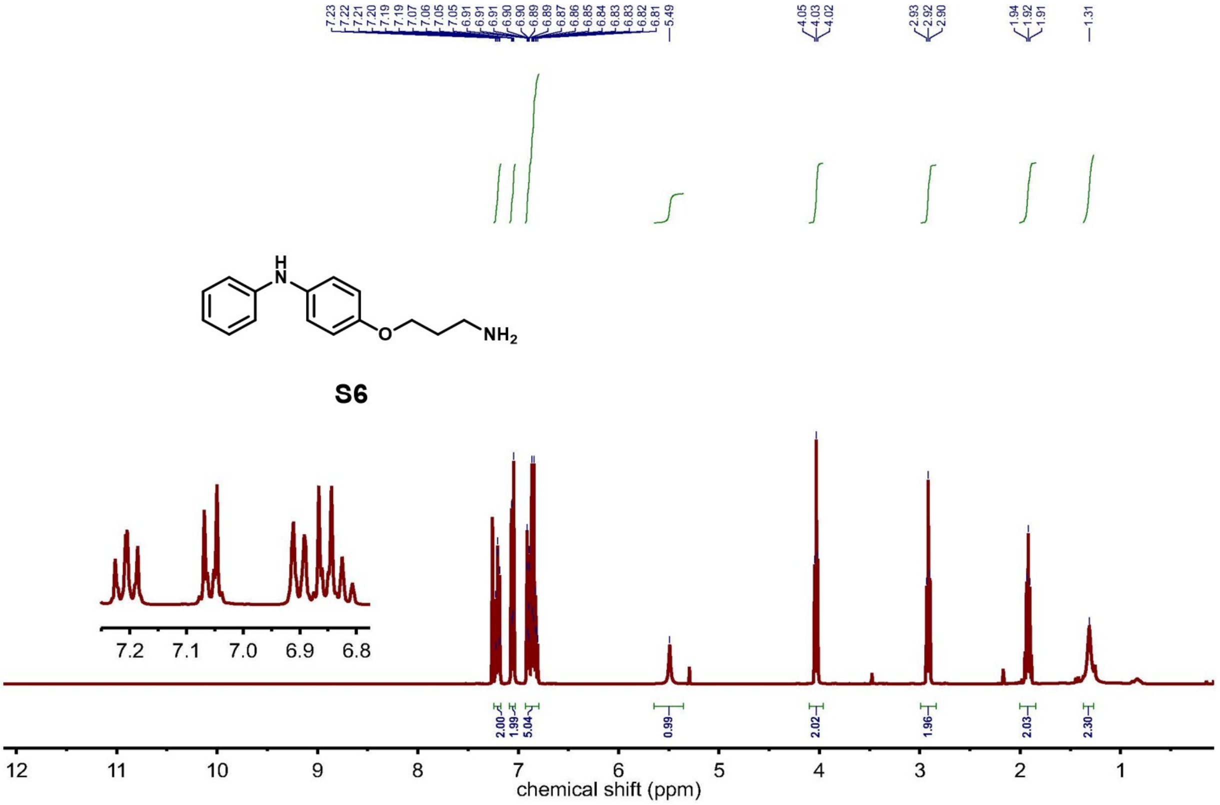

Figure S97: ^{1}H NMR (400 MHz, $CDCl_3$) spectra of **S6**.

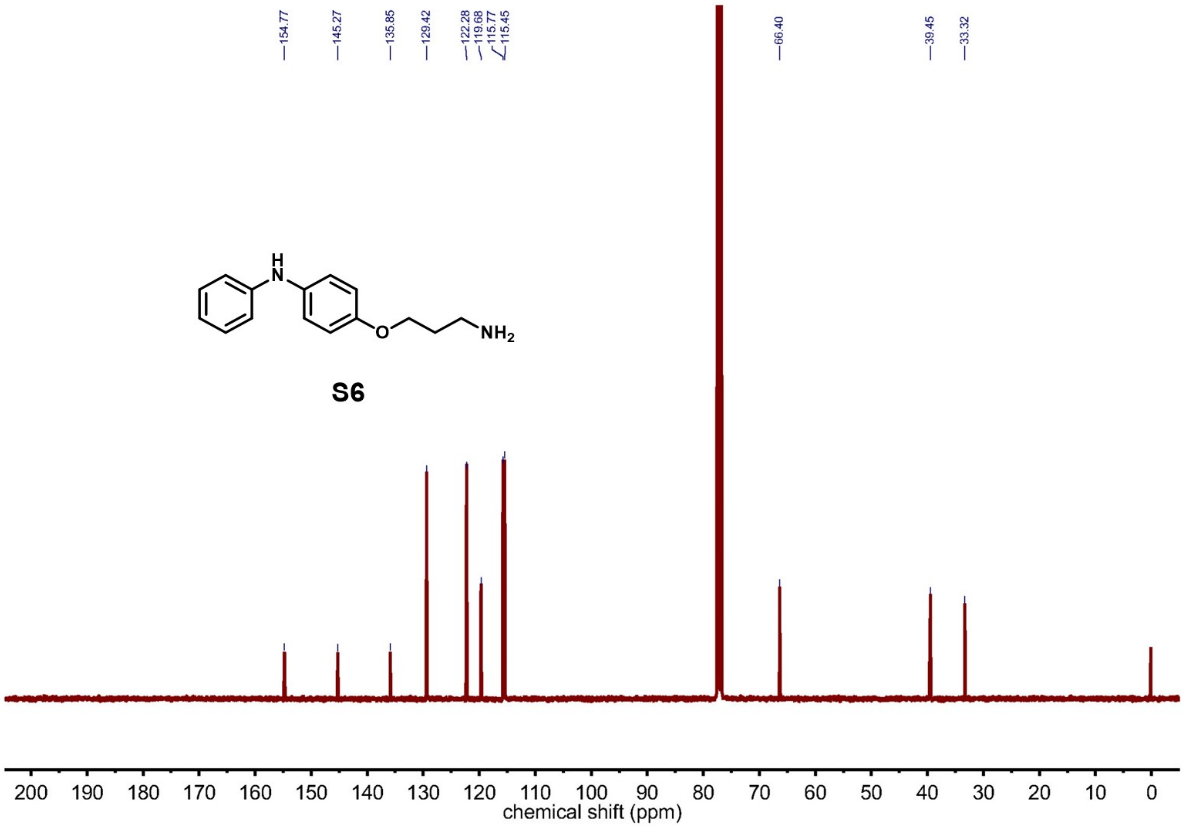

Figure S98: ^{13}C NMR (100 MHz, $CDCl_3$) spectra of **S6**.

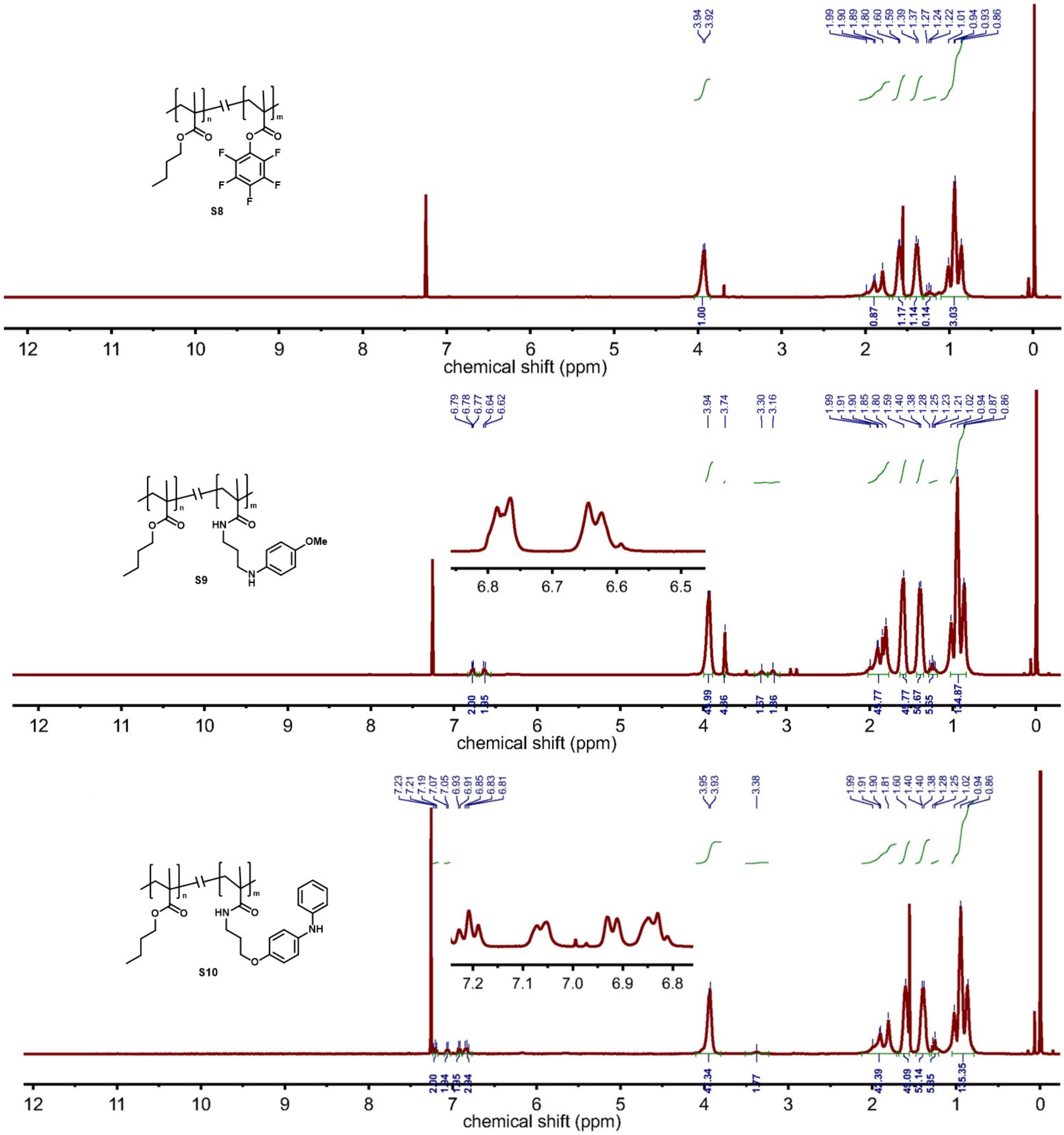

Figure S99: ^{1}H NMR (400 MHz, $CDCl_3$) spectra of activated ester polymer **S8** and amine-modified polymers **S9** and **S10** (top to bottom).

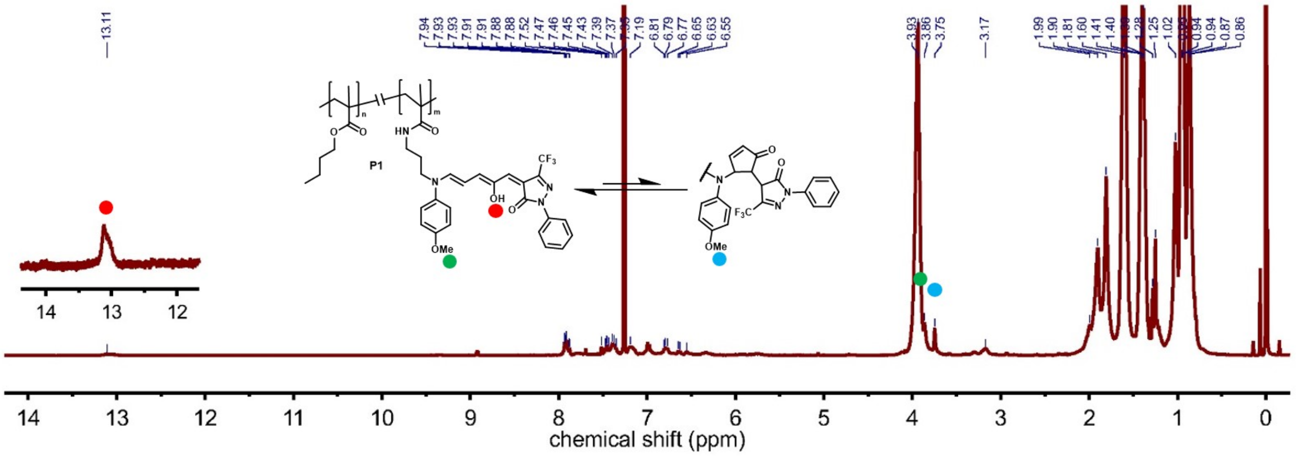

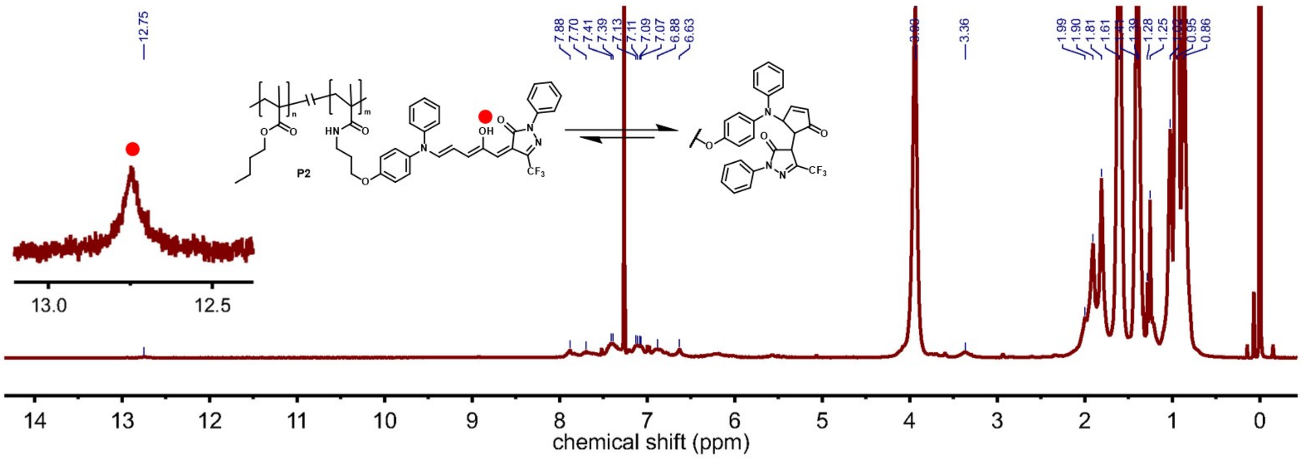

Figure S100: ^{1}H NMR (400 MHz, $CDCl_3$) spectra of DASA-polymers **P1** (top) and **P2** (bottom). In solution the DASAs are in equilibrium between open and closed state resulting in a complex spectrum.[12,16] Characteristic peaks are marked by colored dots.

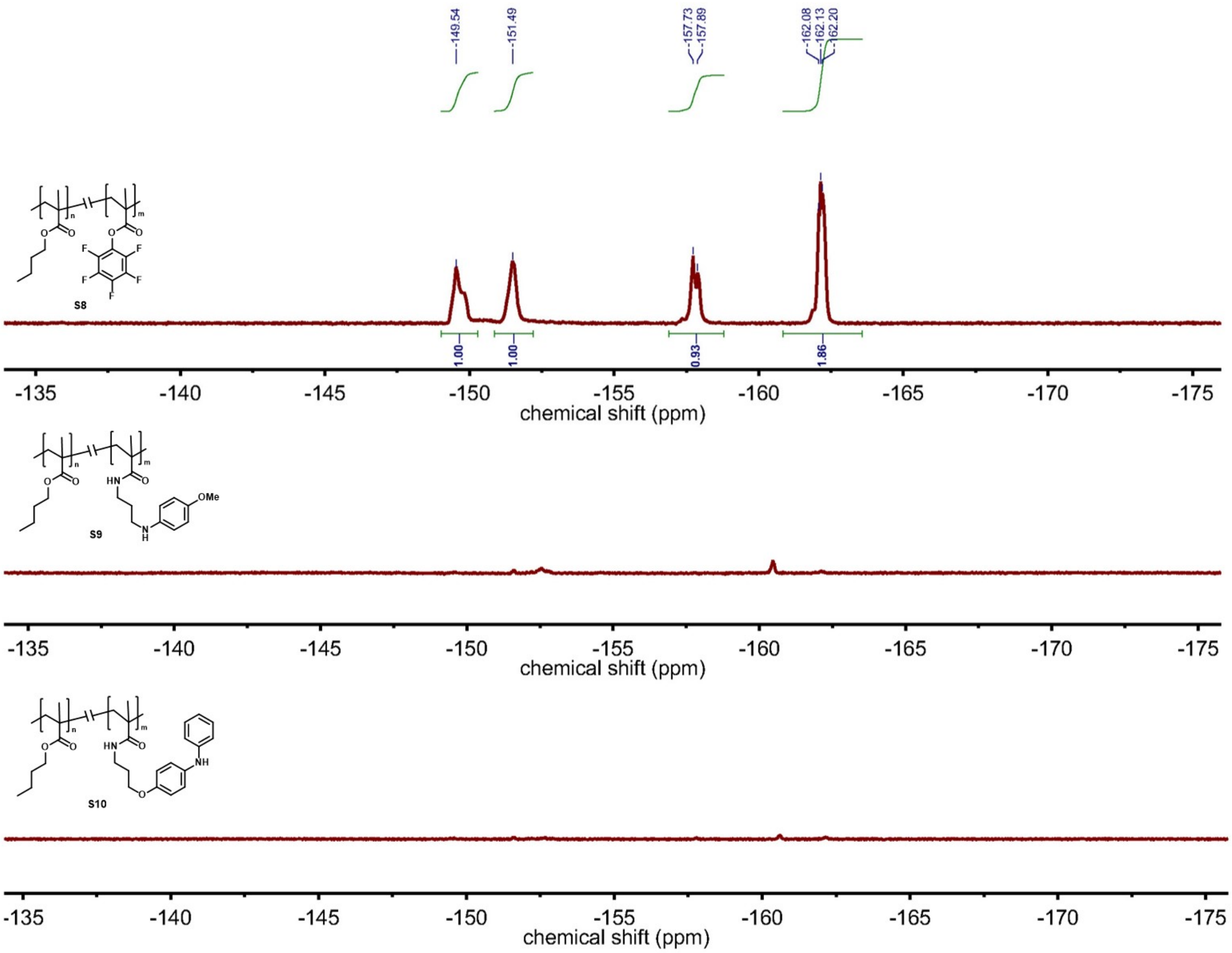

Figure S101: ^{19}F NMR (376 MHz, $CDCl_3$) of **S8**, **S9** and **S10** (top to bottom) showing complete disappearance of the fluorine signals after aminolysis of the pentafluorophenyl ester.

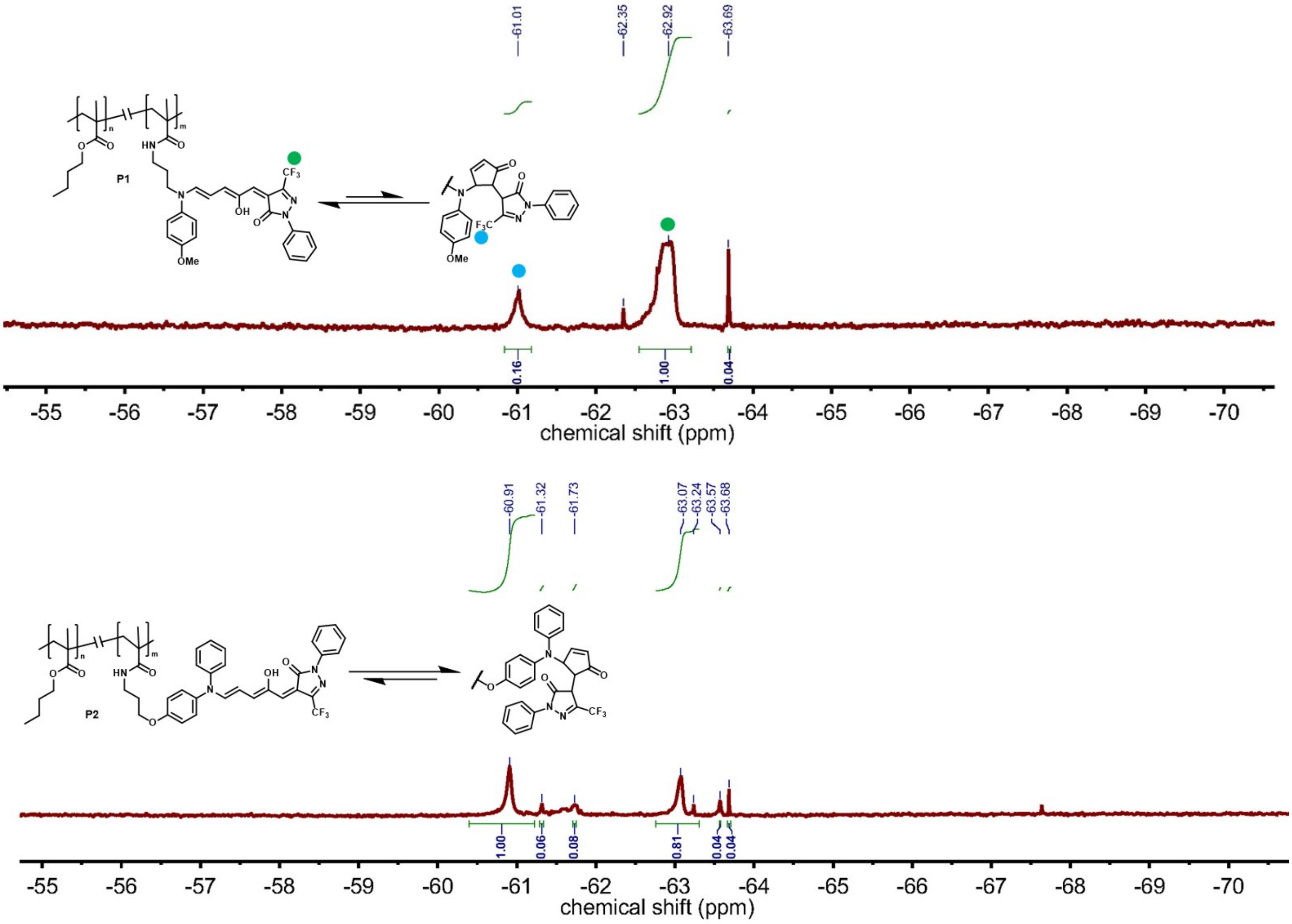

Figure S102: ^{19}F NMR (376 MHz, $CDCl_3$) spectra of DASA-polymers **P1** (top) and **P2** (bottom).

11.2 IR Spectra

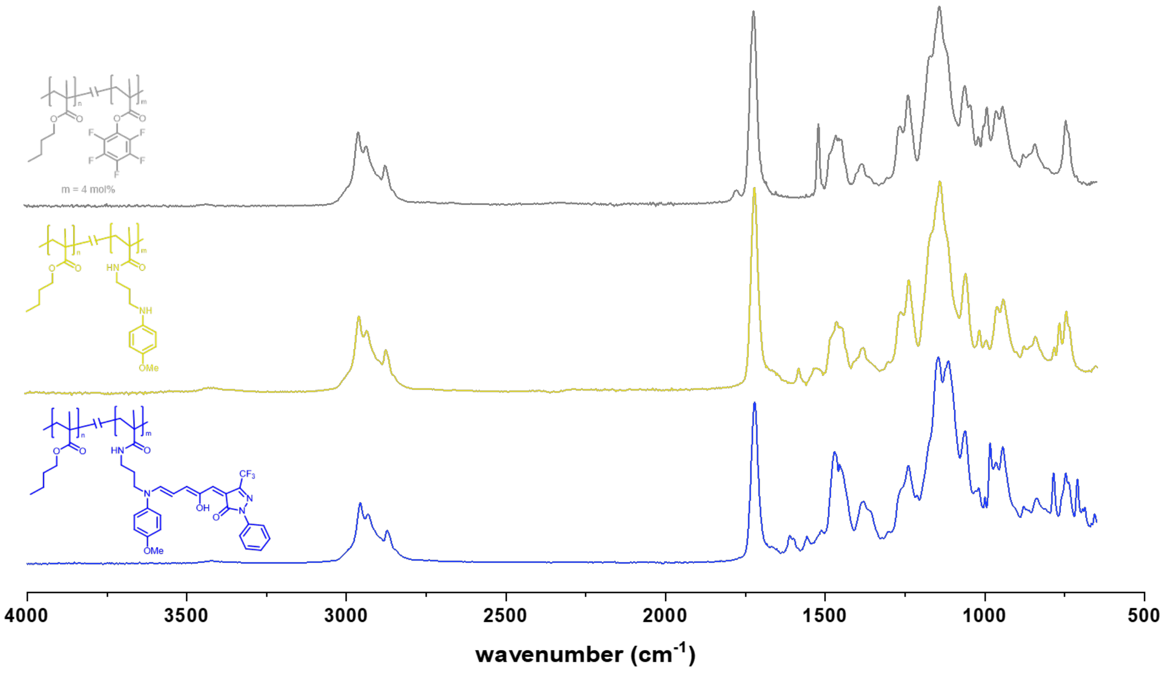

Figure S103: IR absorbance spectra of polymers **S8** (top), **S9** (middle) and **P1** (bottom).

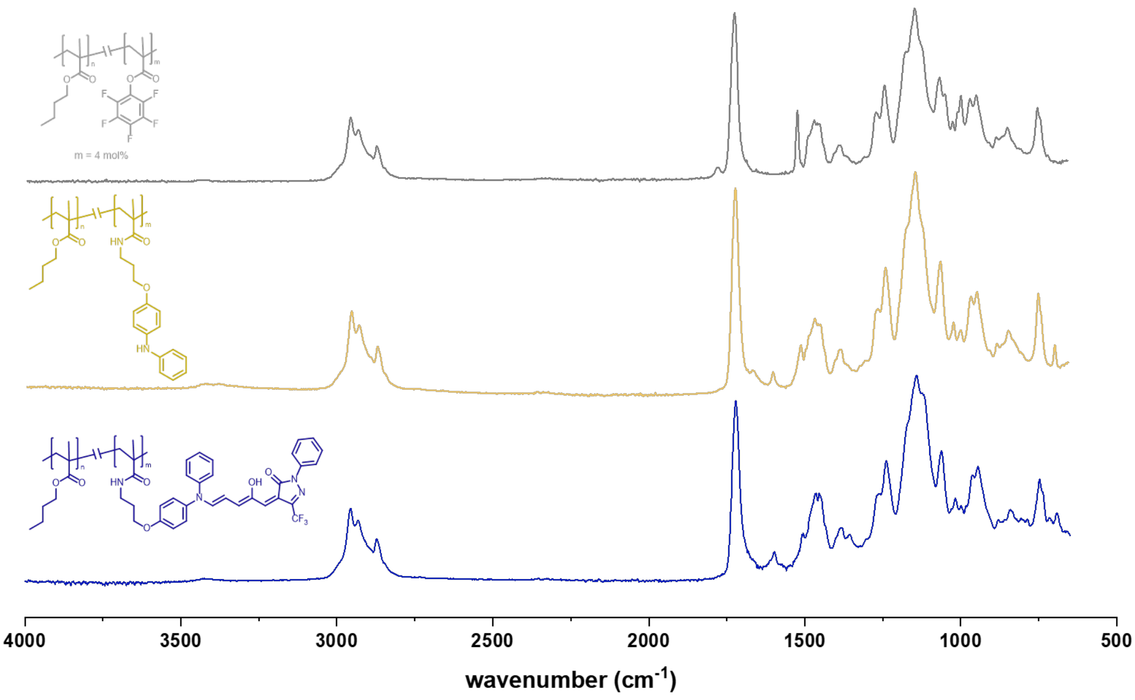

Figure S104: IR absorbance spectra of polymers **S8** (top), **S10** (middle) and **P2** (bottom).

12. References

[1] D. Yu, V. T. Thai, L. I. Palmer, G. K. Veits, J. E. Cook, J. Read de Alaniz, J. E. Hein, *J. Org. Chem.* **2013**, *78*, 12784–12789.
[2] R. F. A. Gomes, J. A. S. Coelho, C. A. M. Afonso, *Chem. - A Eur. J.* **2018**, *24*, 9170–9186.
[3] S. W. Li, R. A. Batey, *Chem. Commun.* **2007**, *8*, 3759–3761.
[4] S. Helmy, S. Oh, F. A. Leibfarth, C. J. Hawker, J. Read de Alaniz, *J. Org. Chem.* **2014**, *79*, 11316–11329.
[5] J. R. Hemmer, Z. A. Page, K. D. Clark, F. Stricker, N. D. Dolinski, C. J. Hawker, J. Read de Alaniz, *J. Am. Chem. Soc.* **2018**, *140*, 10425−10429.
[6] H. Yin, M. Jin, W. Chen, C. Chen, L. Zheng, P. Wei, S. Han, *Tetrahedron Lett.* **2012**, *53*, 1265–1270.

[7] S. Lee, K. Y. Yi, S. K. Kim, J. Suh, N. J. Kim, S. E. Yoo, B. H. Lee, H. W. Seo, S. O. Kim, H. Lim, *Eur. J. Med. Chem.* **2003**, *38*, 459–471.
[8] A. Mastitski, K. Kisseljova, J. Järv, *Proc. Est. Acad. Sci.* **2014**, *63*, 438–443.
[9] S. Helmy, F. A. Leibfarth, S. Oh, J. E. Poelma, C. J. Hawker, J. Read de Alaniz, *J. Am. Chem. Soc.* **2014**, *136*, 8169–8172.
[10] M. M. Lerch, M. Medved, A. Lapini, A. D. Laurent, A. Iagatti, L. Bussotti, W. Szymański, W. J. Buma, P. Foggi, M. Di Donato, B. L. Feringa, *J. Phys. Chem. A* **2018**, *122*, 955–964.
[11] J. R. Hemmer, S. O. Poelma, N. Treat, Z. A. Page, N. D. Dolinski, Y. J. Diaz, W. Tomlinson, K. D. Clark, J. P. Hooper, C. Hawker, J. Read de Alaniz, *J. Am. Chem. Soc.* **2016**, *138*, 13960–13966.
[12] S. Ulrich, J. R. Hemmer, Z. A. Page, N. D. Dolinski, O. Rifaie-Graham, N. Bruns, C. J. Hawker, L. F. Boesel, J. Read de Alaniz, *ACS Macro Lett.* **2017**, *6*, 738−742.
[13] C. Reichardt, *Chem. Rev.* **1994**, *94*, 2319–2358.
[14] M. M. Lerch, M. Di Donato, A. D. Laurent, M. Medved', A. Iagatti, L. Bussotti, A. Lapini, W. J. Buma, P. Foggi, W. Szymański, B. L. Feringa, *Angew. Chemie - Int. Ed.* **2018**, *57*, 8063–8068.
[15] L. Payne, J. D. Josephson, R. S. Murphy, B. D. Wagner, *Molecules* **2020**, *25*, 4928.
[16] O. Rifaie-Graham, S. Ulrich, N. F. B. Galensowske, S. Balog, M. Chami, D. Rentsch, J. R. Hemmer, J. Read de Alaniz, L. F. Boesel, N. Bruns, *J. Am. Chem. Soc.* **2018**, *140*, 8027–8036.
